# Evolution of primate interferon-induced transmembrane proteins (IFITMs): a story of gain and loss with a differentiation into a canonical cluster and IFITM retrogenes

**DOI:** 10.3389/fmicb.2023.1213685

**Published:** 2023-07-26

**Authors:** Luca Schelle, Joana Abrantes, Hanna-Mari Baldauf, Pedro José Esteves

**Affiliations:** ^1^Faculty of Medicine, Max von Pettenkofer Institute and Gene Center, Virology, National Reference Center for Retroviruses, LMU München, Munich, Germany; ^2^CIBIO-InBIO, Research Center in Biodiversity and Genetic Resources, University of Porto, Vairão, Portugal; ^3^BIOPOLIS Program in Genomics, Biodiversity and Land Planning, CIBIO, Vairão, Portugal; ^4^Departamento de Biologia, Faculdade de Ciências, Universidade do Porto, Porto, Portugal; ^5^CITS - Center of Investigation in Health Technologies, CESPU, Gandra, Portugal

**Keywords:** interferon-induced transmembrane proteins, evolution, innate immunity, antiviral proteins, primates, transposable elements, retrogene

## Abstract

Interferon-inducible transmembrane proteins (IFITMs) are a family of transmembrane proteins. The subgroup of immunity-related (IR-)IFITMs is involved in adaptive and innate immune responses, being especially active against viruses. Here, we suggest that IFITMs should be classified as (1) a canonical IFITM gene cluster, which is located on the same chromosome, and (2) IFITM retrogenes, with a random and unique location at different positions within the genome. Phylogenetic analyses of the canonical cluster revealed the existence of three novel groups of primate IFITMs (pIFITM) in the IR-IFITM clade: the prosimian pIFITMs(pro), the new world monkey pIFITMs(nwm) and the old world monkey pIFITMs(owm). Therefore, we propose a new nomenclature: IR-pIFITM1, IR-pIFITM2, IR-pIFITM3, IR-pIFITMnwm, IR-pIFITMowm, and IR-pIFITMpro. We observed divergent evolution for pIFITM5 and pIFITM10, and evidence for concerted evolution and a mechanism of birth-and-death evolution model for the IR-pIFITMs. In contrast, the IFITMs scattered throughout the genomes possessed features of retrogenes retrotransposed by class 1 transposable elements. The origin of the IFITM retrogenes correspond to more recent events. We hypothesize that the transcript of a canonical IFITM3 has been constantly retrotransposed using class 1 transposable elements resulting in the IFITM retro(pseudo)genes. The unique pattern of each species has most likely been caused by constant pseudogenization and loss of the retro(pseudo)genes. This suggests a third mechanism of evolution for the IR-IFITMs in primates, similar to the birth-and-death model of evolution, but via a transposable element mechanism, which resulted in retro(pseudo)genes.

## 1. Introduction

Interferon-inducible transmembrane proteins are relatively small transmembrane proteins with around 130 amino acids (AA). These proteins are encoded by a family of interferon-stimulated genes (ISGs), *IFITM1*, *IFITM2*, and *IFITM3*, which were first discovered as interferon-inducible genes (Friedman et al., 1984), and the paralogs *IFITM5* and *IFITM10*. IFITMs are ancient proteins present in fish, amphibians, reptiles, birds, monotremes, marsupials and mammals (Hickford et al., 2012). Phylogenetically, IFITMs can be divided into three major clades: the immunity-related (IR-)IFITMs (IFITM1, IFITM2, and IFITM3), IFITM5 and IFITM10 (Zhang et al., 2012). IFITMs comprise 5 domains: the N-terminal domain, the CD255 domain, which contains intramembrane domain 1 (IM1) and conserved intracellular loop (CIL), and the C-terminus. The latter consists of intramembrane domain 2 (IM2) and the C-terminal domain (Bailey et al., 2013, 2014). Whether the IMs are intramembrane or rather transmembrane domains remains unclear as their exact topology in the membranes has not been solved and might differ between membrane types (reviewed in Bailey et al., 2014).

IFITMs are associated with several functions: the IR-IFITMs play a role in adaptive (reviewed in Yanez et al., 2020) and innate immune responses, especially against RNA and DNA viruses, with several mechanisms for viral inhibition observed and proposed (extensively reviewed in Diamond and Farzan, 2013; Bailey et al., 2014; Zhao et al., 2018; Liao et al., 2019). IFITM5 has acquired a Ca^2+^ binding site, which is important for its role in osteoblast function and bone mineralization (Hanagata et al., 2011; Hedjazi et al., 2022). The role of IFITM10 remains unclear, but it has recently been associated with gastric cancer (Liu et al., 2021).

Primates diverged into the suborders Strepsirrhini (prosimians) and Haplorrhini ~71.4–77.5 million years ago (MYA). The infraorders Simiiformes and Tarsiiformes (tarsier) originated from Haplorrhini ~61.6–71.1 MYA. At ~40.0–44.2 MYA, the Simiiformes branched to the parvorders of Platyrrhini (new world monkeys) and Catarrhini, which further divided ~26.80–30.60 MYA to Cercopithecidae (old world monkeys) and the superfamily Hominidea (apes), including Hylobatidae (gibbons) and Hominidae (great apes) (divergent times derived from Kumar et al., 2022).

Multigene families were originally believed to evolve by concerted evolution, i.e., the paralog genes would evolve as a unit by genetic exchange from unequal crossing over and gene conversion (Nei and Rooney, 2005). Nei et al. (1997) proposed the birth-and-death model of evolution for multigene families of the immune system where newly duplicated genes are either maintained in the genome and diverge functionally with neofunctionalization or subfunctionalization, or become nonfunctional or are deleted. These models are not mutually exclusive and genes can evolve in a mixed model process (Nei and Rooney, 2005).

Retrogenes or processed pseudogenes are functional retrocopies of genes originating from a parental gene by RNA-based gene duplication via retrotransposition by class 1 transposable elements. Retropseudogenes are the non-functional forms of retrogenes (reviewed in Kaessmann et al., 2009; Troskie et al., 2021). In order to be inherited, retrotransposition has to occur in the germline (Kaessmann et al., 2009). During a retrotransposition event, the mRNA of a parental gene is bound to reverse transcriptase of transposable elements; in mammals, these elements are long interspersed nuclear elements (LINEs), which recognize polyadenylated mRNA (Doucet et al., 2015). The bound mRNA is then retrotransposed to another genomic localization and integrated at a consensus cleavage site of the endonuclease by a process termed target-site primed reverse transcription (TPRT) (Luan et al., 1993; Troskie et al., 2021). Retropseudogenes are characterized by the lack of introns, and the presence of a conserved poly A signal (AATAAA), a poly A tail start and target-site duplications [5′ and 3′ untranslated region (UTR)] (Esnault et al., 2000; Kaessmann et al., 2009). The possible fate of retro(pseudo)genes has been reviewed by Troskie et al. (2021), and includes, for example, the acquisition of a promoter and expression, neofunctionalization, development of a non-coding regulatory function and degeneracy.

Some studies have addressed primate IFITM evolution (Hickford et al., 2012; Zhang et al., 2012; Compton et al., 2016; Wilkins et al., 2016; Benfield et al., 2020). In this study, we conducted a more in-depth study of IFITM evolution in primates by including more primate species (Rahman and Compton, 2021) into the analyses and considering the separation of canonical IFITMs cluster and IFITM retrogenes.

## 2. Results

### 2.1. Gene synteny of canonical *IFITM* cluster in primates

**Figure 1:**
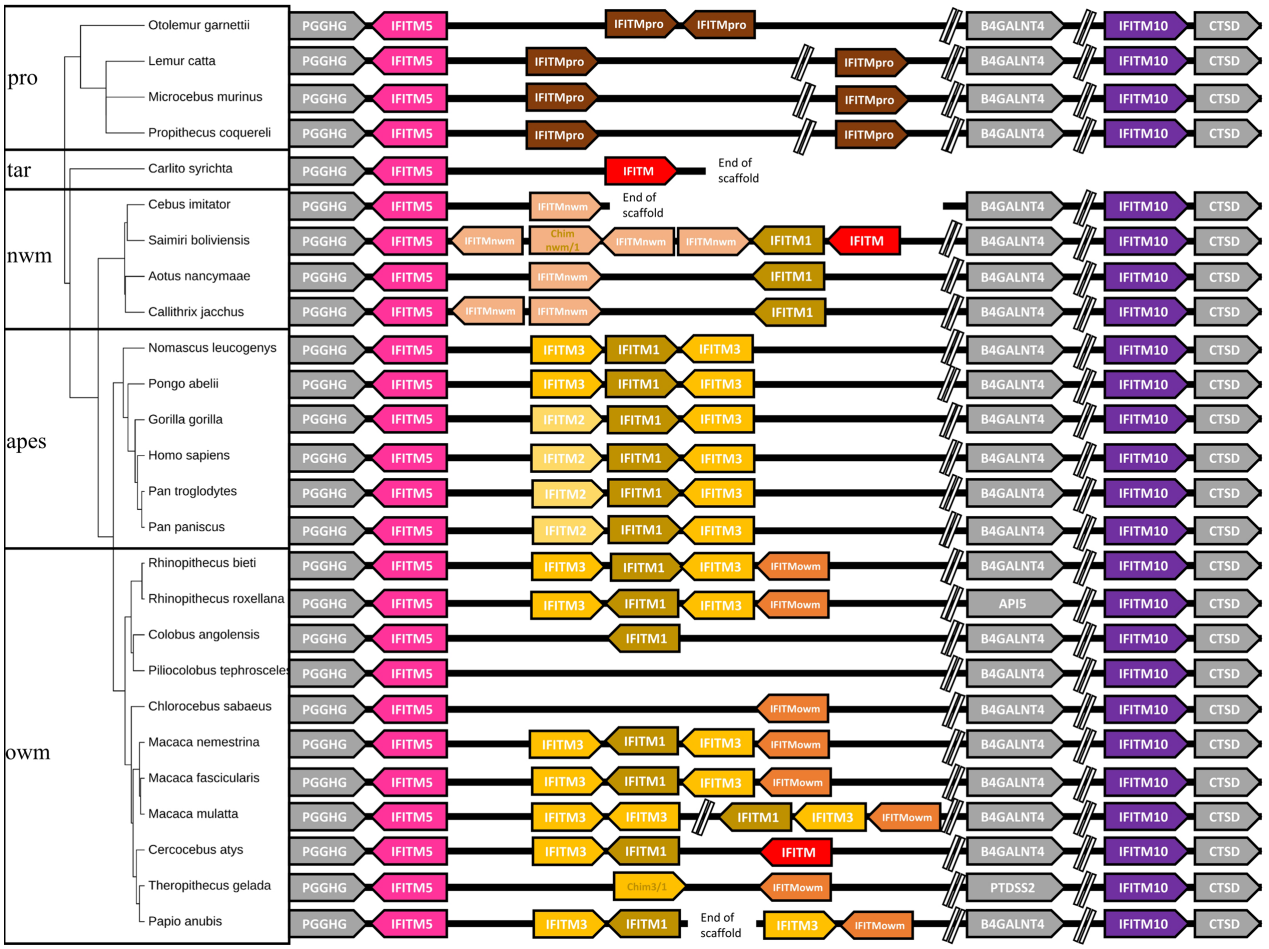
Gene synteny of primate *IFITMs* of the canonical cluster. The gene synteny of the primate *IFITMs* in the canonical cluster is displayed for the 26 analyzed primate species (right). *IFITMs* were colored following the grouping in the phylogenetic analyses (Figure 3). Arrows indicate gene orientation. Primate phylogeny (left) was constructed using timetree.org (Kumar et al., 2022). Gray: flanking genes, pink: *IFITM5*, purple: *IFITM10*, brown: *IR-pIFITMpro*, light orange: *IR-pIFITMnwm*, orange: *IR-pIFITMowm*, sand: *IR-pIFITM1*, yellow: *IR-pIFITM3*, light yellow: *IR-pIFITM2* red: not considered in the analyses, e.g., partial mRNA, Chim: Chimeric genes (see below); pro: prosimians; nwm: new world monkeys; owm: old world monkeys.

After retrieving all available primate *IFITM* sequences from the NCBI database (Accession numbers of the sequences are listed in Supplementary Table S1), we inferred the gene synteny, which is depicted in Figure 1 (right side). Genes used for synteny were located on the same chromosome or same unplaced scaffold in each species and were all flanked by the same genes (PGGHG, BAGALNT4, CTSD respectively; in gray in Figure 1), except for the *IFITMs* of *Rhinopithecus roxellana* and *Theropithecus gelada*, which were not flanked by *BAGALNT4* due to chromosomal rearrangements. This prompted us to term them the canonical IFITM cluster. Genes in red could not be aligned or were only partial mRNAs or pseudogenes, and were therefore excluded from the alignment (Figure 1).

For all the 26 species included, we observed that IFITM5 and IFITM10 consisted of single-copy genes at a conserved position in the synteny. The IR-IFITMs gene synteny was also conserved in the prosimians and apes; however, prosimians possessed two *IR-IFITMs*, with a distinct gene location and orientation rearrangement compared to *Otolemur garnettii* (Figure 1). The apes had three identically arranged *IR-IFITMs*, i.e., one more than the prosimians from which they separated around ~74 MYA (Kumar et al., 2022). For the new and old world monkeys, different numbers of *IR-IFITM* genes were observed, ranging from zero to six (Figure 1). We could not exclude that, especially in the case of single *IR-IFITMs,* additional genes might have been missed due to small size of the gene, gaps in scaffolds and/or poorer genome quality (Figure 1). In summary, we observed diversification of the gene copy number of the *IR-IFITMs* and their synteny in the apes, new and old world monkeys since the separation from the prosimians. In contrast, *IFITM5* and *IFITM10* appeared highly conserved as single copy genes present at a fixed location.

### 2.2. Distinction between canonical IFITMs cluster and IFITM retrogenes

**Figure 2:**
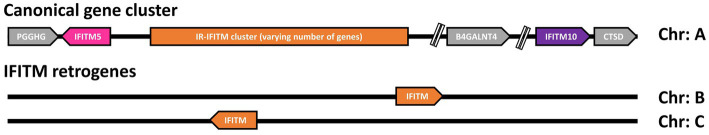
General genomic arrangement of canonical *IFITMs* cluster and *IFITMs* retrogenes. Schematic representation of the general arrangement of the proposed canonical gene cluster and *IFITM* retrogenes. The distinction between the consistently arranged canonical *IFITM* cluster on one chromosome (Chr) and the *IFITM* retrogenes, which are randomly distributed throughout the genome, is shown. Arrows indicate gene orientation. Gray: flanking genes, pink: *IFITM5*, purple: *IFITM10*, orange: *IR-IFITMs.*

For most of the primate species analyzed, in addition to the canonical cluster, we found various *IFITMs scattered* at different random positions within the genome, with most having a unique localization. In line with our observations that these genes are retrogenes (see Section 2.7), we propose that primate *IFITMs* can be classified according to their localization in the genome into canonical *IFITMs* cluster and *IFITM* retrogenes (Figure 2).

### 2.3. Phylogeny of canonical IFITM cluster in primates

**Figure 3:**
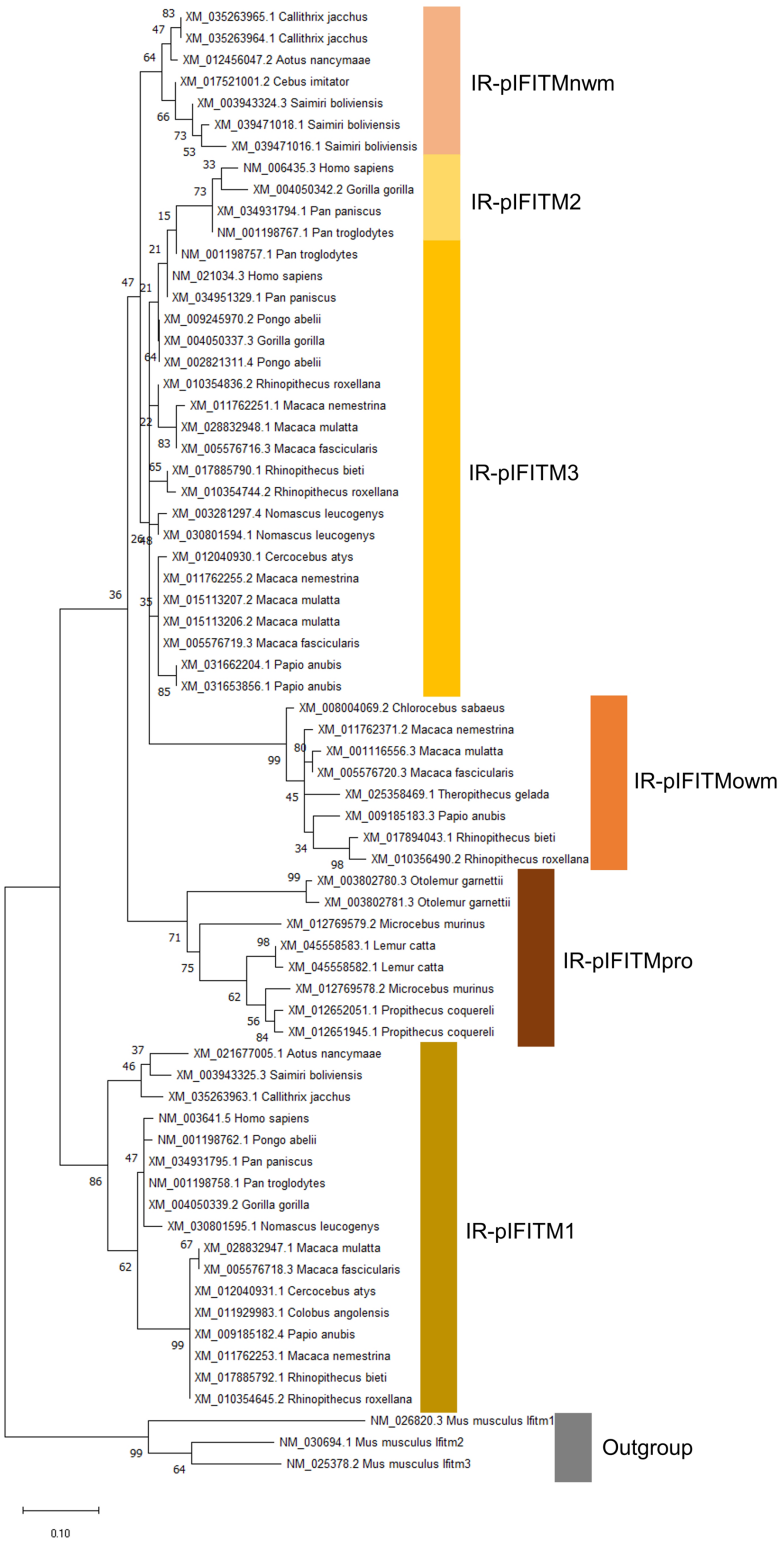
Phylogeny of IR-IFITM in 26 primate species based on AA sequences. The evolutionary history was inferred by using the Maximum Likelihood (ML) method. The tree is drawn to scale, with branch lengths measured in the number of substitutions per site. The bootstrap value is shown next to the branches. Mouse Ifitms were used as outgroup. IR-pIFITM1, IR-pIFITM2, IR-pIFITM3 (Immunity-Related-primate), IR-pIFITMnwm (Immunity-Related-primate-new world monkey) IR-pIFITMowm (Immunity-Related-primate-old world monkey), and IR-pIFITMpro (Immunity-Related-primate-prosimian).

For phylogenetic inference, only the IFITMs from the canonical cluster were used (Figure 3).

Considering the IR-IFITMs of primates (IR-p; Figure 3, accession numbers Supplementary Table S1, alignment Supplementary Figure S5), IR-pIFITM1 was only present in Simiiformes, while absent in prosimians, and formed a well-supported separate group in accordance with the primate phylogeny. The absence of IR-pIFITM1 in prosimians was unique for primates. The genes classified as *IR-pIFITM3s* did not cluster in accordance to primate phylogeny and appeared to be polyphyletic. The IR-pIFITM2 sequences clustered together (bootstrap value of 73), but they were only present in *Homo sapiens*, *Gorilla gorilla*, *Pan paniscus*, and *Pan troglodytes*. We also observed three new phylogenetic groups of primate IFITMs: one of the clusters comprised all prosimian IFITMs (pIFITM(pro)), the second included only old world monkeys IFITMs (pIFITM(owm)) and the third encompassed all NCBI annotated IFITM3 of new world monkeys (pIFITM(nwm)). Except for *Colobus angolensis* and *Piliocolobus tephrosceles*, all old world monkeys maintained a copy of the pIFITMowm, which is in addition to the pIFITM3s present in old world monkeys.

Regarding the phylogeny of the pIFITM5 (Supplementary Figure S1, accession numbers Supplementary Table S1, alignment Supplementary Figure S2) and pIFITM10 (Supplementary Figure S3, accession numbers Supplementary Table S1, alignment Supplementary Figure S4), clustering was according to the established primate phylogeny (Figure 1). The primate IFITM5s were highly conserved, with 72% (97/134) of the sites 100% conserved in all aligned species. The same applied for primate IFITM10s where 88% (115/130) of the sites were 100% identical. Indeed, the *IFITM5* and *IFITM10* genes of prosimians and tarsier, new world monkeys, old world monkeys and apes clustered into closely related separate groups, with the exception of IFITM5 of *Macaca* species (Supplementary Figure S1). This was most likely caused by a point mutation leading to an amino acid exchange (G19R), compared to the otherwise identical sequences of old world monkey IFITM5s (Supplementary Figure S2).

### 2.4. Sequence characteristics of primate IR-IFITM groups

**Figure 4:**
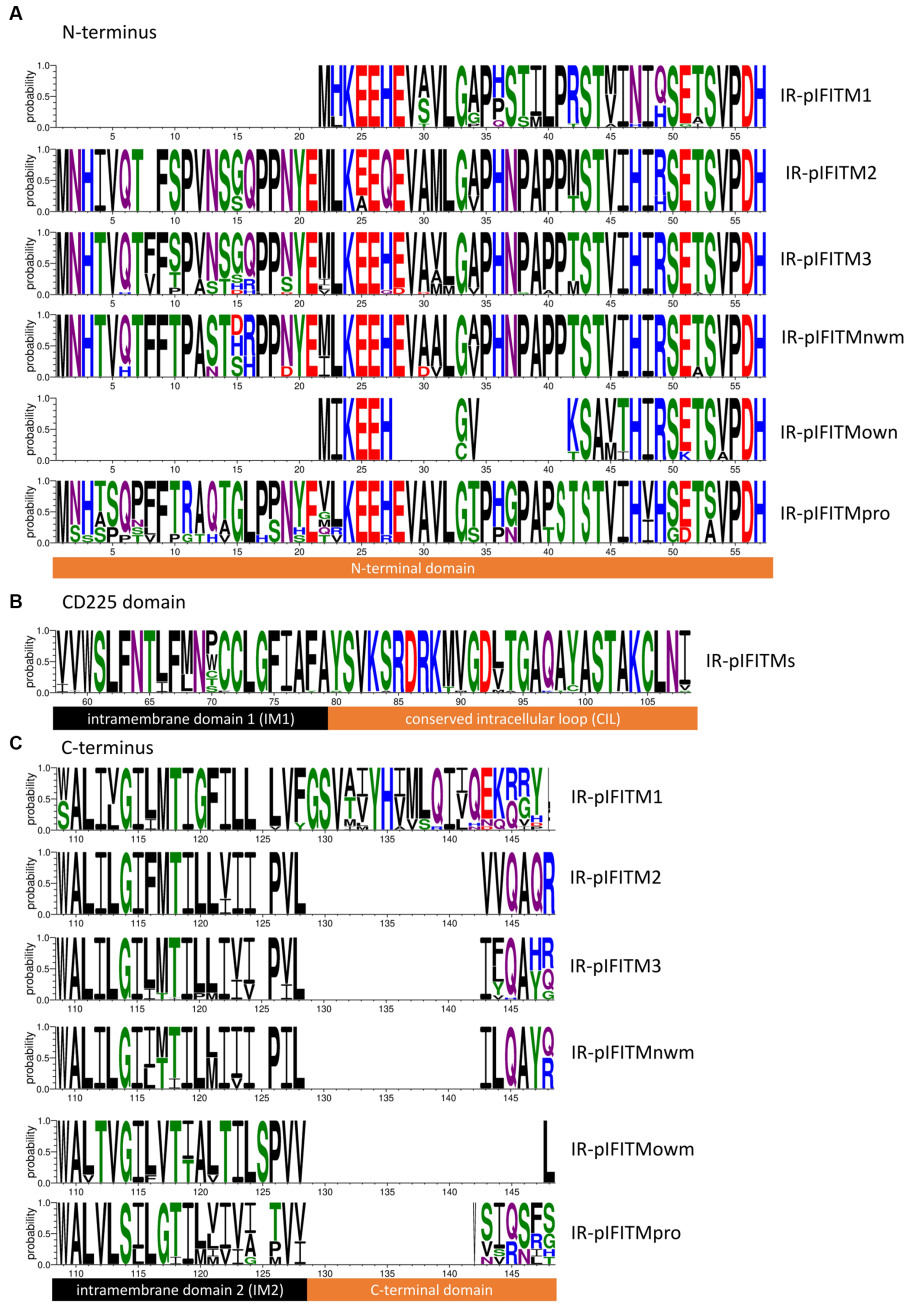
AA sequence characteristics of primate IFITM groups. Sequence logos were derived from the AA alignments of the primate IFITM groups (Supplementary Figures S6–S8) defined in Figure 3 (IR-pIFITM1, IR-pIFITM2, IR-pIFITM3, IR-pIFITMnwm, IR-pIFITMowm, and IR-pIFITMpro). **(A)** N-termini with variable lengths. **(B)** Highly conserved CD225 domains comprising IM1 domain and CIL. **(C)** C-termini including IM2 and the C-terminal domains with highly variability in length. Probability of residues is shown. Protein domains are indicated. Black: transmembrane/intramembrane domain, orange: topological domain. Logos were generated using WebLogo3 (Crooks et al., 2004).

To further characterize and classify the six proposed groups of primate IR-IFITMs, we investigated the AA sequences of the N-termini (Figure 4A), the CD225 middle domains (Figure 4B), and the C-termini (Figure 4C). The CD225 domain sequence was based on the alignment of all six groups, because they were highly conserved except for two AAs (Figure 4B).

We observed that the groups could be characterized by their N- and C-termini (Figure 4), as the remaining CD225 domains were highly conserved and not informative. IR-pIFITM1 and IR-pIFITMowm had shorter N-termini (20-21 AA) compared to IR-pIFITM2/3/nwm/pro, while IR-pIFITMowm also had small deletions next to the start codon (5 AA and 7 AA). The IR-pIFITM2/3/nwm/pro N-termini were of the same length, except that IRpIFITM2 had a deletion of one AA. The N-termini of IR-pIFITM2/3/nwm showed higher similarity to each other than to IR-pIFITMpro, but differed especially at positions 4–16 and 27 (Figure 4A). The IM2 domain of the C-terminus was less conserved than the CD225 domain and therefore a further determinant of the six groups, but the IMs of IR-pIFITM2/3/nwm were more similar. The C-terminal domains differed between the groups in length and sequence. IR-pIFITM1s had an elongated C-terminal domain, while the domain was lost in IR-pIFITMowm. IR-pIFITM2/3/nwm/pro had C-terminal domains of the same length but differed in sequence (Figure 4C). In summary, all primate IR-IFITM groups comprised a highly conserved CD225; yet, they can be differentiated and classified by their N- and C-termini, which were group-specific both in terms of sequence and length.

### 2.5. New classification of primate IFITMs

Based on our analyses, we propose a new nomenclature for the primate *IR-IFITMs* as *IR-pIFITM1*, *IR-pIFITM2*, *IR-pIFITM3* (Immunity-Related-primate), *IR-pIFITMnwm* (Immunity-Related-primate-new world monkey) *IR-pIFITMowm* (Immunity-Related-primate-old world monkey) and *IR-pIFITMpro* (Immunity-Related-primate-prosimian). The old and new nomenclature is listed in Table 1. This phylogeny-based proposed nomenclature does not specify individual genes in a species if more than one gene is present. Due to the closer relationship between paralogs of a species, caused by concerted evolution, than to orthologs, a relationship-based specification was not possible. Therefore, we suggest to specify them according to their synteny as locus (L) + number (1, 2, 3…) = L1, L2, L3… without emphasizing any phylogenetic or functional relationship.

**Table 1:** New proposed classification of primate IR-pIFITMs.

Primate group	Primate	Accession number	Old classification	New classification
New world monkeys	*Callithrix jacchus*	XM_035263965.2	*IFITM3*	*IR-pIFITMnwm*
	*Callithrix jacchus*	XM_035263964.2	*IFITM3*	
	*Aotus nancymaae*	XM_012456047.2	*IFITM3*	
	*Cebus imitator*	XM_017521001.2	*IFITM3*	
	*Saimiri boliviensis*	XM_003943324.3	*IFITM3*	
	*Saimiri boliviensis*	XM_039471018.1	*IFITM3*	
	*Saimiri boliviensis*	XM_039471016.1	*IFITM3*	
Great apes	*Homo sapiens*	NM_006435.3	*IFITM2*	*IR-pIFITM2*
	*Gorilla gorilla*	XM_004050342.2	*IFITM2*	
	*Pan paniscus*	XM_034931794.1	*IFITM2*	
	*Pan troglodytes*	NM_001198767.1	*IFITM2*	
	*Pan troglodytes*	NM_001198757.1	*IFITM3*	*IR-pIFITM3*
	*Homo sapiens*	NM_021034.3	*IFITM3*	
	*Pan paniscus*	XM_034951329.1	*IFITM3*	
	*Pongo abelii*	XM_009245970.2	*IFITM3*	
	*Gorilla gorilla*	XM_004050337.3	*IFITM3*	
	*Pongo abelii*	XM_002821311.5	*IFITM3*	
Old world monkeys	*Rhinopithecus roxellana*	XM_010354836.2	*IFITM3*	
	*Macaca nemestrina*	XM_011762251.1	*IFITM3*	
	*Macaca mulatta*	XM_028832948.1	*IFITM3*	
	*Macaca fascicularis*	XM_005576716.3	*IFITM3*	
	*Rhinopithecus bieti*	XM_017885790.1	*IFITM3*	
	*Rhinopithecus roxellana*	XM_010354744.2	*IFITM3*	
Gibbon	*Nomascus leucogenys*	XM_003281297.4	*IFITM3*	
	*Nomascus leucogenys*	XM_030801594.1	*IFITM3*	
Old world monkeys	*Cercocebus atys*	XM_012040930.1	*IFITM3*	
	*Macaca nemestrina*	XM_011762255.2	*IFITM3*	
	*Macaca mulatta*	XM_015113207.2	*IFITM3*	
	*Macaca mulatta*	XM_015113206.2	*IFITM3*	
	*Macaca fascicularis*	XM_005576719.3	*IFITM3*	
	*Papio anubis*	XM_031662204.1	*IFITM3*	
	*Papio anubis*	XM_031653856.1	*IFITM3*	
Old world monkeys	*Chlorocebus sabaeus*	XM_008004069.2	*IFITM3*	*IR-pIFITMowm*
	*Macaca nemestrina*	XM_011762371.2	*IFITM2*	
	*Macaca mulatta*	XM_001116556.3	*IFITM3*	
	*Macaca fascicularis*	XM_005576720.3	*IFITM3*	
	*Theropithecus gelada*	XM_025358469.1	*IFITM3*	
	*Papio anubis*	XM_009185183.3	*IFITM3*	
	*Rhinopithecus bieti*	XM_017894043.1	*IFITM3*	
	*Rhinopithecus roxellana*	XM_010356490.2	*IFITM3*	
Prosimians	*Otolemur garnettii*	XM_003802780.3	*IFITM3*	*IR-pIFITMpro*
	*Otolemur garnettii*	XM_003802781.3	*IFITM3*	
	*Microcebus murinus*	XM_012769579.2	*IFITM3*	
	*Lemur catta*	XM_045558583.1	*IFITM3*	
	*Lemur catta*	XM_045558582.1	*IFITM3*	
	*Propithecus coquereli*	XM_012652051.1	*IFITM3*	
	*Propithecus coquereli*	XM_012651945.1	*IFITM3*	
New world monkeys	*Aotus nancymaae*	XM_021677005.1	*IFITM1*	*IR-pIFITM1*
	*Saimiri boliviensis*	XM_003943325.3	*IFITM1*	
	*Callithrix jacchus*	XM_035263963.1	*IFITM1*	
Apes	*Homo sapiens*	NM_003641.5	*IFITM1*	
	*Pongo abelii*	NM_001198762.1	*IFITM1*	
	*Pan paniscus*	XM_034931795.1	*IFITM1*	
	*Pan troglodytes*	NM_001198758.1	*IFITM1*	
	*Gorilla gorilla*	XM_004050339.2	*IFITM1*	
	*Nomascus leucogenys*	XM_030801595.1	*IFITM1*	
Old world monkeys	*Macaca mulatta*	XM_028832947.1	*IFITM1*	
	*Macaca fascicularis*	XM_005576718.3	*IFITM1*	
	*Cercocebus atys*	XM_012040931.1	*IFITM1*	
	*Colobus angolensis*	XM_011929983.1	*IFITM1*	
	*Papio anubis*	XM_009185182.4	*IFITM1*	
	*Macaca nemestrina*	XM_011762253.1	*IFITM1*	
	*Rhinopithecus bieti*	XM_017885792.1	*IFITM1*	
	*Rhinopithecus roxellana*	XM_010354645.2	*IFITM1*	

Shown is the old and proposed new classification of primate IFITMs. Order corresponds to phylogenetic tree (Figure 3).

### 2.6. IR-pIFITM1/3 chimeras

**Figure 5:**
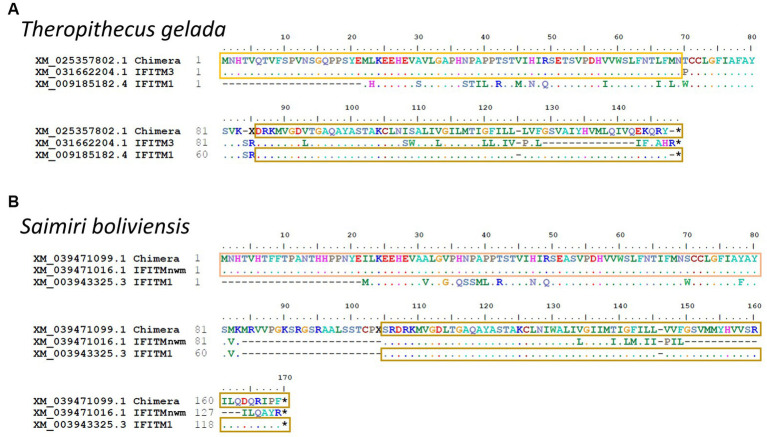
Identification of IR-pIFITM3/1 and IR-pIFITMnwm/1 chimeras. Alignment of chimeric IR-pIFITMs of *Theropithecus gelada* (owm) **(A)** and *Saimiri boliviensis* (nwm) **(B)** with IR-pIFITM3/nwm and IR-pIFITM1. For *Theropithecus gelada*, the alignment was performed with the protein sequences from its closest relative *Papio anubis* as only the chimeric gene is present in the genome. For *Saimiri boliviensis*, X represents start of frameshift omitted to emphasize identity of C-terminus. Identity to IR-pIFITM3/nwm highlighted with yellow or light orange box, respectively, and identity to IR-pIFITM1 highlighted with sand box.

In *Theropithecus gelada* and *Saimiri boliviensis*, we found longer IFITMs sequences that did not align with either of the six primate groups. The alignment of these IFITMs revealed two chimeric sequences with recombination between an IR-pIFITM3/nwm at the N-termini and an IR-pIFITM1 at the C-termini (Figure 5).

### 2.7. Genomic localization of additional primate IFITMs

We observed that the many additional IFITMs were not localized in the canonical clusters, but rather spread throughout the genome. In prosimians, only one additional IFITM was present in *Otolemur garnettii*. For the remaining primates, variable numbers of additional IFITMs were detected, ranging from 6 to 21 genes (Table 2). We further noted an increased number of these additional IFITMs after the separation of prosimians from all other primates.

**Table 2:** Number of IFITM retrogenes in primate species.

	Species	No. of additional IFITMs
Apes	*Homo sapiens*	12
	*Pan paniscus*	5
	*Pan troglodytes*	5
	*Gorilla gorilla*	3
	*Pongo abelii*	4
	*Nomascus leucogenys*	5
Old world monkeys	*Cercocebus atys*	10
	*Chlorocebus sabaeus*	14
	*Macaca fascicularis*	7
	*Macaca mulatta*	8
	*Macaca nemestrina*	8
	*Papio anubis*	11
	*Theropithecus gelada*	11
	*Colobus angolensis*	12
	*Piliocolobus tephrosceles*	8
	*Rhinopithecus bieti*	6
	*Rhinopithecus roxellana*	8
New world monkeys	*Aotus nancymaae*	10
	*Cebus imitator*	26
	*Callithrix jacchus*	28
	*Saimiri boliviensis*	20
Tarsier	*Carlito syrichta*	7
Prosimians	*Microcebus murinus*	0
	*Propithecus coquereli*	0
	*Otolemur garnettii*	1
	*Lemur catta*	0

Shown is the number of IFITM retrogenes in the respective species.

For 13 selected species, covering apes (all apes), old and new world monkeys (randomly selected representatives), tarsier (only one genome available) and prosimians (only one species with additional IFITM), we mapped the scattered IFITMs to characterize their synteny (Supplementary Table S2). The genomic localization of the scattered IFITMs appeared random and unique. Further, we observed that a considerable number was located in the intronic regions of other genes, especially in new and old world monkeys (Table 3). Only in closely related species, we observed a genomic overlap, with some IFITMs present in more than one species flanked by the same genes (mostly among apes, some among old world monkeys, one among new world monkeys and none for tarsier and prosimians; Table 4).

**Table 3:** IFITM retrogenes in primate species with location in introns of other genes.

No.	Species and location	Direction	Flanking gene	Direction	Annotated	Accession number	Direction	Flacking gene	In intron	Direction	Gene
5	Chr. 12	>	SH2B3	>	IFITM3P5 pseudo	NG_006225.2	<	BRAP	in	<	ATXN2
6	Chr. 12	<	AMIGO2	<	IFITM3P6 pseudo	NG_006230.2	<	RPAP3	in	>	PCED1B
	*Pan paniscus*										
2	Chr. 12	>	SH2B3	>	IFITM3P5 pseudo	XM_034934516.1	<	BRAP	in	<	ATXN2
	*Macaca mulatta*										
1	Chr. 16	<	CTC1	<	3 like 8 (owm P1)	XM_001112566.4	>	RANGRF	in	>	PFAS
2	Chr. 1	>	ATP5PB	>	3 like (owm P2)	XM_001106166.4	>	RAP1A	in	<	TMIGD3
4	Chr. 10	<	SLC25A17	>	3 ps (owm P4)	XR_001447798.2	>	XPNPEP3	in	<	ST13
6	Chr. 9	<	OIT1	>	3 ps	XR_001447146.2	>	MICU1	in	<	MCU
7	Chr. 11	>	SH2B3	>	P5	XR_001448216.2	<	BRAP	in	<	ATXN2
	*Cercocebus atys*										
2	Unplaced	>	ATP5PB	>	3 like (owm P2)	XR_001017992.1	>	RAP1A	in	<	TMIGD1
3	Unplaced		End	>	3 ps	XR_001010903.1	>	TAB3	in	>	dystropine-like
4	Unplaced	<	CTC1	<	3 like 8 (owm P1)	XM_012051598.1	>	RANGRF	in	>	PFAS
6	Unplaced	<	SLC25A17	>	3 ps (owm P4)	XR_001010110.1	>	XPNPEP3	in	<	ST13
8	Unplaced	>	SH2B3	>	P5	XR_001010401.1	<	BRAP	in	<	ATXN2
	*Rhinopithecus roxellana*										
1	Chr. 12	<	WTIP	>	3 ps	XR_747609.2	<	PDCD2L	in	<	UBA2
2	Chr. 19	<	C1QL1	>	3 like	XM_030922644.1	>	NMT1	in	<	DCAKD
4	Chr. 10	>	WASHC4	>	3 ps	XR_004059464.1	<	NUAK1	in	<	APPL2
5	Chr. 5	<	ATP10A	>	3 ps	XR_004057469.1	>	GABRB5	in	<	GABRB3
6	Chr. 13	<	SLC25A17	>	3 ps	XR_004052498.1	>	XPNPEP3	in	<	ST13
	*Aotus nancymaae*										
3	Unplaced	>	EGF	>	3 ps	XR_001104807.2	>	ENPEP	in	<	ELOVL6
	*Saimiri boliviensis*										
6	Unplaced	<	RPH3a	>	3 like	XM_039472141.1	>	RPL6	in	<	PTPN11
9	Unplaced	<	MTERF1	>	3 like	XM_039473093.1	<	CYP51A1	in	>	AKAP9
15	Unplaced	>	LRRFIP2	>	3 like	XM_039461847.1	>	EPM2AIP1	in	<	MLH1
19	Unplaced	<	KIAA1586	>	3 like	XM_039467413.1	>	DST	in	<	BEND6
	*Carlito syrichta*										
1	Unplaced	>	SVOPL	>	3 ps	XR_504221.2	End		in	<	Trim24
6	Unplaced	>	SPINK4	>	3 ps	XM_008074487.1	>	CHMP5	in	<	BAG1

in - located in introns.

**Table 4:** IFITM retrogenes present in more than one primate species.

No.	Species and location	Direction	Flanking gene	Direction	Annotated	Accession number	Direction	Flacking gene	In intron	Direction	Gene
1	Chr. 4	<	EHPA5	>	IFITM3P1 pseudo	NG_006204.1	<	CENPC	x		
2	Chr. 12	<	AMN1	>	IFITM3P2 pseudo	NG_006205.3	>	RESF1	x		
3	Chr. 6	<	SPDEF	<	IFITM3P3 pseudo	NG_006229.1	<	ILRUN	x		
4	Chr. 7	<	NUPR2	>	IFITM3P4 pseudo	NG_006223.3	<	ZNF479	x		
5	Chr. 12	>	SH2B3	>	IFITM3P5 pseudo	NG_006225.2	<	BRAP	in	<	ATXN2
6	Chr. 12	<	AMIGO2	<	IFITM3P6 pseudo	NG_006230.2	<	RPAP3	in	>	PCED1B
7	Chr. 1	>	SYF2	>	IFITM3P7 pseudo	NG_006227.1	>	RUNX3	x		
8	Chr. 8	>	CDH7	>	IFITM3P8	NG_006224.1	>	CLVS1	x		
9	Chr. 2	>	PAPLOG	>	IFITM3P9	NG_006228.3	>	BCL11A	x		
10	Chr. 6	>	HLA-F	<	IFITM4p	NR_001590.1	>	HLA-G	x		
11	Chr.8	>	YTHDF3	>	IFITM8P pseudo	NG_005307.4	>	BHLHE22	x		
12	Chr. 11	>	MYEOV	<	IFITM9P pseudo	NG_006210.1	>	CCND1	x		
	*Pan paniscus*										
1	Chr. 12	<	AMN1	>	IFITM3P2 pseudo	XM_003813732.5	>	RESF1	x		
2	Chr. 12	>	SH2B3	>	IFITM3P5 pseudo	XM_034934516.1	<	BRAP	in	<	ATXN2
3	Chr. 2a	>	PAPLOG	>	IFITM3P9	XM_034953202.1	>	BCL11A	x		
4	Chr. 6	>	HLA-F related	<	IFITM4p	XM_034961680.1	>	HLA-G	x		
5	Chr.8	>	YTHDF3	>	IFITM8P pseudo	XM_034966156.1	>	BHLHE22	x		
	*Pan troglodytes*										
1	Chr. 4	<	EHPA5	>	IFITM3P1 pseudo	XR_001716631.2	<	CENPC	x		
2	Chr. 12	<	AMN1	>	IFITM3P2 pseudo	XM_003952225.4	>	RESF1	x		
3	Chr. 7	<	NUPR2	>	IFITM3P4 pseudo	XR_169790.4	<	ZNF479	x		
4	Chr. 2a	>	PAPLOG	>	IFITM3P9	XR_001715794.1	>	BCL11A	x		
5	Chr. 6	>	HLA-F related	<	IFITM4p	XR_002944366.1	>	HLA-G	x		
	*Gorilla gorilla*										
1	Chr. 12	<	AMN1	>	IFITM3P2 pseudo	XM_004052942.3	>	RESF1	x		
2	Chr. 2a	>	PAPLOG	>	IFITM3P9	XR_002004539.2	>	BCL11A	x		
	*Pongo abelii*										
1	Chr. 12	<	AMN1	>	IFITM3P2 pseudo	XR_656249.2	>	RESF1	x		
2	Chr. 7	<	NUPR2	>	IFITM3P4 pseudo	XR_002913425.1	<	ZNF479	x		
3	Chr. 2a	>	PAPLOG	>	IFITM3P9	XR_654203.1	>	BCL11A	x		
4	Chr. 11	>	MYEOV	<	IFITM9P pseudo	XR_656019.2	>	CCND1	x		
	*Nomascus leucogenys*										
1	Chr. 14	>	PAPLOG	>	IFITM3P9	XR_001114400.1	>	BCL11A	x		
2	Chr. 22a	<	SPDEF	<	IFITM3P3 pseudo	XM_030803316.1	<	ILRUN	x		
5	Chr. 17	>	CHCHD2	>	IFITM3P4 pseudo	XR_004026378.1	>	VOPP1	x		
	*Macaca mulatta*										
1	Chr. 16	<	CTC1	<	3 like 8 (owm P1)	XM_001112566.4	>	RANGRF	in	>	PFAS
2	Chr. 1	>	ATP5PB	>	3 like (owm P2)	XM_001106166.4	>	RAP1A	in	<	TMIGD3
3	Chr. 7	>	MAPK1IP1L	<	3 like (own P3)	XM_001088204.4	>	LGALS3	x		
4	Chr. 10	<	SLC25A17	>	3 ps (owm P4)	XR_001447798.2	>	XPNPEP3	in	<	ST13
	*Cercocebus atys*										
2	Unplaced	>	ATP5PB	>	3 like (owm P2)	XR_001017992.1	>	RAP1A	in	<	TMIGD1
4	Unplaced	<	CTC1	<	3 like 8 (owm P1)	XM_012051598.1	>	RANGRF	in	>	PFAS
6	Unplaced	<	SLC25A17	>	3 ps (owm P4)	XR_001010110.1	>	XPNPEP3	in	<	ST13
7	Unplaced	>	MAPK1IP1L	<	3 like (own P3)	XM_012061884.1	>	LGALS3	x		
10	Unplaced	<	CHRM3	>	1 like (owm P5)	XR_001011033.1	>	ZP4	x		
	*Rhinopithecus roxellana*										
8	Chr. 8	<	CHRM3	>	1 like (owm P5)	XR_750288.2	>	ZP4	x		
	*Aotus nancymaae*										
6	Unplaced	<	AGO2	<	3 ps (nwm P)	XR_002478805.1	<	PTK2	x		
	*Saimiri boliviensis*										
18	Unplaced	<	AGO2	<	3 ps (nwm P)	XM_039464430.1	<	PTK2	x		

in - located in intron.

x - not located in intron.

### 2.8. Additional IFITIMs are IFITM retrogenes

This random distribution and localization in introns of other genes hinted toward transposable element mechanisms and retrogenes. To test this hypothesis, we randomly picked two additional IFITMs from each analyzed species (only one if no more were available) and analyzed the genomic context. For this, we searched for features of retrogenes 200 bp upstream of the canonical start codon and 400 downstream of the canonical stop codon (Supplementary Figure S9). The results are summarized in Table 5.

**Table 5:** Retrogene features of selected primate IFITM retrogenes.

Species	Accession number	Lack of intron	Poly A signal	Poly A tail	Target site duplications (TSDs)	Premature STOP
*Otolemur garnettii*	XR_001161573.1	Yes	Yes	Yes	Yes	No
*Carlito syrichta*	XR_504221.2	Yes	Yes	Yes	Yes	No
	XM_008052862.1	?*	Yes	Yes	Yes	Yes/No*
*Saimiri boliviensis*	XM_039468571.1	Yes	Yes	Yes	Yes	No
	XM_039478903.1	Yes	Yes	Yes	Yes	No
*Aotus nancymaae*	XR_002477520.1	Yes	Yes	Yes	Yes	Yes
	XR_001106643.2	Yes	Yes	Yes	Yes	Yes
*Rhinopithecus roxellana*	XR_748909.2	Yes	Yes	Yes	Yes	No
	XM_030922644.1	Yes	Yes	Yes	Yes	No
*Cercocebus atys*	XR_001017992.1	Yes	Yes	Yes	Yes	No
	XR_001011714.1	Yes	Yes	Yes	Yes	No
*Macaca mulatta*	XM_001112566.4	Yes	Yes	Yes	Yes	No
	XR_001438791.2	Yes	Yes	Yes	Yes	Yes
*Nomascus leucogenys*	XR_004026378.1	Yes	Yes	Yes	Yes	No
	XR_004027821.1	Yes	Yes	Yes	Yes	No
*Pongo abelii*	XR_002913425.1	Yes	Yes	Yes	Yes	No
	XR_656019.2	Yes	Yes	Yes	Yes	No
*Gorilla gorilla*	XR_002005707.2	Yes	Yes	Yes	Yes	No
	XM_004052942.3	Yes	Yes	Yes	Yes	No
*Pan troglodytes*	XR_002913425.1	Yes	Yes	Yes	Yes	No
	XR_169790.4	Yes	Yes	Yes	Yes	Yes
*Pan paniscus*	XM_034961680.1	Yes	Yes	Yes	Yes	Yes
	XM_034966156.1	Yes	Yes	Yes	Yes	Yes
*Homo sapiens*	NG_006210.1	Yes	Yes	Yes	Yes	Yes
	NG_006230.2	Yes	Yes	Yes	Yes	Yes

Shown are the features of retrogenes.

* Not distinguishable if short part of intron or insert.

We observed that all investigated sequences lacked an intron, except for one in *Carlito syrichta*. They had a consensus poly A signal, the start of the poly A tail and target-site duplications (TSDs) adjacent to the poly A tail start and upstream of the canonical start codon. These are all features of retrogenes (Esnault et al., 2000; Kaessmann et al., 2009). For the coding sequences, we also found some with premature stop codons (8/25 tested, e.g., in *Pan paniscus* and *Aotus nancymaae*), which are an indication for retropseudogenes.

Since we observed that the additional *IFITMs* were retrogenes, we compared them with genes from the canonical cluster to infer their origin. For this, we aligned two selected *IFITM* retrogene genomic sequences of each species with the mRNAs of *IR-pIFITM3, IR-pIFITMnwm* or *IR-pIFITMpro* from the canonical cluster (Supplementary Figure S10). We observed that the genomic sequences aligned with the mRNA sequences of *IR-pIFITM3, IR-pIFITMnwm* or *IR-pIFITMpro*, suggesting that these might have been the origin (parental genes) of the IFITMs retrogenes. Further, we observed that the two selected IFITM retrogenes aligned better with the canonical mRNA of *IR-pIFITM3*, *IR-pIFITMnwm* or *IR-pIFITMpro* from the same species and that even the 5′ and 3’ UTR parts aligned with only few nucleotide mismatches (Supplementary Figure S10). This suggests that the emergence from their parental gene was a recent event. In summary, the additional IFITMs are retrogenes or retropseudogenes that exhibit various retrogenic features and could have originated from parental genes in the canonical cluster in a more recent event.

## 3. Discussion

In this study, we examined the evolution of the IFITM protein family in primate species. Our synteny analyses suggest that primate IFITMs can be classified according to their localization within the genome into a canonical IFITM cluster, which includes *IFITM5*, *IFITM10*, *IR-IFITM*, and *IFITM* retrogenes (Figure 2). We observed that the primates *IFITM5* and *IFITM10* were present as single copy genes with conserved synteny: IFITM5 was flanked by PGGHG and IFITM10 by CTSD (Figure 1). This high conservation and the presence of a single copy are most likely related to their essential function as shown by the link between their absence or the presence of mutations and diseases (Hanagata et al., 2011; Liu et al., 2021; Hedjazi et al., 2022). In contrast, a diversification of the gene copy numbers of the IR-IFITMs (zero to six genes) and their synteny occurred in primates after their separation from prosimians around 74 MYA, which consistently possessed two copies of *IR-pIFITMpro* (Figure 1) (Kumar et al., 2022). IR-IFITMs of new and old world monkeys underwent massive rearrangements with gene expansions and losses. In contrast, apes uniformly possessed three IR-IFITM genes, arranged identically; therefore, at least one duplication event must have occurred after the separation from the prosimians. We can only speculate that the synteny is more conserved in apes and prosimians, because they have shared the same specificity for pathogens due to their close relationship. The overall high variability in the number of IR-IFITMs genes in the primate species could be related to their function in the immune response and co-evolution with species-specific pathogens as seen for other immunity-related proteins (Nei et al., 1997; Côrte-Real et al., 2020), resulting in repertoires specific for each species. In line with this, primate IFITMs might follow the birth-and-death model evolution that often occurs in immunity-related genes (Nei et al., 1997; Nei and Rooney, 2005).

In contrast to other phylogenetic studies including primate IFITMs (Siegrist et al., 2011; Hickford et al., 2012; Zhang et al., 2012; Compton et al., 2016; Wilkins et al., 2016; Benfield et al., 2020), we conducted a study including more primate species (26 species) while the others focused on smaller subsets, which improved the resolution of our phylogenetic analysis. Further, we focused our phylogenetic analyses on the IFITMs in the canonical clusters (Figure 2) with the underlying hypothesis that these IR-IFITMs suffered similar selective pressures. In contrast, we assumed that IFITM retrogenes (see below), experienced differences in the selective pressure, probably due to their redundancy, genomic localization, and pseudogenization accompanied by altered expression (Kaessmann et al., 2009; Troskie et al., 2021). The exclusion of these IFITM retrogenes allowed us to reduce bias from the altered selection pressure and improved the alignments, the basis of the phylogeny, by removing indels.

Hickford et al. (2012) focused on marsupial IFITMs and reported only the presence of canonical IFITMs with overall low similarity to other paralogs at the AA level. In line with that, Benfield and colleagues identified chiropteran IFITMs that formed a monophyletic group separated from other taxa by a relatively long branch (Benfield et al., 2020). On the other hand, Zhang et al. (2012) performed a more general evolutionary analysis of mammalian and non-mammalian IFITMs, including only six primate species. They found that all IR-IFITM genes from the different lineages formed their own subgroups, suggesting gene duplication of IR-IFITM as an evolutionary mechanism after species separation. Focusing on the evolution of primate IFITM3s, Compton et al. (2016) identified an atypical gene locus in humans compared to bush baby species and suggested gene gain and loss events for primate evolution. A high number of pseudogenes per IFITM genes was already noted for human paralogs by Siegrist et al. (2011).

Based on our phylogenetic analyses (Figure 3) and further supported by their sequence characteristics, length and AA sequences of the N- and the C-termini (Figure 4), we found six groups of primate IR-IFITMs. Therefore, we propose a new classification: IR-pIFITM1, IR-pIFITM2 and IR-pIFITM3, in line with previous studies (Hickford et al., 2012; Zhang et al., 2012; Compton et al., 2016; Benfield et al., 2020), and three new groups, the IR-pIFITMnwm, IR-pIFITMowm and IR-pIFITMpro (Figure 3). A shortcoming of our study is the lack of functional studies, especially those that have not been studied before such as pIFITMpro. However, our more in-depth evolutionary analyses might guide future functional studies.

The IR-pIFITMpro group is only present in prosimians. It is noteworthy that the two IFITMs genes of the prosimians belong to the IR-pIFITMpro group and neither IR-pIFITM1 nor IR-pIFITM3 are present. It is unclear whether the prosimian ancestor possessed IR-pIFITM1 and/or IR-pIFITM3 “progenitors,” which were lost as a result of concerted evolution with the emergence of an IR-pIFITMpro group, or *vice-versa*: the birth-and-death model of evolution led to the emergence of IR-pIFITM1 and IR-pIFITM3/nwm “progenitor” in the Simiiformes. The subsequent separation of the IR-pIFITM3/nwm “progenitor” into IR-pIFITM3 and IR-pIFITMnwm could have been caused by similar mechanisms. The concerted evolution hypothesis is backed up by our finding of several highly supported subgroups (>83 bootstraps) of IR-IFITM3/nwm from the same species (Figure 3, e.g., *Callithrix jacchus* and *Papio Anubis*) and two chimeras between IR-pIFITM3/nwm and IR-pIFITM1 (Figure 5), suggesting gene conversion in new and old world monkeys and, therefore, a concerted evolution mechanism (Nei and Rooney, 2005). The IR-pIFTM2 genes are most likely a duplication of IR-pIFITM3, which gradually diverged in the apes.

Regarding the IR-pIFITMowm group, each species, except *Colobus angolensis* and *Piliocolobus tephrosceles,* had one *IR-pIFITMowm* gene. The phylogeny suggests that it probably arose by deletions from a duplication of an *IR-pIFITM3* (Figure 3), but we cannot exclude gene conversion or a chimeric origin, as it is not possible to assign an origin based on sequence motifs due to truncations at the C- and N-termini (Figure 4). One copy has been stably maintained in all but two old world monkey species, suggesting an evolutionary advantage for its presence. A possible explanation might be that IR-pIFITMowms were active against a bacterial or a viral pathogen or may have acquired a new function (neofunctionalization) and were thus maintained. Taken together, we found evidence for both concerted evolution and the birth-and-death evolution model for the canonical cluster of the IR-pIFITMs, which could indicate their evolution by a possible mixed process of both models (Nei and Rooney, 2005). The evolution of *IFITM5* and *IFITM10*, which had only one highly conserved copy at canonical positions in each species, were in line with the primate evolution (Supplementary Figures S1, S3; Figure 1).

**Figure 6:**
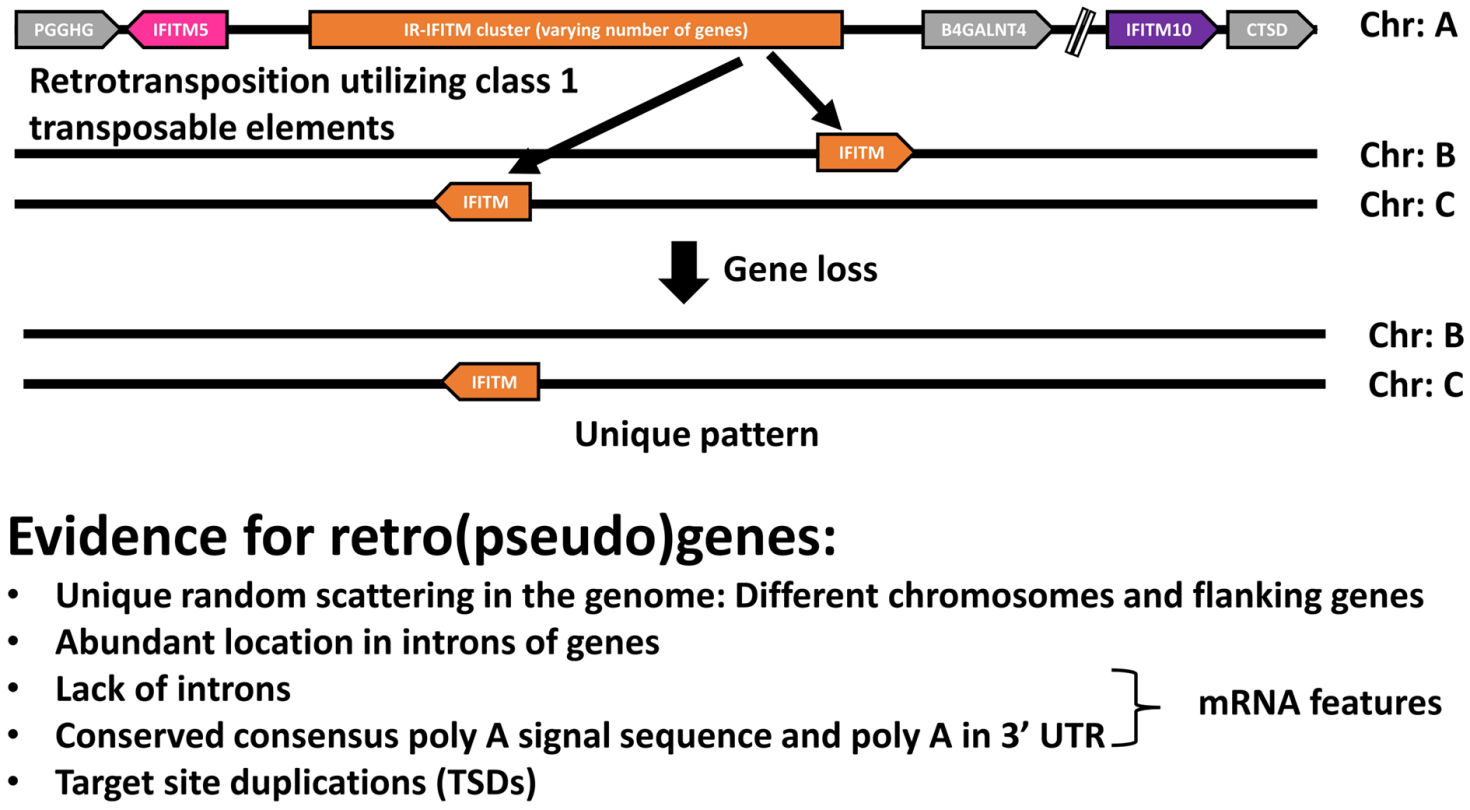
Hypothesis for the origin and pattern of the IFITM retrogenes. Schematic representation of our hypothesis: the transcript of a canonical IR-pIFITM3/nwm/pro is constantly retrotranspositioned by class 1 transposable elements originating the retro(pseudo)genes. The unique pattern of each species is caused by constant pseudogenization and loss of the retro(pseudo)genes. Evidence supporting the hypothesis are listed.

The number of the IFITMs not in the canonical cluster was expanded in Simiiformes, probably after the separation from the prosimians (Table 2). Based on their synteny, we found that they were randomly distributed throughout the genomes and that a fraction of them were located in the intronic regions of other genes (Supplementary Table S2; Table 3). Since some IFITM genes, including human IFITM4P, have been proposed to be retrogenes (Siegrist et al., 2011; Rahman and Compton, 2021), we hypothesized that any additional primate IFITMs might also be retrogenes. Our analyses demonstrated that, along with their randomly scattered location and location within introns, all of them possessed additional features of retrogenes retrotransposed by class 1 transposable elements, such as lack of introns, the presence of conserved poly A signal (AATAAA), poly A start, and target site duplications (TSDs; 5′ and 3′ UTR) (Table 5) (Esnault et al., 2000; Kaessmann et al., 2009) and can therefore be designated as retrogenes. Sixteen of the analyzed genes had a complete coding sequence, but eight presented premature stop codons, which allowed their classification as retrogenes and retropseudogenes, respectively. In the alignment of the IFITM retrogene genomic sequences with the mRNA sequences of the canonical IR-pIFITM3, IR-pIFITMnwm or IR-pIFITMpro, we observed that the genomic sequences aligned best with the mRNA sequences of the IR-pIFITM3, IR-pIFITMnwm or IR-pIFITMpro from the same species, respectively. Furthermore, we observed that even the 5′ and 3′ UTR parts aligned with only few nucleotide mismatches with the mRNA sequences of the IR-pIFITM3, IR-pIFITMnwm or IR-pIFITMpro from the same species (Supplementary Figure S10). This suggests that the transcript of these canonical IFITMs may have been the origin (parental gene) of the retro(pseudo)gene, and that the event was recent because the TSDs and the poly A signal and tail, which degenerate over time, were mostly intact (Kaessmann et al., 2009). In conclusion, we hypothesize that the transcript of a canonical IR-pIFITM3/nwm/pro has been constantly retrotranspositioned by class 1 transposable elements, building the retro(pseudo)genes. The unique species-specific pattern was caused by constant pseudogenization and/or loss of the IFITM retro(pseudo)genes (Figure 6). The reason for the preferential integration of IR-pIFITM3/nwm/pro transcripts remains unclear but enrichment of retro(pseudo)gene mRNAs was observed in LINE-1 ribonucleoproteins (mediating retrotransposition) (Mandal et al., 2013). We hypothesize that the high abundance of their mRNAs in the germline might have favored their binding and retrotransposition (Zhang et al., 2003, 2004). This might be caused either by interferon induction (Friedman et al., 1984) as an innate immunity response to specific pathogens or their general expression in germline cells, which has been shown for mouse ifitms (Tanaka and Matsui, 2002). However, an unknown mechanisms could have also played a role since LINE-1 RNA is preferentially retrotranspositioned compared to other mRNAs (Esnault et al., 2000; Kulpa and Moran, 2006). It is also possible that other mRNA properties play a role similar to the poly A tail requirement for retrotransposition (Doucet et al., 2015). The maintenance of a high number of such retro(pseudo)genes in higher primate species is also unclear. Indeed, in some cases, it could have compensated or caused the loss of the canonical IFITMs (e.g., *Piliocolobus tephrosceles*). In other cases, it might represent an additional selective advantage by their expression in response to a viral infection. This was recently shown for human IFITM4P, a retropseudogene, which is not coding for a protein (Xiao et al., 2021). However, the rate of retrotransposition and therefore the emergence of retro(pseudo)genes could be simply exceeding the rate at which pseudogenization and gene loss occur in higher primates.

In conclusion, we found evidence for concerted evolution and birth-and-death evolution model for the canonical cluster IR-pIFITMs. For the IFITM retro(pseudo)genes, we propose a new hypothesis for their origin and pattern (Figure 6) through a third mechanism of evolution, similar to the birth-and-death model of evolution, but via a transposable element mechanism leading to IFITM retro(pseudo)genes. Primate IFITMs were thus the result of a mixed evolutionary process combining three different mechanisms.

## 4. Materials and methods

### 4.1. Gene synteny analysis

Primate IFITM sequences were retrieved from https://www.ncbi.nlm.nih.gov/; BLASTn analysis ensured that all available sequences per species were included. Accession numbers of all retrieved sequences are found in Supplementary Table S1. The NCBI Genomic Data Viewer^1^ was used to determine the genomic localization and orientation of the IFITMs in the 26 analyzed primate species. The primate phylogeny was obtained using Timetree.org (Kumar et al., 2022).

### 4.2. Sequence alignments

Sequences were initially aligned using MEGA11 (Tamura et al., 2021) and MUSCLE algorithm (Edgar, 2004). Alignments were then visually inspected and manually corrected in BioEdit (Hall, 1999).

### 4.3. Phylogenetic analysis

For AA sequences, the evolutionary history was inferred using the Maximum Likelihood (ML) method. The percentage of trees in which the associated taxa clustered together is shown next to the branches and was obtained by conducting 1,000 bootstrap replicates. Initial tree(s) for the heuristic search were obtained automatically by applying Neighbor-Joining and BioNJ algorithms to a matrix of pairwise distances estimated using the JTT model (Jones et al., 1992), and then selecting the topology with superior log likelihood value. A discrete Gamma distribution was used to model evolutionary rate differences among sites [5 categories (+G)]. The trees were drawn to scale, with branch lengths measured in the number of substitutions per site. All positions with less than 95% site coverage were eliminated, i.e., fewer than 5% alignment gaps, missing data, and ambiguous bases were allowed at any position (partial deletion option). Analyses were conducted in MEGA11 (Tamura et al., 2021).

### 4.4. Sequence logos

For the generation of the sequence logos, WebLogo 3^2^ was used (Crooks et al., 2004). Alignments (Supplementary Figures S6–S8) were used as input.

### 4.5. Transposable element features analysis

We considered random unique localization, localization in introns of other genes, lack of introns, conserved poly A signal (AATAAA), poly A tail start, target-site duplications (5′ and 3′ UTR) (Kaessmann et al., 2009), and full coding sequences as features for retro(pseudo)genes. Localization (random, unique, in introns) was obtained from our synteny data. For the other features, we analyzed the genomic sequence of the IFITMs 200 bp upstream of the canonical start codon and 400 bp downstream of the canonical stop codon. Lack of introns was obtained from the annotations found at NCBI and genomic sequence. Sequences were manually inspected for canonical start codon, canonical stop codon, premature stop codon, poly A signal (AATAAA), poly A start and TSDs.

## Data availability statement

The original contributions presented in the study are included in the article/Supplementary material, further inquiries can be directed to the corresponding authors.

## Author contributions

LS: conceptualization, data curation, formal analysis, writing—original draft, writing—review and editing. JA: funding acquisition, writing—review and editing. H-MB: supervision, writing—review and editing, funding acquisition. PE: conceptualization, writing—review and editing, funding acquisition. All authors contributed to the article and approved the submitted version.

## Funding

This work was supported by Fundação para a Ciência e Tecnologia (FCT) - Portugal, supported the Assistant Researcher grant of JA (CEECIND/00078/2017) and the Principal Researcher grant of PE (CEECIND/01495/2020). This work was co-funded by the project NORTE-01-0246-FEDER-000063, supported by Norte Portugal Regional Operational Programme (NORTE2020), under the PORTUGAL 2020 Partnership Agreement, through the European Regional Development Fund (ERDF). H-MB acknowledges funding from the Deutsche Forschungsgemeinschaft (DFG) (BA-6820/1-1). H-MB and JA acknowledge the project-related personal exchange (PPP) program of the FCT/German Academic Exchange Service (DAAD) (57518622). This work was supported by the young Society for Virology (jGfV) lab rotation scholarship granted to LS.

## Conflict of interest

The authors declare that the research was conducted in the absence of any commercial or financial relationships that could be construed as a potential conflict of interest.

## Publisher’s note

All claims expressed in this article are solely those of the authors and do not necessarily represent those of their affiliated organizations, or those of the publisher, the editors and the reviewers. Any product that may be evaluated in this article, or claim that may be made by its manufacturer, is not guaranteed or endorsed by the publisher.

## Footnotes

^1^https://www.ncbi.nlm.nih.gov/genome/gdv/

^2^https://weblogo.threeplusone.com/
