# Supplementary material for: Evolution of primate interferon-induced transmembrane proteins (IFITMs): a story of gain and loss with a differentiation into a canonical cluster and IFITM retrogenes
Source: Front Microbiol. 2023 Jul 26;14:1213685. doi: 10.3389/fmicb.2023.1213685 (PMC10415907; doi:10.3389/fmicb.2023.1213685)
Supplement: Supplementary file 1 [file Data_Sheet_1.pdf]

frontiers

Supplementary Material

Evolution of primate interferon-induced transmembrane proteins (IFITMs): a story of gain and loss with a differentiation into canonical cluster and IFITM retrogenes

Luca Schelle[1], Joana Abrantes[2,3,4], Hanna-Mari Baldauf[1+*], Pedro José Esteves[2,3,4,5+*]

[1]Max von Pettenkofer Institute and Gene Center, Virology, National Reference Center for Retroviruses, Faculty of Medicine, LMU München, Munich, Germany

[2]CIBIO-InBIO, Research Center in Biodiversity and Genetic Resources, University of Porto, 4485-661 Vairão, Portugal

[3]BIOPOLIS Program in Genomics, Biodiversity and Land Planning, CIBIO, Campus de Vairão, 4485-661, Vairão, Portugal

[4]Departamento de Biologia, Faculdade de Ciências, Universidade do Porto, 4099-002 Porto, Portugal

[5]CITS - Center of Investigation in Health Technologies, CESPU, 4585-116 Gandra, Portugal

*** Correspondence:**
Hanna-Mari Baldauf
baldauf@mvp.lmu.de
Pedro José Esteves
pjesteves@cibio.up.pt

+ These authors share last authorship

Supplementary Tables

Supplement: Accession numbers of used and excluded sequences

Table S1: Accession numbers of used and excluded sequences

	IFITM5 Alignment
1	NM_001025295.3:37-435 Homo sapiens interferon induced transmembrane protein 5 (IFITM5) mRNA
2	XM_003806010.1:31-429 PREDICTED: Pan paniscus interferon induced transmembrane protein 5 (IFITM5) mRNA
3	NM_001198769.1:37-435 Pan troglodytes interferon induced transmembrane protein 5 (IFITM5) mRNA
4	XM_031006316.1:35-433 PREDICTED: Gorilla gorilla gorilla interferon induced transmembrane protein 5 (IFITM5) mRNA
5	XM_024241354.1:31-429 PREDICTED: Pongo abelii interferon induced transmembrane protein 5 (IFITM5) mRNA

6	XM_003281296.3:31-429 PREDICTED: Nomascus leucogenys interferon induced transmembrane protein 5 (IFITM5) mRNA
7	XM_008053676.1:30-434 PREDICTED: Carlito syrichta interferon induced transmembrane protein 5 (IFITM5) mRNA
8	XM_012456046.1:30-428 PREDICTED: Aotus nancymaae interferon induced transmembrane protein 5 (IFITM5) mRNA
9	XM_003943326.1:29-427 PREDICTED: Saimiri boliviensis boliviensis interferon induced transmembrane protein 5 (IFITM5) mRNA
10	XM_002755705.3:27-425 PREDICTED: Callithrix jacchus interferon induced transmembrane protein 5 (IFITM5) mRNA
11	XM_012040929.1:117-515 PREDICTED: Cercocebus atys interferon induced transmembrane protein 5 (IFITM5) mRNA
12	XM_001085215.4:109-507 PREDICTED: Macaca mulatta interferon induced transmembrane protein 5 (IFITM5) mRNA
13	XM_003909306.4:415-813 PREDICTED: Papio anubis interferon induced transmembrane protein 5 (IFITM5) mRNA
14	XM_008001923.2:31-429 PREDICTED: Chlorocebus sabaeus interferon induced transmembrane protein 5 (IFITM5) mRNA
15	XM_025358134.1:31-429 PREDICTED: Theropithecus gelada interferon induced transmembrane protein 5 (IFITM5) mRNA
16	XM_011929981.1:31-429 PREDICTED: Colobus angolensis palliatus interferon induced transmembrane protein 5 (IFITM5) mRNA
17	XM_023183578.2:237-635 PREDICTED: Piliocolobus tephrosceles interferon induced transmembrane protein 5 (IFITM5) mRNA
18	XM_005576715.3:37-435 PREDICTED: Macaca fascicularis interferon induced transmembrane protein 5 (IFITM5) mRNA
19	XM_011762252.1:62-460 PREDICTED: Macaca nemestrina interferon induced transmembrane protein 5 (IFITM5) mRNA
20	XM_017885791.1:31-429 PREDICTED: Rhinopithecus bieti interferon induced transmembrane protein 5 (IFITM5) mRNA
21	XM_010354927.1:31-429 PREDICTED: Rhinopithecus roxellana interferon induced transmembrane protein 5 (IFITM5) mRNA
22	XM_017521000.2:30-428 PREDICTED: Cebus imitator interferon induced transmembrane protein 5 (IFITM5) mRNA
23	XM_012769614.1:17-421 PREDICTED: Microcebus murinus interferon induced transmembrane protein 5 (IFITM5) mRNA
24	XM_012651962.1:58-462 PREDICTED: Propithecus coquereli interferon induced transmembrane protein 5 (IFITM5) mRNA
25	XM_003802761.3:94-498 PREDICTED: Otolemur garnettii interferon induced transmembrane protein 5 (IFITM5) mRNA
26	XM_045558584.1:189-593 PREDICTED: Lemur catta interferon induced transmembrane protein 5 (IFITM5) mRNA

	IFITM10 Alignment
1	NM_001170820.4:154-840 Homo sapiens interferon induced transmembrane protein 10 (IFITM10) mRNA
2	XM_034931928.1:440-832 PREDICTED: Pan paniscus interferon induced transmembrane protein 10 (IFITM10) transcript variant X1 mRNA
3	XM_016920116.2:571-963 PREDICTED: Pan troglodytes interferon induced transmembrane protein 10 (IFITM10) transcript variant X1 mRNA
4	XM_019036679.1:168-854 PREDICTED: Gorilla gorilla gorilla interferon induced transmembrane protein 10 (IFITM10) mRNA
5	XM_024255950.1:1-891 PREDICTED: Pongo abelii interferon induced transmembrane protein 10 (IFITM10) mRNA
6	XM_030811506.1:662-1054 PREDICTED: Nomascus leucogenys interferon induced transmembrane protein 10 (IFITM10) transcript variant X2 mRNA
7	XM_012456043.1:1-714 PREDICTED: Aotus nancymaae interferon induced transmembrane protein 10 (IFITM10) mRNA
8	XM_039470357.1:1-918 PREDICTED: Saimiri boliviensis boliviensis interferon induced transmembrane protein 10 (IFITM10) mRNA
9	XM_012041114.1:326-718 PREDICTED: Cercocebus atys interferon induced transmembrane protein 10 (IFITM10) transcript variant X1 mRNA
10	XM_028833028.1:616-1008 PREDICTED: Macaca mulatta interferon induced transmembrane protein 10 (IFITM10) transcript variant X3 mRNA
11	XM_031653828.1:649-1041 PREDICTED: Papio anubis interferon induced transmembrane protein 10 (IFITM10) transcript variant X1 mRNA

12	XM_025355874.1:139-849 PREDICTED: Theropithecus gelada interferon induced transmembrane protein 10 (IFITM10) transcript variant X1 mRNA
13	XM_011930050.1:10-840 PREDICTED: Colobus angolensis palliatus interferon induced transmembrane protein 10 (IFITM10) transcript variant X1 mRNA
14	XM_023183427.1:546-938 PREDICTED: Piliocolobus tephrosceles interferon induced transmembrane protein 10 (IFITM10) mRNA
15	XM_045372531.1:1-1095 PREDICTED: Macaca fascicularis interferon induced transmembrane protein 10 (IFITM10) mRNA
16	XM_011721336.2:411-803 PREDICTED: Macaca nemestrina interferon induced transmembrane protein 10 (IFITM10) transcript variant X1 mRNA
17	XM_017874696.1:655-1047 PREDICTED: Rhinopithecus bieti interferon induced transmembrane protein 10 (IFITM10) transcript variant X2 mRNA
18	XM_010382024.2:442-834 PREDICTED: Rhinopithecus roxellana interferon induced transmembrane protein 10 (IFITM10) mRNA
19	XM_020286416.1:27-851 PREDICTED: Microcebus murinus interferon induced transmembrane protein 10 (IFITM10) transcript variant X1 mRNA
20	XM_012651941.1 PREDICTED: Propithecus coquereli interferon induced transmembrane protein 10 (IFITM10) mRNA
21	XM_012810449.2:1-996 PREDICTED: Otolemur garnettii interferon induced transmembrane protein 10 (IFITM10) mRNA
22	XM_045558430.1:389-781 PREDICTED: Lemur catta interferon induced transmembrane protein 10 (IFITM10) transcript variant X1 mRNA

	Excluded IFITM10
1	XM_035263794.1:651-1049 PREDICTED: Callithrix jacchus interferon induced transmembrane protein 10 (IFITM10) transcript variant X1 mRNA
2	XM_037997344.1:1-762 PREDICTED: Chlorocebus sabaeus interferon induced transmembrane protein 10 (IFITM10) mRNA
3	XM_017520417.2:1-1017 PREDICTED: Cebus imitator interferon induced transmembrane protein 10 (IFITM10) mRNA

	IFITM123 Alignment
1	XM_012040930.1:109-510 PREDICTED: Cercocebus atys interferon-induced transmembrane protein 3-like (LOC105577185) mRNA
2	XM_028832948.1:88-489 PREDICTED: Macaca mulatta interferon-induced transmembrane protein 3-like (LOC114672189) mRNA
3	XM_015113207.2:87-488 PREDICTED: Macaca mulatta interferon-induced transmembrane protein 3 (LOC697829) mRNA
4	XM_015113206.2:109-510 PREDICTED: Macaca mulatta interferon-induced transmembrane protein 3 (LOC697564) mRNA
5	XM_031662204.1:109-510 PREDICTED: Papio anubis interferon-induced transmembrane protein 3-like (LOC116273247) transcript variant X1 mRNA
6	XM_031653856.1:109-510 PREDICTED: Papio anubis interferon-induced transmembrane protein 3-like (LOC100999022) transcript variant X1 mRNA
7	XM_005576716.3:36-437 PREDICTED: Macaca fascicularis interferon-induced transmembrane protein 3-like (LOC102145938) transcript variant X1 mRNA
8	XM_005576719.3:85-486 PREDICTED: Macaca fascicularis interferon-induced transmembrane protein 3 (LOC102146935) transcript variant X1 mRNA
9	XM_011762251.1:109-510 PREDICTED: Macaca nemestrina interferon-induced transmembrane protein 3-like (LOC105494124) transcript variant X1 mRNA
10	XM_011762255.2:109-510 PREDICTED: Macaca nemestrina interferon-induced transmembrane protein 3-like (LOC105494127) transcript variant X1 mRNA
11	XM_010354836.2:86-487 PREDICTED: Rhinopithecus roxellana interferon-induced transmembrane protein 3 (LOC104655384) mRNA
12	XM_017885790.1:109-510 PREDICTED: Rhinopithecus bieti interferon-induced transmembrane protein 3 (LOC108537995) mRNA
13	XM_010354744.2:2-520 PREDICTED: Rhinopithecus roxellana interferon-induced transmembrane protein 3-like (LOC104655295) mRNA
14	XM_017521001.2:83-484 PREDICTED: Cebus imitator interferon-induced transmembrane protein 3 (LOC108298304) mRNA
15	NM_006435.3:378-776 Homo sapiens interferon induced transmembrane protein 2 (IFITM2) mRNA
16	XM_034931794.1:85-483 PREDICTED: Pan paniscus interferon induced transmembrane protein 2 (IFITM2) mRNA

17	NM_001198767.1:280-678 Pan troglodytes interferon induced transmembrane protein 2 (IFITM2) mRNA
18	XM_009245970.2:117-515 PREDICTED: Pongo abelii interferon-induced transmembrane protein 3-like (LOC100455435) mRNA
19	XM_004050342.2:98-496 PREDICTED: Gorilla gorilla gorilla interferon induced transmembrane protein 2 (IFITM2) mRNA
20	NM_021034.3:48-449 Homo sapiens interferon induced transmembrane protein 3 (IFITM3) transcript variant 1 mRNA
21	XM_034951329.1:233-634 PREDICTED: Pan paniscus interferon-induced transmembrane protein 3 (LOC100971164) mRNA
22	NM_001198757.1:36-437 Pan troglodytes interferon induced transmembrane protein 3 (IFITM3) mRNA
23	XM_004050337.3:99-500 PREDICTED: Gorilla gorilla gorilla interferon-induced transmembrane protein 3 (LOC101128980) transcript variant X2 mRNA
24	XM_002821311.4:236-637 PREDICTED: Pongo abelii interferon-induced transmembrane protein 3 (LOC100453977) mRNA
25	XM_003281297.4:236-637 PREDICTED: Nomascus leucogenys interferon-induced transmembrane protein 3 (LOC100594097) mRNA
26	XM_030801594.1:107-508 PREDICTED: Nomascus leucogenys interferon-induced transmembrane protein 3 (LOC101175734) mRNA
27	XM_012456047.2:193-594 PREDICTED: Aotus nancymaae interferon-induced transmembrane protein 3 (LOC105719828) mRNA
28	XM_039471018.1:97-498 PREDICTED: Saimiri boliviensis boliviensis interferon-induced transmembrane protein 3-like (LOC120364636) mRNA
29	XM_003943324.3:85-486 PREDICTED: Saimiri boliviensis boliviensis interferon-induced transmembrane protein 3 (LOC101048198) mRNA
30	XM_039471016.1:84-485 PREDICTED: Saimiri boliviensis boliviensis interferon-induced transmembrane protein 3-like (LOC120364635) mRNA
31	XM_035263965.1:196-597 PREDICTED: Callithrix jacchus interferon-induced transmembrane protein 3-like (LOC118145581) mRNA
32	XM_035263964.1:296-697 PREDICTED: Callithrix jacchus interferon-induced transmembrane protein 3-like (LOC118145580) mRNA
33	NM_003641.5:132-509 Homo sapiens interferon induced transmembrane protein 1 (IFITM1) mRNA
34	XM_034931795.1:141-518 PREDICTED: Pan paniscus interferon induced transmembrane protein 1 (IFITM1) mRNA
35	NM_001198758.1:132-509 Pan troglodytes interferon induced transmembrane protein 1 (IFITM1) mRNA
36	XM_004050339.2:335-712 PREDICTED: Gorilla gorilla gorilla interferon induced transmembrane protein 1 (IFITM1) mRNA
37	NM_001198762.1:137-514 Pongo abelii interferon induced transmembrane protein 1 (IFITM1) mRNA
38	XM_030801595.1:318-695 PREDICTED: Nomascus leucogenys interferon induced transmembrane protein 1 (IFITM1) mRNA
39	XM_035263963.1:191-571 PREDICTED: Callithrix jacchus interferon induced transmembrane protein 1 (IFITM1) mRNA
40	XM_012040931.1:163-540 PREDICTED: Cercocebus atys interferon induced transmembrane protein 1 (IFITM1) mRNA
41	XM_028832947.1:159-671 PREDICTED: Macaca mulatta interferon-induced transmembrane protein 1 (LOC697687) transcript variant X1 mRNA
42	XM_021677005.1:371-763 PREDICTED: Aotus nancymaae interferon-induced transmembrane protein 1 (LOC105719843) transcript variant X1 mRNA
43	XM_011929983.1:320-697 PREDICTED: Colobus angolensis palliatus interferon induced transmembrane protein 1 (IFITM1) transcript variant X2 mRNA
44	XM_009185182.4:189-566 PREDICTED: Papio anubis interferon-induced transmembrane protein 1 (LOC101000083) transcript variant X1 mRNA
45	XM_005576718.3:119-496 PREDICTED: Macaca fascicularis interferon induced transmembrane protein 1 (IFITM1) mRNA
46	XM_011762253.1:163-540 PREDICTED: Macaca nemestrina interferon induced transmembrane protein 1 (IFITM1) mRNA
47	XM_017885792.1:176-553 PREDICTED: Rhinopithecus bieti interferon induced transmembrane protein 1 (IFITM1) mRNA
48	XM_010354645.2:311-688 PREDICTED: Rhinopithecus roxellana interferon induced transmembrane protein 1 (IFITM1) mRNA
49	XM_003943325.3:210-590 PREDICTED: Saimiri boliviensis boliviensis interferon-induced transmembrane protein 1 (LOC101048512) mRNA

50	XM_001116556.3:1055-1345 PREDICTED: Macaca mulatta interferon-induced transmembrane protein 3-like (LOC720763) mRNA
51	XM_008004069.2:16-306 PREDICTED: Chlorocebus sabaeus interferon-induced transmembrane protein 3 (LOC103238550) transcript variant X1 mRNA
52	XM_011762371.2:1-627 PREDICTED: Macaca nemestrina interferon-induced transmembrane protein 2-like (LOC105494172) mRNA
53	XM_025358469.1 PREDICTED: Theropithecus gelada interferon-induced transmembrane protein 3-like (LOC112607347) mRNA
54	XM_005576720.3:788-1078 PREDICTED: Macaca fascicularis interferon-induced transmembrane protein 3-like (LOC102115008) mRNA
55	XM_017894043.1:1-291 PREDICTED: Rhinopithecus bieti interferon-induced transmembrane protein 3-like (LOC108543531) mRNA
56	XM_010356490.2:27-317 PREDICTED: Rhinopithecus roxellana interferon-induced transmembrane protein 3-like (LOC104656765) mRNA
57	XM_009185183.3:1-324 PREDICTED: Papio anubis interferon-induced transmembrane protein 2-like (LOC101000441), mRNA
58	XM_012652051.1:102-503 PREDICTED: Propithecus coquereli interferon-induced transmembrane protein 3-like (LOC105816571) mRNA
59	XM_012651945.1:103-504 PREDICTED: Propithecus coquereli interferon-induced transmembrane protein 3-like (LOC105816515) mRNA
60	XM_003802780.3:72-464 PREDICTED: Otolemur garnettii interferon-induced transmembrane protein 3-like (LOC100943108) mRNA
61	XM_003802781.3:34-414 PREDICTED: Otolemur garnettii interferon-induced transmembrane protein 3-like (LOC100943419) mRNA
62	XM_045558583.1:21-425 PREDICTED: Lemur catta interferon-induced transmembrane protein 3-like (LOC123642983) mRNA
63	XM_045558582.1:50-625 PREDICTED: Lemur catta interferon-induced transmembrane protein 3-like (LOC123642982) mRNA
64	XM_012769579.2:117-515 PREDICTED: Microcebus murinus interferon-induced transmembrane protein 3-like (LOC105874071), mRNA
65	XM_012769578.2:92-493 PREDICTED: Microcebus murinus interferon-induced transmembrane protein 3-like (LOC105874070) mRNA
66	NM_030694.1:47-481 Mus musculus interferon induced transmembrane protein 2 (Ifitm2), mRNA
67	NM_026820.3:155-475 Mus musculus interferon induced transmembrane protein 1 (Ifitm1), transcript variant 1, mRNA
68	NM_025378.2:100-513 Mus musculus interferon induced transmembrane protein 3 (Ifitm3), mRNA

	Excluded IFITM123
1	XM_012042177.1:1-528 PREDICTED: Cercocebus atys interferon-induced transmembrane protein 1-like (LOC105577604), mRNA
2	XM_039471099.1:77-718 PREDICTED: Saimiri boliviensis boliviensis interferon-induced transmembrane protein 3-like (LOC120364642), transcript variant X1, mRNA
3	XM 025357802.1:108-701 PREDICTED: Theropithecus gelada interferon-induced transmembrane protein 1 (LOC112606872) transcript variant X1 mRNA
4	XM_008053679.1 PREDICTED: Carlito syrichta interferon-induced transmembrane protein 3-like (LOC103255722), partial mRNA
5	PREDICTED: Saimiri boliviensis boliviensis interferon-induced transmembrane protein 3-like (LOC120364575), partial mRNA

	Chimeras Alignment
1	XM_039471099.1:77-718 PREDICTED: Saimiri boliviensis boliviensis interferon-induced transmembrane protein 3-like (LOC120364642), transcript variant X1, mRNA
2	XM_039471016.1:84-485 PREDICTED: Saimiri boliviensis boliviensis interferon-induced transmembrane protein 3-like (LOC120364635) mRNA
3	XM_003943325.3:210-590 PREDICTED: Saimiri boliviensis boliviensis interferon-induced transmembrane protein 1 (LOC101048512) mRNA
4	XM_025357802.1:108-701 PREDICTED: Theropithecus gelada interferon-induced transmembrane protein 1 (LOC112606872) transcript variant X1 mRNA
5	XM_031662204.1:109-510 PREDICTED: Papio anubis interferon-induced transmembrane protein 3-like (LOC116273247) transcript variant X1 mRNA
6	XM_009185182.4:189-566 PREDICTED: Papio anubis interferon-induced transmembrane protein 1 (LOC101000083) transcript variant X1 mRNA

	Additional IR-IFITMs
1	NR_001590.1 Homo sapiens interferon induced transmembrane protein 4 pseudogene (IFITM4P), non-coding RNA
2	NG_006225.2 Homo sapiens IFITM3 pseudogene 5 (IFITM3P5) on chromosome 12
3	NG_006230.2 Homo sapiens IFITM3P pseudogene 6 (IFITM3P6) on chromosome 12
4	NG_006210.1 Homo sapiens interferon induced transmembrane protein 9 pseudogene (IFITM9P) on chromosome 11
5	NG_006229.1 Homo sapiens IFITM3 pseudogene 3 (IFITM3P3) on chromosome 6
6	NG_006223.3 Homo sapiens IFITM3 pseudogene 4 (IFITM3P4) on chromosome 7
7	NG_006204.1 Homo sapiens IFITM3 pseudogene 1 (IFITM3P1) on chromosome 4
8	NG_006205.3 Homo sapiens IFITM3 pseudogene 2 (IFITM3P2) on chromosome 12
9	NG_006227.1 Homo sapiens IFITM3 pseudogene 7 (IFITM3P7) on chromosome 1
10	NG_006224.1 Homo sapiens IFITM3 pseudogene 8 (IFITM3P8) on chromosome 8
11	NG_006228.3 Homo sapiens IFITM3 pseudogene 9 (IFITM3P9) on chromosome 2
12	NG_005307.4 Homo sapiens interferon induced transmembrane protein 8 pseudogene (IFITM8P) on chromosome 8
13	XM_034934516.1 PREDICTED: Pan paniscus interferon-induced transmembrane protein 3-like (LOC103783988), mRNA
14	XM_034966156.1 PREDICTED: Pan paniscus interferon-induced transmembrane protein 3-like (LOC100985648), mRNA
15	XM_003813732.5 PREDICTED: Pan paniscus putative dispanin subfamily A member 2d (LOC100976555), mRNA
16	XM_034961680.1 PREDICTED: Pan paniscus interferon-induced transmembrane protein 3-like (LOC100993091), mRNA
17	XM_034953202.1 PREDICTED: Pan paniscus interferon-induced transmembrane protein 3-like (LOC100995269), transcript variant X1, mRNA
18	XM_003952225.4 PREDICTED: Pan troglodytes putative dispanin subfamily A member 2d (LOC465271), mRNA
19	XR_001716631.2 PREDICTED: Pan troglodytes interferon-induced transmembrane protein 3 pseudogene (LOC101057405), misc_RNA
20	XR_169790.4 PREDICTED: Pan troglodytes interferon-induced transmembrane protein 3 pseudogene (LOC101057658), misc_RNA
21	XR_002944366.1 PREDICTED: Pan troglodytes interferon-induced transmembrane protein 3 pseudogene (LOC112209536), misc_RNA
22	XR_001715794.1 PREDICTED: Pan troglodytes interferon-induced transmembrane protein 3 pseudogene (LOC459257), transcript variant X1, misc_RNA
23	XR_002005707.2 PREDICTED: Gorilla gorilla gorilla interferon-induced transmembrane protein 3 pseudogene (LOC109026657), misc_RNA
24	XM_004052942.3 PREDICTED: Gorilla gorilla gorilla putative dispanin subfamily A member 2d (LOC101143671), mRNA
25	XR_002004539.2 PREDICTED: Gorilla gorilla gorilla interferon-induced transmembrane protein 3 pseudogene (LOC101150658), transcript variant X1, misc_RNA
26	XR_656019.2 PREDICTED: Pongo abelii interferon-induced transmembrane protein 3 pseudogene (LOC100443671), misc_RNA
27	XR_002913425.1 PREDICTED: Pongo abelii interferon-induced transmembrane protein 3 pseudogene (LOC100453335), misc_RNA
28	XR_656249.2 PREDICTED: Pongo abelii putative dispanin subfamily A member 2d (LOC100454104), misc_RNA
29	XR_654203.1 PREDICTED: Pongo abelii interferon-induced transmembrane protein 3 pseudogene (LOC100438205), misc_RNA
30	XR_004027821.1 PREDICTED: Nomascus leucogenys interferon-induced transmembrane protein 3 pseudogene (LOC100585336), misc_RNA
31	XR_004026378.1 PREDICTED: Nomascus leucogenys interferon-induced transmembrane protein 3 pseudogene (LOC115830870), misc_RNA
32	XM_030803316.1 PREDICTED: Nomascus leucogenys inflammation and lipid regulator with UBA-like and NBR1-like domains (ILRUN), transcript variant X2, mRNA

33	XR_001114400.1 PREDICTED: Nomascus leucogenys interferon-induced transmembrane protein 3 pseudogene (LOC100600683), misc_RNA
34	XM_030812847.1 PREDICTED: Nomascus leucogenys interferon-induced transmembrane protein 1 (LOC100590408), mRNA
35	XR_001014764.1 PREDICTED: Cercocebus atys interferon-induced transmembrane protein 3 pseudogene (LOC105585156), misc_RNA
36	XR_001017992.1 PREDICTED: Cercocebus atys interferon-induced transmembrane protein 3 pseudogene (LOC105594389), misc_RNA
37	XR_001010903.1 PREDICTED: Cercocebus atys interferon-induced transmembrane protein 3 pseudogene (LOC105574575), misc_RNA
38	XM_012051598.1 PREDICTED: Cercocebus atys interferon-induced transmembrane protein 3 (LOC105581870), mRNA
39	XR_001011714.1 PREDICTED: Cercocebus atys interferon-induced transmembrane protein 3 pseudogene (LOC105576564), misc_RNA
40	XR_001010110.1 PREDICTED: Cercocebus atys interferon-induced transmembrane protein 3 pseudogene (LOC105572549), misc_RNA
41	XM_012061884.1 PREDICTED: Cercocebus atys interferon-induced transmembrane protein 3-like (LOC105586648), mRNA
42	XR_001010401.1 PREDICTED: Cercocebus atys interferon-induced transmembrane protein 3 pseudogene (LOC105573186), misc_RNA
43	XR_001011610.1 PREDICTED: Cercocebus atys interferon-induced transmembrane protein 3 pseudogene (LOC105576426), misc_RNA
44	XR_001011033.1 PREDICTED: Cercocebus atys interferon-induced transmembrane protein 1 pseudogene (LOC105574913), misc_RNA
45	XM_001112566.4 PREDICTED: Macaca mulatta interferon-induced transmembrane protein 3 (LOC717105), mRNA
46	XM_001106166.4 PREDICTED: Macaca mulatta interferon-induced transmembrane protein 3 (LOC704623), mRNA
47	XR_003722396.1 PREDICTED: Macaca mulatta interferon-induced transmembrane protein 3-like (LOC114672188), misc_RNA
48	XM_001088204.4 PREDICTED: Macaca mulatta interferon-induced transmembrane protein 3 (LOC696527), mRNA
49	XR_001447798.2 PREDICTED: Macaca mulatta interferon-induced transmembrane protein 3 pseudogene (LOC100430243), misc_RNA
50	XR_001438791.2 PREDICTED: Macaca mulatta interferon-induced transmembrane protein 3 pseudogene (LOC706375), misc_RNA
51	XR_001447146.2 PREDICTED: Macaca mulatta interferon-induced transmembrane protein 3 pseudogene (LOC107000162), misc_RNA
52	XR_001448216.2 PREDICTED: Macaca mulatta interferon-induced transmembrane protein 3 pseudogene (LOC107001016), misc_RNA
53	XM_001113914.2 PREDICTED: Macaca mulatta interferon-induced transmembrane protein 3 (LOC719537), mRNA
54	XM_001085444.4 PREDICTED: Macaca mulatta interferon-induced transmembrane protein 1 (LOC697687), transcript variant X2, mRNA
55	XR_003722397.1 PREDICTED: Macaca mulatta interferon-induced transmembrane protein 1-like (LOC114672190), misc_RNA
56	XM_028832920.1 PREDICTED: Macaca mulatta uncharacterized LOC114672158 (LOC114672158), partial mRNA
57	XM_045385282.1 PREDICTED: Macaca fascicularis interferon-induced transmembrane protein 3-like (LOC102145452), mRNA
58	XM_045375359.1 PREDICTED: Macaca fascicularis interferon-induced transmembrane protein 3-like (LOC102122364), mRNA
59	XM_015453812.2 PREDICTED: Macaca fascicularis interferon-induced transmembrane protein 3 (LOC102144965), mRNA
60	XR_006691311.1 PREDICTED: Macaca fascicularis interferon-induced transmembrane protein 3-like (LOC102138757), misc_RNA
61	XM_045363567.1 PREDICTED: Macaca fascicularis interferon-induced transmembrane protein 3-like (LOC102132866), mRNA
62	XR_006697307.1 PREDICTED: Macaca fascicularis interferon-induced transmembrane protein 3 (LOC102128635), misc_RNA
63	XM_045389014.1 PREDICTED: Macaca fascicularis interferon-induced transmembrane protein 1-like (LOC102129479), mRNA
64	XR_985755.1 PREDICTED: Macaca nemestrina interferon-induced transmembrane protein 3 pseudogene (LOC105479207), misc_RNA
65	XR_977922.2 PREDICTED: Macaca nemestrina interferon-induced transmembrane protein 3 pseudogene (LOC105466036), misc_RNA
66	XM_011741516.1 PREDICTED: Macaca nemestrina interferon-induced transmembrane protein 3 (LOC105481783), mRNA

67	XR_982613.2 PREDICTED: Macaca nemestrina interferon-induced transmembrane protein 3 pseudogene (LOC105473984), misc_RNA
68	XR_976809.2 PREDICTED: Macaca nemestrina interferon-induced transmembrane protein 3 pseudogene (LOC105464391), misc_RNA
69	XM_011729366.2 PREDICTED: Macaca nemestrina uncharacterized LOC105474566 (LOC105474566), mRNA
70	XR_986894.2 PREDICTED: Macaca nemestrina interferon-induced transmembrane protein 3 pseudogene (LOC105481311), misc_RNA
71	XR_982871.1 PREDICTED: Macaca nemestrina interferon-induced transmembrane protein 1 pseudogene (LOC105474631), misc_RNA
72	XR_634477.4 PREDICTED: Papio anubis interferon-induced transmembrane protein 3 pseudogene (LOC101024476), misc_RNA
73	XR_004176860.1 PREDICTED: Papio anubis interferon-induced transmembrane protein 3 pseudogene (LOC116269399), misc_RNA
74	XR_004179381.1 PREDICTED: Papio anubis interferon-induced transmembrane protein 3 pseudogene (LOC116271032), misc_RNA
75	XM_003901987.4 PREDICTED: Papio anubis interferon-induced transmembrane protein 3 (LOC101015605), mRNA
76	XR_636599.4 PREDICTED: Papio anubis interferon-induced transmembrane protein 3 pseudogene (LOC101001352), misc_RNA
77	XR_002517966.2 PREDICTED: Papio anubis interferon-induced transmembrane protein 3 pseudogene (LOC101026647), misc_RNA
78	XR_651947.4 PREDICTED: Papio anubis interferon-induced transmembrane protein 3 pseudogene (LOC101016949), misc_RNA
79	XR_652876.3 PREDICTED: Papio anubis interferon-induced transmembrane protein 3 pseudogene (LOC101024720), misc_RNA
80	XR_002516296.2 PREDICTED: Papio anubis interferon-induced transmembrane protein 3 pseudogene (LOC110740947), misc_RNA
81	XR_004176202.1 PREDICTED: Papio anubis interferon-induced transmembrane protein 3 pseudogene (LOC116268933), misc_RNA
82	XR_002519128.2 PREDICTED: Papio anubis interferon-induced transmembrane protein 1 pseudogene (LOC110741993), misc_RNA
83	XM_037993185.1 PREDICTED: Chlorocebus sabaeus interferon-induced transmembrane protein 3-like (LOC103243415), mRNA
84	XM_037987998.1 PREDICTED: Chlorocebus sabaeus interferon-induced transmembrane protein 3-like (LOC119620499), mRNA
85	XM_037986032.1 PREDICTED: Chlorocebus sabaeus interferon-induced transmembrane protein 3-like (LOC103230771), mRNA
86	XM_037997654.1 PREDICTED: Chlorocebus sabaeus interferon-induced transmembrane protein 3-like (LOC103223666), mRNA
87	XM_037998196.1 PREDICTED: Chlorocebus sabaeus interferon-induced transmembrane protein 3-like (LOC103224222), mRNA
88	XM_038007384.1 PREDICTED: Chlorocebus sabaeus interferon-induced transmembrane protein 3-like (LOC103223365), mRNA
89	XM_007986748.2 PREDICTED: Chlorocebus sabaeus interferon-induced transmembrane protein 3 (LOC103229029), mRNA
90	XM_008002932.2 PREDICTED: Chlorocebus sabaeus interferon-induced transmembrane protein 1 (LOC103238018), mRNA
91	XM_037986061.1 PREDICTED: Chlorocebus sabaeus interferon-induced transmembrane protein 1-like (LOC103230828), mRNA
92	XR_005241419.1 PREDICTED: Chlorocebus sabaeus interferon-induced transmembrane protein 3-like (LOC103217946), misc_RNA
93	XM_037999883.1 PREDICTED: Chlorocebus sabaeus interferon-induced transmembrane protein 3-like (LOC119624805), mRNA
94	XR_005239686.1 PREDICTED: Chlorocebus sabaeus interferon-induced transmembrane protein 3-like (LOC103241750), misc_RNA
95	XM_038004310.1 PREDICTED: Chlorocebus sabaeus interferon-induced transmembrane protein 3-like (LOC103225363), mRNA
96	XR_005239862.1 PREDICTED: Chlorocebus sabaeus interferon-induced transmembrane protein 3-like (LOC103239130), misc_RNA
97	XR_003115394.1 PREDICTED: Theropithecus gelada interferon-induced transmembrane protein 3 pseudogene (LOC112605356), misc_RNA
98	XR_003120924.1 PREDICTED: Theropithecus gelada interferon-induced transmembrane protein 3 pseudogene (LOC112630736), transcript variant X1, misc_RNA
99	XR_003116221.1 PREDICTED: Theropithecus gelada interferon-induced transmembrane protein 3 pseudogene (LOC112609612), misc_RNA

100	XR_003121812.1 PREDICTED: Theropithecus gelada interferon-induced transmembrane protein 3 pseudogene (LOC112634730), misc_RNA
101	XM_025391895.1 PREDICTED: Theropithecus gelada interferon-induced transmembrane protein 3 (LOC112628719), mRNA
102	XR_003121816.1 PREDICTED: Theropithecus gelada interferon-induced transmembrane protein 3 pseudogene (LOC112634750), misc_RNA
103	XR_003121593.1 PREDICTED: Theropithecus gelada interferon-induced transmembrane protein 3 pseudogene (LOC112633818), misc_RNA
104	XR_003115342.1 PREDICTED: Theropithecus gelada interferon-induced transmembrane protein 3 pseudogene (LOC112605064), misc_RNA
105	XR_003116523.1 PREDICTED: Theropithecus gelada interferon-induced transmembrane protein 1 pseudogene (LOC112611007), misc_RNA
106	XR_003121736.1 PREDICTED: Theropithecus gelada interferon-induced transmembrane protein 3 pseudogene (LOC112634120), misc_RNA
107	XR_003121871.1 PREDICTED: Theropithecus gelada interferon-induced transmembrane protein 3-like (LOC112635185), misc_RNA
108	XR_001001345.1 PREDICTED: Colobus angolensis palliatus interferon-induced transmembrane protein 1 pseudogene (LOC105508844), misc_RNA
109	XR_001003274.1 PREDICTED: Colobus angolensis palliatus interferon-induced transmembrane protein 1 pseudogene (LOC105521471), misc_RNA
110	XR_001001467.1 PREDICTED: Colobus angolensis palliatus interferon-induced transmembrane protein 3 pseudogene (LOC105509653), misc_RNA
111	XR_001003416.1 PREDICTED: Colobus angolensis palliatus interferon-induced transmembrane protein 3 pseudogene (LOC105522263), misc_RNA
112	XR_001003340.1 PREDICTED: Colobus angolensis palliatus interferon-induced transmembrane protein 3 pseudogene (LOC105521825), misc_RNA
113	XR_001001690.1 PREDICTED: Colobus angolensis palliatus interferon-induced transmembrane protein 3 pseudogene (LOC105511254), misc_RNA
114	XR_001004262.1 PREDICTED: Colobus angolensis palliatus interferon-induced transmembrane protein 3 pseudogene (LOC105527742), misc_RNA
115	XR_001003217.1 PREDICTED: Colobus angolensis palliatus interferon-induced transmembrane protein 3 pseudogene (LOC105521075), misc_RNA
116	XM_011961854.1 PREDICTED: Colobus angolensis palliatus interferon-induced transmembrane protein 3 (LOC105525998), mRNA
117	XR_001002890.1 PREDICTED: Colobus angolensis palliatus interferon-induced transmembrane protein 3 pseudogene (LOC105518865), misc_RNA
118	XR_001001741.1 PREDICTED: Colobus angolensis palliatus interferon-induced transmembrane protein 2 pseudogene (LOC105511638), misc_RNA
119	XR_001001127.1 PREDICTED: Colobus angolensis palliatus interferon-induced transmembrane protein 3 pseudogene (LOC105507581), misc_RNA
120	XM_026449272.2 PREDICTED: Piliocolobus tephrosceles interferon-induced transmembrane protein 3 (LOC113220337), mRNA
121	XM_023195614.3 PREDICTED: Piliocolobus tephrosceles interferon-induced transmembrane protein 3 (LOC111528796), mRNA
122	XR_002733564.3 PREDICTED: Piliocolobus tephrosceles interferon-induced transmembrane protein 3 pseudogene (LOC111548972), misc_RNA
123	XR_002732677.2 PREDICTED: Piliocolobus tephrosceles interferon-induced transmembrane protein 3 pseudogene (LOC111546183), misc_RNA
124	XR_002731406.1 PREDICTED: Piliocolobus tephrosceles interferon-induced transmembrane protein 3 pseudogene (LOC111541943), misc_RNA
125	XR_002731926.2 PREDICTED: Piliocolobus tephrosceles interferon-induced transmembrane protein 1 pseudogene (LOC111543627), misc_RNA
126	XM_023183438.2 PREDICTED: Piliocolobus tephrosceles interferon-induced transmembrane protein 1 (LOC111520572), mRNA
127	XR_002732401.2 PREDICTED: Piliocolobus tephrosceles interferon-induced transmembrane protein 1 pseudogene (LOC111545329), misc_RNA
128	XR_001889783.1 PREDICTED: Rhinopithecus bieti interferon-induced transmembrane protein 3 pseudogene (LOC108544264), misc_RNA
129	XR_001885165.1 PREDICTED: Rhinopithecus bieti interferon-induced transmembrane protein 3 pseudogene (LOC108528464), misc_RNA
130	XM_017847863.1 PREDICTED: Rhinopithecus bieti interferon-induced transmembrane protein 3-like (LOC108512578), mRNA
131	XR_001886971.1 PREDICTED: Rhinopithecus bieti interferon-induced transmembrane protein 1 pseudogene (LOC108534422), misc_RNA
132	XR_001880867.1 PREDICTED: Rhinopithecus bieti interferon-induced transmembrane protein 3 pseudogene (LOC108513356), misc_RNA

133	XR_001884255.1 PREDICTED: Rhinopithecus bieti interferon-induced transmembrane protein 1 pseudogene (LOC108525322), misc_RNA
134	XR_747609.2 PREDICTED: Rhinopithecus roxellana interferon-induced transmembrane protein 3 pseudogene (LOC104659568), misc_RNA
135	XM_030922644.1 PREDICTED: Rhinopithecus roxellana interferon-induced transmembrane protein 3 (LOC115894740), mRNA
136	XR_748909.2 PREDICTED: Rhinopithecus roxellana interferon-induced transmembrane protein 3 pseudogene (LOC104668909), misc_RNA
137	XR_004059464.1 PREDICTED: Rhinopithecus roxellana interferon-induced transmembrane protein 3 pseudogene (LOC115899880), misc_RNA
138	XR_004057469.1 PREDICTED: Rhinopithecus roxellana interferon-induced transmembrane protein 3 pseudogene (LOC115897696), misc_RNA
139	XR_004052498.1 PREDICTED: Rhinopithecus roxellana interferon-induced transmembrane protein 3 pseudogene (LOC104673756), misc_RNA
140	XR_750535.2 PREDICTED: Rhinopithecus roxellana interferon-induced transmembrane protein 1 pseudogene (LOC104681065), misc_RNA
141	XR_750288.2 PREDICTED: Rhinopithecus roxellana interferon-induced transmembrane protein 1 pseudogene (LOC104679193), misc_RNA
142	XR_001106000.2 PREDICTED: Aotus nancymaae interferon-induced transmembrane protein 3 pseudogene (LOC105711420), misc_RNA
143	XR_002477898.1 PREDICTED: Aotus nancymaae interferon-induced transmembrane protein 3 pseudogene (LOC105706694), misc_RNA
144	XR_001104807.2 PREDICTED: Aotus nancymaae interferon-induced transmembrane protein 3 pseudogene (LOC105706305), misc_RNA
145	XR_002478306.1 PREDICTED: Aotus nancymaae interferon-induced transmembrane protein 3 pseudogene (LOC105730841), misc_RNA
146	XR_001106643.2 PREDICTED: Aotus nancymaae interferon-induced transmembrane protein 3 pseudogene (LOC105713798), misc_RNA
147	XR_002478805.1 PREDICTED: Aotus nancymaae interferon-induced transmembrane protein 3 pseudogene (LOC105723281), misc_RNA
148	XR_001109428.2 PREDICTED: Aotus nancymaae interferon-induced transmembrane protein 3 pseudogene (LOC105726080), misc_RNA
149	XM_021677289.1 PREDICTED: Aotus nancymaae interferon-induced transmembrane protein 3-like (LOC105730448), mRNA
150	XM_012455723.1 PREDICTED: Aotus nancymaae putative dispanin subfamily A member 2d (LOC105719583), mRNA
151	XR_002477520.1 PREDICTED: Aotus nancymaae interferon-induced transmembrane protein 3 pseudogene (LOC110567062), misc_RNA
152	XR_001108052.2 PREDICTED: Aotus nancymaae uncharacterized LOC105719844 (LOC105719844), ncRNA
153	XM_039464032.1 PREDICTED: Saimiri boliviensis boliviensis interferon-induced transmembrane protein 3-like (LOC101044242), mRNA
154	XM_039468571.1 PREDICTED: Saimiri boliviensis boliviensis interferon-induced transmembrane protein 3-like (LOC104651646), mRNA
155	XM_039460874.1 PREDICTED: Saimiri boliviensis boliviensis interferon-induced transmembrane protein 3-like (LOC101050572), mRNA
156	XM_039467216.1 PREDICTED: Saimiri boliviensis boliviensis interferon-induced transmembrane protein 3-like (LOC120363003), mRNA
157	XM_039461850.1 PREDICTED: Saimiri boliviensis boliviensis interferon-induced transmembrane protein 3-like (LOC120360840), mRNA
158	XM_039460060.1 PREDICTED: Saimiri boliviensis boliviensis interferon-induced transmembrane protein 3-like (LOC101040174), mRNA
159	XM_039472141.1 PREDICTED: Saimiri boliviensis boliviensis interferon-induced transmembrane protein 3 (LOC120364980), mRNA
160	XM_039466275.1 PREDICTED: Saimiri boliviensis boliviensis interferon-induced transmembrane protein 3-like (LOC120362742), mRNA
161	XM_039466276.1 PREDICTED: Saimiri boliviensis boliviensis interferon-induced transmembrane protein 3-like (LOC120362743), mRNA
162	XM_039473093.1 PREDICTED: Saimiri boliviensis boliviensis interferon-induced transmembrane protein 3-like (LOC101037636), mRNA
163	XM_039464937.1 PREDICTED: Saimiri boliviensis boliviensis interferon-induced transmembrane protein 3-like (LOC101040764), mRNA
164	XM_039473337.1 PREDICTED: Saimiri boliviensis boliviensis interferon-induced transmembrane protein 3-like (LOC101049171), mRNA
165	XM_039478903.1 PREDICTED: Saimiri boliviensis boliviensis interferon-induced transmembrane protein 3-like (LOC120367594), mRNA

166	XM_039470622.1 PREDICTED: Saimiri boliviensis boliviensis interferon-induced transmembrane protein 3-like (LOC101030291), mRNA
167	XM_039459986.1 PREDICTED: Saimiri boliviensis boliviensis interferon-induced transmembrane protein 3-like (LOC101045919), mRNA
168	XM_039461847.1 PREDICTED: Saimiri boliviensis boliviensis interferon-induced transmembrane protein 3-like (LOC120360839), mRNA
169	XM_039464070.1 PREDICTED: Saimiri boliviensis boliviensis interferon-induced transmembrane protein 3-like (LOC101045171), mRNA
170	XM_039477739.1 PREDICTED: Saimiri boliviensis boliviensis interferon-induced transmembrane protein 3-like (LOC101028783), mRNA
171	XM_039464430.1 PREDICTED: Saimiri boliviensis boliviensis interferon-induced transmembrane protein 3-like (LOC120362181), mRNA
172	XM_039467413.1 PREDICTED: Saimiri boliviensis boliviensis interferon-induced transmembrane protein 3-like (LOC120363195), mRNA
173	XM_039464938.1 PREDICTED: Saimiri boliviensis boliviensis interferon-induced transmembrane protein 3-like (LOC101041094), mRNA
174	XM_035272028.1 PREDICTED: Callithrix jacchus interferon-induced transmembrane protein 3-like (LOC118147209), mRNA
175	XM_035291270.1 PREDICTED: Callithrix jacchus interferon-induced transmembrane protein 3-like (LOC103791528), mRNA
176	XM_035297088.1 PREDICTED: Callithrix jacchus interferon-induced transmembrane protein 3-like (LOC108591335), mRNA
177	XM_035274832.1 PREDICTED: Callithrix jacchus interferon-induced transmembrane protein 3-like (LOC118147795), mRNA
178	XM_035274961.1 PREDICTED: Callithrix jacchus interferon-induced transmembrane protein 3 (LOC103788209), mRNA
179	XM_035274298.1 PREDICTED: Callithrix jacchus interferon-induced transmembrane protein 3-like (LOC103788419), mRNA
180	XM_035295638.1 PREDICTED: Callithrix jacchus interferon-induced transmembrane protein 3-like (LOC108589315), mRNA
181	XM_035262187.1 PREDICTED: Callithrix jacchus interferon-induced transmembrane protein 3-like (LOC100406127), mRNA
182	XM_035297872.1 PREDICTED: Callithrix jacchus interferon-induced transmembrane protein 3-like (LOC118153442), mRNA
183	XM_035260161.1 PREDICTED: Callithrix jacchus interferon-induced transmembrane protein 3-like (LOC100392960), mRNA
184	XM_035255126.1 PREDICTED: Callithrix jacchus interferon-induced transmembrane protein 3-like (LOC100385838), mRNA
185	XM_035291278.1 PREDICTED: Callithrix jacchus interferon-induced transmembrane protein 3-like (LOC118151486), mRNA
186	XM_035277278.1 PREDICTED: Callithrix jacchus interferon-induced transmembrane protein 3-like (LOC100412785), mRNA
187	XM_035253065.1 PREDICTED: Callithrix jacchus interferon-induced transmembrane protein 3-like (LOC108590093), mRNA
188	XM_035279210.1 PREDICTED: Callithrix jacchus interferon-induced transmembrane protein 3-like (LOC108589044), mRNA
189	XM_035259315.1 PREDICTED: Callithrix jacchus interferon-induced transmembrane protein 3-like (LOC118144827), mRNA
190	XM_008993878.3 PREDICTED: Callithrix jacchus interferon-induced transmembrane protein 3-like (LOC100395060), mRNA
191	XM_035254054.1 PREDICTED: Callithrix jacchus interferon-induced transmembrane protein 3-like (LOC100402056), mRNA
192	XM_035266254.1 PREDICTED: Callithrix jacchus interferon-induced transmembrane protein 3-like (LOC100391872), mRNA
193	XR_004737475.1 PREDICTED: Callithrix jacchus uncharacterized LOC103789434 (LOC103789434), ncRNA
194	XM_035269777.1 PREDICTED: Callithrix jacchus interferon-induced transmembrane protein 3-like (LOC118146681), mRNA
195	XM_017971086.2 PREDICTED: Callithrix jacchus putative dispanin subfamily A member 2d (LOC100397238), mRNA
196	XM_035266250.1 PREDICTED: Callithrix jacchus interferon-induced transmembrane protein 3-like (LOC100401552), mRNA
197	XM_035302816.1 PREDICTED: Callithrix jacchus protein tyrosine phosphatase receptor type A (PTPRA), transcript variant X9, mRNA
198	XM_035270374.1 PREDICTED: Callithrix jacchus putative UPF0607 protein ENSP00000332738 (LOC118146801), mRNA

199	XM_037741387.1 PREDICTED: Cebus imitator interferon-induced transmembrane protein 3-like (LOC108310121), mRNA
200	XM_037740737.1 PREDICTED: Cebus imitator interferon-induced transmembrane protein 3-like (LOC108308666), mRNA
201	XM_037732364.1 PREDICTED: Cebus imitator interferon-induced transmembrane protein 3-like (LOC108290294), mRNA
202	XM_037744520.1 PREDICTED: Cebus imitator interferon-induced transmembrane protein 3-like (LOC108315712), mRNA
203	XM_037742341.1 PREDICTED: Cebus imitator interferon-induced transmembrane protein 3-like (LOC108311773), mRNA
204	XR_001829005.2 PREDICTED: Cebus imitator interferon-induced transmembrane protein 3-like (LOC108313260), misc_RNA
205	XR_001823123.2 PREDICTED: Cebus imitator interferon-induced transmembrane protein 3-like (LOC108299801), misc_RNA
206	XM_037741506.1 PREDICTED: Cebus imitator interferon-induced transmembrane protein 3-like (LOC119472443), mRNA
207	XR_001824764.2 PREDICTED: Cebus imitator interferon-induced transmembrane protein 3-like (LOC108303279), misc_RNA
208	XM_037736783.1 PREDICTED: Cebus imitator interferon-induced transmembrane protein 3-like (LOC108300669), mRNA
209	XR_001819667.2 PREDICTED: Cebus imitator interferon-induced transmembrane protein 3-like (LOC108290678), misc_RNA
210	XM_037737645.1 PREDICTED: Cebus imitator interferon-induced transmembrane protein 3-like (LOC108302525), mRNA
211	XM_037734853.1 PREDICTED: Cebus imitator interferon-induced transmembrane protein 3-like (LOC108295997), mRNA
212	XM_037745794.1 PREDICTED: Cebus imitator interferon-induced transmembrane protein 3-like (LOC108318049), mRNA
213	XM_037732737.1 PREDICTED: Cebus imitator interferon-induced transmembrane protein 3-like (LOC108291862), mRNA
214	XM_037732501.1 PREDICTED: Cebus imitator interferon-induced transmembrane protein 3-like (LOC108291366), mRNA
215	XR_001824908.1 PREDICTED: Cebus imitator interferon-induced transmembrane protein 3-like (LOC108303574), misc_RNA
216	XM_017509934.2 PREDICTED: Cebus imitator uncharacterized LOC108290196 (LOC108290196), mRNA
217	XM_037728719.1 PREDICTED: Cebus imitator interferon-induced transmembrane protein 3-like (LOC108284417), mRNA
218	XR_005196933.1 PREDICTED: Cebus imitator interferon-induced transmembrane protein 3-like (LOC119468274), misc_RNA
219	XM_037740055.1 PREDICTED: Cebus imitator interferon-induced transmembrane protein 3-like (LOC108305978), mRNA
220	XM_037739722.1 PREDICTED: Cebus imitator interferon-induced transmembrane protein 3-like (LOC108306657), mRNA
221	XM_037727362.1 PREDICTED: Cebus imitator interferon-induced transmembrane protein 3-like (LOC108281260), mRNA
222	XM_037740637.1 PREDICTED: Cebus imitator interferon-induced transmembrane protein 3-like (LOC119472128), mRNA
223	XM_037727289.1 PREDICTED: Cebus imitator interferon-induced transmembrane protein 3-like (LOC108281396), mRNA
224	XM_037735992.1 PREDICTED: Cebus imitator interferon-induced transmembrane protein 3-like (LOC108299340), mRNA
225	XR_504221.2 PREDICTED: Carlito syrichta interferon-induced transmembrane protein 3 pseudogene (LOC103259173), misc_RNA
226	XR_002482247.1 PREDICTED: Carlito syrichta interferon-induced transmembrane protein 3 pseudogene (LOC103265445), misc_RNA
227	XR_504039.2 PREDICTED: Carlito syrichta interferon-induced transmembrane protein 3 pseudogene (LOC103255732), misc_RNA
228	XR_002482145.1 PREDICTED: Carlito syrichta interferon-induced transmembrane protein 3 pseudogene (LOC103263524), misc_RNA
229	XM_008048196.1 PREDICTED: Carlito syrichta interferon-induced transmembrane protein 3-like (LOC103249564), mRNA
230	XM_008052862.1 PREDICTED: Carlito syrichta interferon-induced transmembrane protein 3-like (LOC103254874), mRNA
231	XM_008074487.1 PREDICTED: Carlito syrichta interferon-induced transmembrane protein 3-like (LOC103277049), mRNA

232	XR_504917.1 PREDICTED: Carlito syrichta interferon-induced transmembrane protein 3 pseudogene (LOC103269752), misc_RNA
233	XM_008048195.2 PREDICTED: Carlito syrichta interferon-induced transmembrane protein 1-like (LOC103249563), mRNA
234	XM_008048197.2 PREDICTED: Carlito syrichta interferon-induced transmembrane protein 3-like (LOC103249565), mRNA
235	XR_001161573.1 PREDICTED: Otolemur garnettii interferon-induced transmembrane protein 3 pseudogene (LOC100959247), misc_RNA

Supplement: Genomic localization of scattered primate IFITMs

Table S2: Genomic localization of scattered primate IFITMs

No.		Direction	Flanking gene	Direction	Annotated	Accession number	Direction	Flackin g gene	In intron	Direction	Gene
	**Homo sapiens**										
1	Chr. 4	<	EHPA5	>	IFITM3P1 pseudo	NG_006204.1	<	CENPC	x		
2	Chr. 12	<	AMN1	>	IFITM3P2 pseudo	NG_006205.3	>	RESF1	x		
3	Chr. 6	<	SPDEF	<	IFITM3P3 pseudo	NG_006229.1	<	ILRUN	x		
4	Chr. 7	<	NUPR2	>	IFITM3P4 pseudo	NG_006223.3	<	ZNF479	x		
5	Chr. 12	>	SH2B3	>	IFITM3P5 pseudo	NG_006225.2	<	BRAP	in	<	ATXN2
6	Chr. 12	<	AMIGO2	<	IFITM3P6 pseudo	NG_006230.2	<	RPAP3	in	>	PCED1 B
7	Chr. 1	>	SYF2	>	IFITM3P7 pseudo	NG_006227.1	>	RUNX3	x		
8	Chr. 8	>	CDH7	>	IFITM3P8	NG_006224.1	>	CLVS1	x		
9	Chr. 2	>	PAPLOG	>	IFITM3P9	NG_006228.3	>	BCL11A	x		
10	Chr. 6	>	HLA-F	<	IFITM4p	NR_001590.1	>	HLA-G	x		
11	Chr.8	>	YTHDF3	>	IFITM8P pseudo	NG_005307.4	>	BHLHE2 2	x		
12	Chr. 11	>	MYEOV	<	IFITM9P pseudo	NG_006210.1	>	CCND1	x		
	**Pan paniscus**										
1	Chr. 12	<	AMN1	>	IFITM3P2 pseudo	XM_003813732. 5	>	RESF1	x		
2	Chr. 12	>	SH2B3	>	IFITM3P5 pseudo	XM_034934516. 1	<	BRAP	in	<	ATXN2
3	Chr. 2a	>	PAPLOG	>	IFITM3P9	XM_034953202. 1	>	BCL11A	x		
4	Chr. 6	>	HLA-F related	<	IFITM4p	XM_034961680. 1	>	HLA-G	x		
5	Chr.8	>	YTHDF3	>	IFITM8P pseudo	XM_034966156. 1	>	BHLHE2 2	x		
	**Pan troglodytes**										
1	Chr. 4	<	EHPA5	>	IFITM3P1 pseudo	XR_001716631. 2	<	CENPC	x		
2	Chr. 12	<	AMN1	>	IFITM3P2 pseudo	XM_003952225. 4	>	RESF1	x		

3	Chr. 7	<	NUPR2	>	IFITM3P4 pseudo	XR_169790.4	<	ZNF479	x		
4	Chr. 2a	>	PAPLOG	>	IFITM3P9	XR_001715794.1	>	BCL11A	x		
5	Chr. 6	>	HLA-F related	<	IFITM4p	XR_002944366.1	>	HLA-G	x		
	**Gorilla gorilla**										
1	Chr. 12	<	AMN1	>	IFITM3P2 pseudo	XM_004052942.3	>	RESF1	x		
2	Chr. 2a	>	PAPLOG	>	IFITM3P9	XR_002004539.2	>	BCL11A	x		
3	Chr. 4	>	SHROOM3	>	3 ps	XR_002005707.2	<	SOWAHB	x		
	**Pongo abelii**										
1	Chr. 12	<	AMN1	>	IFITM3P2 pseudo	XR_656249.2	>	RESF1	x		
2	Chr. 7	<	NUPR2	>	IFITM3P4 pseudo	XR_002913425.1	<	ZNF479	x		
3	Chr. 2a	>	PAPLOG	>	IFITM3P9	XR_654203.1	>	BCL11A	x		
4	Chr. 11	>	MYEOV	<	IFITM9P pseudo	XR_656019.2	>	CCND1	x		
	**Nomascus leucogenys**										
1	Chr. 14	>	PAPLOG	>	IFITM3P9	XR_001114400.1	>	BCL11A	x		
2	Chr. 22a	<	SPDEF	<	IFITM3P3 pseudo	XM_030803316.1	<	ILRUN	x		
3	Chr. 5	<	MARC2	>	1 like	XM_030812847.1	<	MARK1	x		
4	Chr. 22a	>	EFCAB11	<	3 ps	XR_004027821.1	>	FOXN3	x		
5	Chr. 17	>	CHCHD2	>	IFITM3P4 pseudo	XR_004026378.1	>	VOPP1	x		
	**Macaca mulatta**										
1	Chr. 16	<	CTC1	<	3 like 8 (owm P1)	XM_001112566.4	>	RANGRF	in	>	PFAS
2	Chr. 1	>	ATP5PB	>	3 like (owm P2)	XM_001106166.4	>	RAP1A	in	<	TMIGD3
3	Chr. 7	>	MAPK1IP1L	<	3 like (own P3)	XM_001088204.4	>	LGALS3	x		
4	Chr. 10	<	SLC25A17	>	3 ps (owm P4)	XR_001447798.2	>	XPNPEP3	in	<	ST13
5	Chr. 12	<	KCNE4	>	3 ps	XR_001438791.2	<	SCG2	x		
6	Chr. 9	<	OIT1	>	3 ps	XR_001447146.2	>	MICU1	in	<	MCU
7	Chr. 11	>	SH2B3	>	P5	XR_001448216.2	<	BRAP	in	<	ATXN2
8	Chr.1	>	SYF2	>	P7	XM_001113914.2	>	RUNX3	x		
	**Cercocebus atys**										
1	Unplaced	<	ETV6	>	3 ps	XR_001014764.1	>	SPRP1 like	x		
2	Unplaced	>	ATP5PB	>	3 like (owm P2)	XR_001017992.1	>	RAP1A	in	<	TMIGD1
3	Unplaced		End	>	3 ps	XR_001010903.1	>	TAB3	in	>	dystropine-like
4	Unplaced	<	CTC1	<	3 like 8 (owm P1)	XM_012051598.1	>	RANGRF	in	>	PFAS
5	Unplaced	>	DYSF	<	3 ps	XR_001011714.1	<	CYP26B1	x		
6	Unplaced	<	SLC25A17	>	3 ps (owm P4)	XR_001010110.1	>	XPNPEP3	in	<	ST13

7	Unplaced	>	MAPK1IP1L	<	3 like (own P3)	XM_012061884.1	>	LGALS3	x		
8	Unplaced	>	SH2B3	>	P5	XR_001010401.1	<	BRAP	in	<	ATXN2
9	Unplaced	>	PAPLOG	>	P9	XR_001011610.1	>	BCL11A	x		
10	Unplaced	<	CHRM3	>	1 like (owm P5)	XR_001011033.1	>	ZP4	x		
	**Rhinopithecus roxellana**										
1	Chr. 12	<	WTIP	>	3 ps	XR_747609.2	<	PDCD2L	in	<	UBA2
2	Chr. 19	<	C1QL1	>	3 like	XM_030922644.1	>	NMT1	in	<	DCAKD
3	Chr. 15	<	CADM1	>	3 ps	XR_748909.2	<	BUD13	x		
4	Chr. 10	>	WASHC4	>	3 ps	XR_004059464.1	<	NUAK1	in	<	APPL2
5	Chr. 5	<	ATP10A	>	3 ps	XR_004057469.1	>	GABRB5	in	<	GABRB3
6	Chr. 13	<	SLC25A17	>	3 ps	XR_004052498.1	>	XPNPEP3	in	<	ST13
7	Chr. 8	<	SETB1	<	1 ps	XR_750535.2	>	ARNT	x		
8	Chr. 8	<	CHRM3	>	1 like (owm P5)	XR_750288.2	>	ZP4	x		
	**Aotus nancymaae**										
1	Unplaced		End	>	3 ps	XR_001106000.2	End		x		
2	Unplaced	<	ACR	>	3 ps	XR_002477898.1	<	SHANK3	x		
3	Unplaced	>	EGF	>	3 ps	XR_001104807.2	>	ENPEP	in	<	ELOVL6
4	Unplaced	>	FGF13	<	3 ps	XR_002478306.1	<	ZIC3	x		
5	Unplaced	>	HEBP1	<	3 ps	XR_001106643.2	>	GPRC5D	x		
6	Unplaced	<	AGO2	<	3 ps (nwm P)	XR_002478805.1	<	PTK2	x		
7	Unplaced	<	FBX015	>	3 ps	XR_001109428.2	>	CNDP2	x		
8	Unplaced	<	ZBED1	<	3 like	XM_021677289.1	>	CD99	x		
9	Unplaced	<	FGF16	<	3 ps	XM_012455723.1	<	MAGEE1	x		
10	Unplaced	<	BBS2	>	3 ps	XR_002477520.1	>	MT4	x		
	**Saimiri boliviensis**										
1	Unplaced	>	SOX3	<	3 like	XM_039464032.1	>	ATP11C	x		
2	Unplaced	<	HPGDS	>	3 like	XM_039468571.1	>	PDLIM5	x		
3	Unplaced	<	WARS2	>	3 like	XM_039460874.1	>	HAO2	x		
4	Unplaced		End	>	3 like	XM_039467216.1	<	G3PDH	x		
	same as above	<	PCOLCE2	>	3 like	XM_039461850.1	<	G3PDH	x		
5	Unplaced	<	ARHGEF11	>	3 like	XM_039460060.1	<	ETV3L	x		
6	Unplaced	<	RPH3a	>	3 like	XM_039472141.1	>	RPL6	in	<	PTPN11
7	Unplaced	<	ZFP211	>	3 like	XM_039466275.1	<	ZIK	next	>	3 like
8	Unplaced	<	ZFP211	>	3 like	XM_039466276.1	<	ZIK	next	>	3 like
9	Unplaced	<	MTERF1	>	3 like	XM_039473093.1	<	CYP51A1	in	>	AKAP9

10	Unplaced	<	RNF19A	<	3 like	XM_039464937.1	<	ANKRD46	next	>	3 like
11	Unplaced	>	SPRY2	>	3 like	XM_039473337.1	<	NDFIP2	x		
12	Unplaced	<	NEBL	>	3 like	XM_039478903.1	<	MIR1915HG	x		
13	Unplaced	>	RGS7BP	>	3 like	XM_039470622.1	>	SHISAL2B	x		
14	Unplaced	<	SERINC5	>	3 like	XM_039459986.1	>	SPZ1	x		
15	Unplaced	>	LRRFIP2	>	3 like	XM_039461847.1	>	EPM2AIP1	in	<	MLH1
16	Unplaced	<	ISGF1	>	3 like	XM_039464070.1	>	STK26	x		
17	Unplaced	<	DLG5	>	3 like	XM_039477739.1	<	POLR3A	x		
18	Unplaced	<	AGO2	<	3 ps (nwm P)	XM_039464430.1	<	PTK2	x		
19	Unplaced	<	KIAA1586	>	3 like	XM_039467413.1	>	DST	in	<	BEND6
20	Unplaced	<	RNF19A	<	3 like	XM_039464938.1	<	ANKRD46	next	>	3 like
	**Carlito syrichta**										
1	Unplaced	>	SVOPL	>	3 ps	XR_504221.2	End		in	<	Trim24
2	Unplaced	<	TSN3	<	3 ps	XR_002482247.1	End		x		
3	Unplaced	<	SRSF4	<	3 ps	XR_504039.2	<	MECR	x		
4	Unplaced	<	ZFP624	>	3 ps	XR_002482145.1	End		x		
5	Unplaced	<	UMODL1	>	3 like	XM_008052862.1	>	ZBTB21	x		
6	Unplaced	>	SPINK4	>	3 ps	XM_008074487.1	>	CHMP5	in	<	BAG1
7	Unplaced	>	ZFP28	>	3 ps	XR_504917.1	End		x		
	**Otolemur garnettii**										
1	Unplaced	<	VSP11	>	3 ps	XR_001161573.1	>	HYOU1	x		

Supplementary Figures

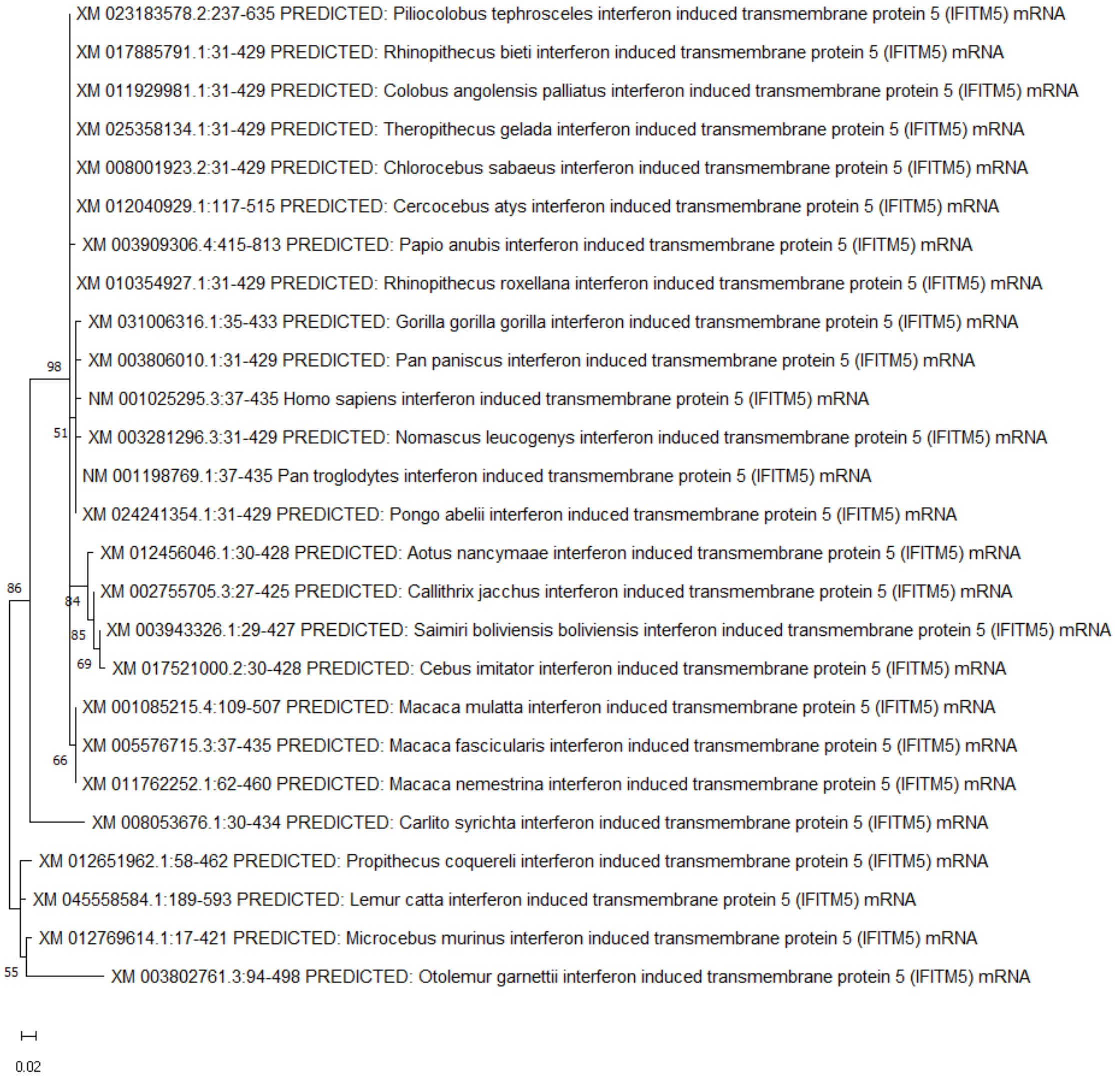

Figure S1: Phylogeny IFITM5 AA sequences: The evolutionary history was inferred by using the Maximum Likelihood method with the AA sequences. The tree is drawn to scale, with branch lengths measured in the number of substitutions per site. The percentage of trees in which the associated taxa clustered together is shown next to the branches.

IFITM5 alignment

```
                              10        20        30        40        50        60        70        80        90       100
                      ....|....|....|....|....|....|....|....|....|....|....|....|....|....|....|....|....|....|....|....|
NM 001025295.3:  1    MDTAYPREDTRAPTPSKA--GAHTALTLGAPHPPPRDHLIWSVFSTLYLNLCCLGFLALAYSIKARDQKVVGDLEAARRFGSKAKCYNILAAMWTLVPPL
XM 003806010.1:  1    .........P........--..Q.............................................................................
NM 001198769.1:  1    .........P........--................................................................................
XM 031006316.1:  1    .........P........--...........R....................................................................
XM 024241354.1:  1    .........P........--................................................................................
XM 003281296.3:  1    .........P........--............H...................................................................
XM 008053676.1:  1    ...SF....P..L..H..DGP.....AM.P.C...........................V..V.......A.............................
XM 012456046.1:  1    .........P........--.P.........Q....................................................................
XM 003943326.1:  1    .........P........--...........Q.......................................A.............................
XM 002755705.3:  1    .........P........--...........Q.......................................A.............................
XM 012040929.1:  1    .........P........--................................................................................
XM 001085215.4:  1    .........P........--R...............................................................................
XM 003909306.4:  1    .........P........--.....V..........................................................................
XM 008001923.2:  1    .........P........--................................................................................
XM 025358134.1:  1    .........P........--................................................................................
XM 011929981.1:  1    .........P........--................................................................................
XM 023183578.2:  1    .........P........--................................................................................
XM 005576715.3:  1    .........P........--R...............................................................................
XM 011762252.1:  1    .........P........--R...............................................................................
XM 017885791.1:  1    .........P........--................................................................................
XM 010354927.1:  1    .........P........--................................................................................
XM 017521000.2:  1    .........P........--...........Q.............................N.........A.............................
XM 012769614.1:  1    ...SF....PGP...R..DGA.P......T.R.......V...............L......V.......A.............................
XM 012651962.1:  1    ...SF...EP.P...R..DGA.P...A....R.......V...............L......V.......A.............................
XM 003802761.3:  1    ...SF.C..PGHL..R..DGATP........G.......V...V.S....M....L......V.........N.....H.....................
XM 045558584.1:  1    ...SF....PQP...R..DGA.P........R.......V...............L......V.......A.............................

                               110       120       130
                      ....|....|....|....|....|....|....|
NM 001025295.3:  99   LLLGLVVTGALHLARLAKDSAAFFSTKFDDADYD*
XM 003806010.1:  99   ..................................*
NM 001198769.1:  99   ..................................*
XM 031006316.1:  99   ..................................*
XM 024241354.1:  99   ..................................*
XM 003281296.3:  99   ..................................*
XM 008053676.1:  101  ........................T.....G...*
XM 012456046.1:  99   .....................T......E.G...*
XM 003943326.1:  99   ..................G..T......E.G...*
XM 002755705.3:  99   .....................T......E.G...*
XM 012040929.1:  99   ..............................G...*
XM 001085215.4:  99   ..............................G...*
XM 003909306.4:  99   ..............................G...*
XM 008001923.2:  99   ..............................G...*
XM 025358134.1:  99   ..............................G...*
XM 011929981.1:  99   ..............................G...*
XM 023183578.2:  99   ..............................G...*
XM 005576715.3:  99   ..............................G...*
XM 011762252.1:  99   ..............................G...*
XM 017885791.1:  99   ..............................G...*
XM 010354927.1:  99   ..............................G...*
XM 017521000.2:  99   ..................G..T......E.G...*
XM 012769614.1:  101  ..............................G...*
XM 012651962.1:  101  ..............................G...*
XM 003802761.3:  101  .................R............G...*
XM 045558584.1:  101  ..............................G...*
```

Figure S2: Alignment of primate IFITM5s: Alignment of the AA sequence of primate IFITM5.

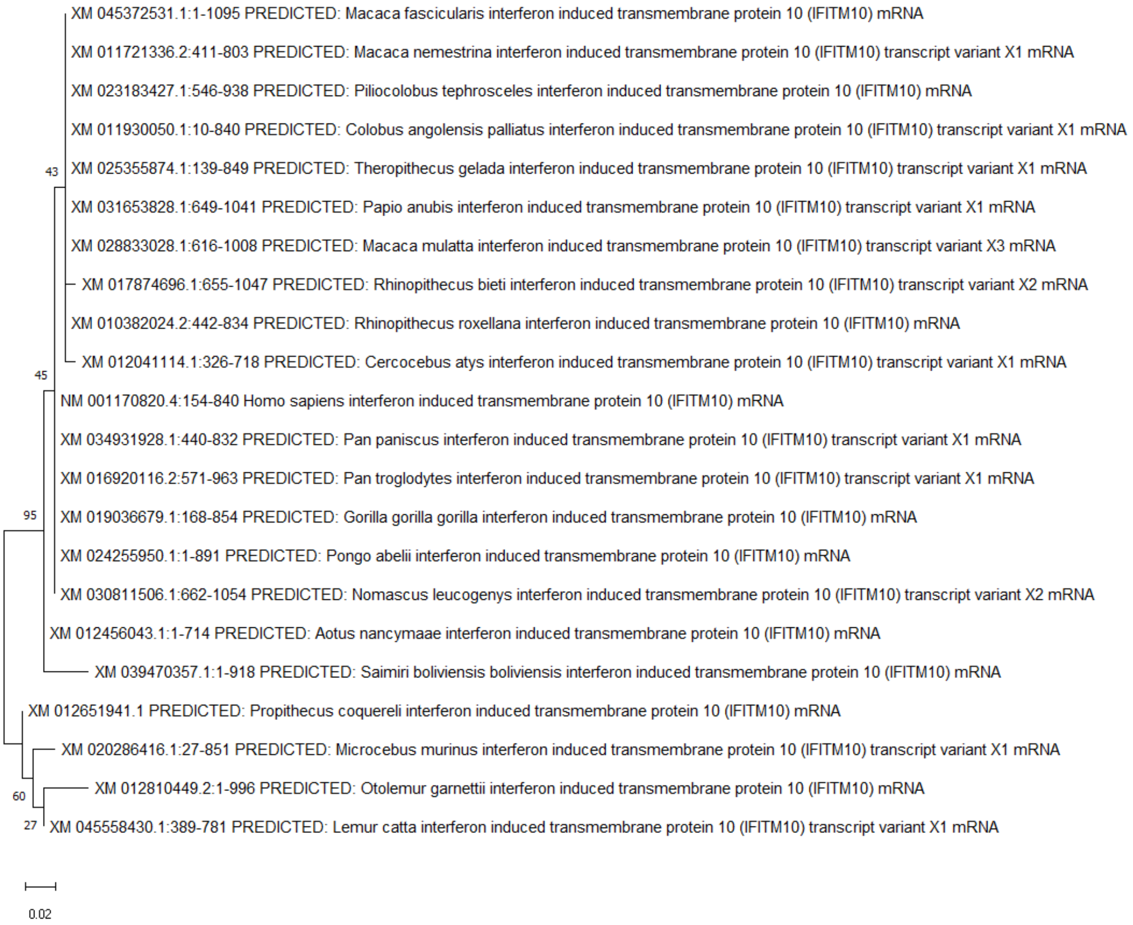

Figure S3: Phylogeny IFITM10 AA sequences: The evolutionary history was inferred by using the Maximum Likelihood method with the AA sequences. The tree is drawn to scale, with branch lengths measured in the number of substitutions per site. The percentage of trees in which the associated taxa clustered together is shown next to the branches.

Alignment IFITM 10

```
                                        10         20         30         40         50         60         70         80         90        100
                                ....|....|....|....|....|....|....|....|....|....|....|....|....|....|....|....|....|....|....|....|
NM 001170820.4:   1   MAPTLFPMESKSSKTDSVRAAGAPPACKHLAEKKTMTNPTTVIEVYPDTTEVNDYYLWSIFNFVYLNFCCLGFIALAYSLKVRDKKLLNDLNGAVEDAKT
XM 034931928.1:   1   ....................................................................................................
XM 016920116.2:   1   ....................................................................................................
XM 019036679.1:   1   ....................................................................................................
XM 024255950.1:   1   ....................................................................................................
XM 030811506.1:   1   ....................................................................................................
XM 012456043.1:   1   ....................................................................................................
XM 039470357.1:   1   ..............A.........A................I.D........................................................
XM 012041114.1:   1   ....................T...A...........................................................................
XM 028833028.1:   1   ....................T...............................................................................
XM 031653828.1:   1   ....................T...............................................................................
XM 025355874.1:   1   ....................T...............................................................................
XM 011930050.1:   1   ....................T...............................................................................
XM 023183427.1:   1   ....................T...............................................................................
XM 045372531.1:   1   ....................T...............................................................................
XM 011721336.2:   1   ....................T...............................................................................
XM 017874696.1:   1   ....................T.V.............................................................................
XM 010382024.2:   1   ....................T...............................................................................
XM 020286416.1:   1   ........................Q..........LA..A..V.....SS..................................................
XM 012651941.1    1   ........................Q...........A.....V.....SS..................................................
XM 012810449.2:   1   ....................TS..Q..........VA..A..V.....SS..................................................
XM 045558430.1:   1   ....................T...Q...........A..A..V.....SS..................................................

                                       110        120        130
                                ....|....|....|....|....|....|.
NM 001170820.4:   101 ARLFNITSSALAASCIILVFIFLRYPLTDY*
XM 034931928.1:   101 ..............................*
XM 016920116.2:   101 ..............................*
XM 019036679.1:   101 ..............................*
XM 024255950.1:   101 ..............................*
XM 030811506.1:   101 ..............................*
XM 012456043.1:   101 ..................I...........*
XM 039470357.1:   101 ..................I...........*
XM 012041114.1:   101 ..............................*
XM 028833028.1:   101 ..............................*
XM 031653828.1:   101 ..............................*
XM 025355874.1:   101 ..............................*
XM 011930050.1:   101 ..............................*
XM 023183427.1:   101 ..............................*
XM 045372531.1:   101 ..............................*
XM 011721336.2:   101 ..............................*
XM 017874696.1:   101 ..............................*
XM 010382024.2:   101 ..............................*
XM 020286416.1:   101 ..................I........S..*
XM 012651941.1    101 ..................I...........*
XM 012810449.2:   101 ...........................S..*
XM 045558430.1:   101 ..................I...........*
```

Figure S4: Alignment of primate IFITM10s: Alignment of the AA sequence of primate IFITM10.

Alignment IR-IFITMs

```
                                  10        20        30        40        50        60        70        80        90       100
                        ....|....|....|....|....|....|....|....|....|....|....|....|....|....|....|....|....|....|....|....|
NM 021034.3:48-    1    MNHTVQTFFSPVNSGQPPNYEMLKEEHEVAVLGAPHNPAPPTSTVIHIRSETSVPDHVVWSLFNTLFMNPCCLGFIAFAYSVKSRDRKMVGDVTGAQAYA
XM 012040930.1:    1    .......V..........S..................S...............................T......................L.......
XM 028832948.1:    1    .......V.P........S......................M...........................T......................L.......
XM 015113207.2:    1    ...........................D..MM............................................................L.......
XM 015113206.2:    1    ...........................D..MM............................................................L.......
XM 031662204.1:    1    .......V..........S.........................................................................L.......
XM 031653856.1:    1    .......V..........S.........................................................................L.......
XM 005576716.3:    1    .......V.P........S......................M...........................T......................L.......
XM 005576719.3:    1    ...........................D..MM............................................................L.......
XM 011762251.1:    1    .......V.P........S......................M...........................T......................L.......
XM 011762255.2:    1    ..............S...............MM............................................................L.......
XM 010354836.2:    1    .......V..........S......................M..................................................L.......
XM 017885790.1:    1    .......V.............L....Q.................................................................L.......
XM 010354744.2:    1    .......V.............L....Q..............M..................................................L.......
XM 017521001.2:    1    .........T.ASTHR.....I........A......................................S......................L.......
NM 006435.3:378    1    ...I...-..................Q...M..V.......M...........................T..............................
XM 034931794.1:    1    ...I...-..................Q...M..........M..........................................................
NM 001198767.1:    1    ...I...-..................Q...M..........M..........................................................
XM 009245970.2:    1    .......-......S...............M.............................................................M.......
XM 004050342.2:    1    ...I...-......S.........A.Q...M.................H....................T..............................
XM 034951329.1:    1    ....................................................................................................
NM 001198757.1:    1    .........................................M..........................................................
XM 004050337.3:    1    ............................................................................................M.......
XM 002821311.4:    1    ................L...........................................................................M.......
XM 003281297.4:    1    .....................V.................A....................................................L.......
XM 030801594.1:    1    .....................V.................A....................................................L.......
XM 012456047.2:    1    .........T.ASTHH..............A......................................S......................L.......
XM 039471018.1:    1    .........T.ASTDR.............D...V...............................I...S......................L.......
XM 003943324.3:    1    .........T.ASTDR.....I........A..V...................................S......................L.......
XM 039471016.1:    1    .....H...T.A.THH.....I........A..V.................A.............I...S.......Y..............L.......
XM 035263965.1:    1    .........T.ASTSR..............A......................................S......................L.......
XM 035263964.1:    1    .........T.ASTSR..D...........A......................................S......................L.......
NM 003641.5:132    1    ---------------------.H..........P.PSTIL.R....N.H..................L.W..............................
XM 034931795.1:    1    ---------------------.H............PSTIL.R....N.H..................L.W..............................
NM 001198758.1:    1    ---------------------.H............PSTIL.R....N.H..................L.W..............................
XM 004050339.2:    1    ---------------------.H............PSTIL.R....N.H..................L.W..............................
NM 001198762.1:    1    ---------------------.H......T.....PSTIL.R....N.H..I...............L.W..............................
XM 030801595.1:    1    ---------------------.H............PSTIL.R....N.H..................L.W.......Y..............L.......
XM 035263963.1:    1    ---------------------............G..SSML......N.Q.G..................W......................L.......
XM 012040931.1:    1    ---------------------.H......S......STIL.R..M.N.Q........I.......I.L.W..............................
XM 028832947.1:    1    ---------------------.H......S......STIL.R..M.N.Q........I.......I.L.W.............-X...............
XM 021677005.1:    1    ---------------------............G.QSSML.R..A...Q..A.....I...........W.............-X.......L.......
XM 011929983.1:    1    ---------------------.H.............STIL.R..M.N.Q........I.......I.L.W..............................
XM 009185182.4:    1    ---------------------.H......S......STIL.R..M.N.Q........I.......I.L.W..............................
XM 005576718.3:    1    ---------------------.H......S......STIL.R..M.N.Q........I.......I.L.W..............................
XM 011762253.1:    1    ---------------------.H......S......STIL.R..M.N.Q........I.......I.L.W..............................
XM 017885792.1:    1    ---------------------.H......S......STIL.R..M.N.Q........I.......I.L.W..............................
XM 010354645.2:    1    ---------------------.H......S......STIL.R..M.N.Q........I.......I.L.W..............................
XM 003943325.3:    1    ---------------------............G.QSSML.R....N.Q..A.............I...W......................L.......
XM 001116556.3:    1    ---------------------.I....-----.V-------K.A.T............I........V....................T...L.....C.
XM 008004069.2:    1    ---------------------.I....-----.V-------..A.T....K.....................................T...L.......
XM 011762371.2:    1    ---------------------.I....-----.V-------K.A............................................T...L.....C.
XM 025358469.1     1    ---------------------.I....-----.V-------K.A.T.......................T............N.....T...L.....C.
XM 005576720.3:    1    ---------------------.I....-----.V-------K.A.T............I.............................T...L.....C.
XM 017894043.1:    1    ---------------------.I....-----CV-------K.AMT.................................S............L.....C.
XM 010356490.2:    1    ---------------------.I....-----CV-------K.AMT.......A.........................S............L.....C.
XM_009185183.3:    1    .----SAEEACGKRD------VI.K..-----.V-------K.A.T.......................T..................T...L.....C.
XM 012652051.1:    1    ....S.P..TRAQV.L.....V....R......T..G...S......VH..................L.C...................M..L.......
XM 012651945.1:    1    ....S.P..TRAQA.L.S...G...........T..G...S......VH..................L.C...................M..L.......
XM 003802780.3:    1    ...SS.SV.TRTHT.L.....Q...........S.P....---.....HG..A................CF.....................LA..KT..
XM 003802781.3:    1    .S.SSPNL.----T.L.....TR..........S.P....---.....HG..A................CF.....................LA..KT..
XM 045558583.1:    1    ...AS.P..TRAQT.L.....V...........T..G...S......VH....................C.............A................
XM 045558582.1:    1    ...AS.P..PGAQT.L.S.H.VV..........T..G...S......VH....................C.............A................
XM_012769579.2:    1    .-S.P.P..TRAQT.LHS.S.............T..G..TS.......H.D................L.C.....V................L.....H.
XM 012769578.2:    1    .S..P....TRAQA.L.....V...........T..G..TS......VH.D.A..............L.C......................L.......
NM_030694.1:47-    1    .S.NS.A.-LST.A.L..S..TI...YG.TE..E.S.S.VVRT...NMPR.V...............F.A.....V.Y...............V......
NM_026820.3:155    1    ---------------------.P..QQ..V...S..ISTSA.A.T.NMP-.I.T...............F.....V.Y..............T.....F.
NM_025378.2:100    1    ....S.A.ITAASG.......RI...Y...EM....GS.SVRT...NMPR.V.................F.......Y......................

                                 110       120       130       140
                        ....|....|....|....|....|....|....|....|....|....
NM 021034.3:48-    101  STAKCLNIWALILGILMTILLIVI-PVL--------------IFQAYG*
XM 012040930.1:    101  .......................V-...--------------....HR*
XM 028832948.1:    101  ...................P....-...--------------.Y..HR*
XM 015113207.2:    101  .......................V-...--------------....HQ*
XM 015113206.2:    101  .......................V-...--------------....HQ*
XM 031662204.1:    101  .......S..............IV-...--------------....HR*
XM 031653856.1:    101  .......S..............IV-...--------------....HR*
XM 005576716.3:    101  ...................P....-...--------------.Y..HR*
XM 005576719.3:    101  .......................V-...--------------....HQ*
XM 011762251.1:    101  .......V...........P....-...--------------.Y..HR*
XM 011762255.2:    101  .......................V-...--------------..H.HQ*
XM 010354836.2:    101  ........................-...--------------.Y..HR*
XM 017885790.1:    101  ........................-...--------------.Y..HR*
XM 010354744.2:    101  ........................-...--------------.Y..HR*
XM 017521001.2:    101  ...............I......I.-.I.--------------.L...Q*
NM 006435.3:378    100  ...............F......I.-...--------------VV..QR*
XM 034931794.1:    100  ...............F.....VI.-...--------------VV..QR*
NM 001198767.1:    100  ...............F.....VI.-...--------------VV..QR*
XM 009245970.2:    100  ........................-...--------------.V..HR*
XM 004050342.2:    100  ...............F.....VI.-...--------------VV..QR*
XM 034951329.1:    101  ........................-...--------------......*
NM 001198757.1:    101  ........................-...--------------......*
XM 004050337.3:    101  ........................-...--------------......*
XM 002821311.4:    101  ........................-...--------------.....Q*
XM 003281297.4:    101  ......................I.-...--------------.....Q*
XM 030801594.1:    101  ........................-...--------------.V..HR*
XM 012456047.2:    101  ...............IT.......-.I.--------------.L...Q*
XM 039471018.1:    101  ...............I....M.I.-.I.--------------.L...R*
XM 003943324.3:    101  ...............I....M.I.-.I.--------------.L...R*
XM 039471016.1:    101  ...............I.I..M.I.-.I.--------------.L...R*
```

Alignment IR-IFITMs

```
XM 003943324.3:  101 ...............I....M.I.-.I.--------------.L...R*
XM 039471016.1:  101 ...............I.I..M.I.-.I.--------------.L...R*
XM 035263965.1:  101 ................T.....I.-.I.--------------.L...Q*
XM 035263964.1:  101 ................T.....I.-.I.--------------.L...Q*
NM 003641.5:132  80  ...................GF.LL-L.FGSVTVYHIMLQIIQEKRG.-*
XM 034931795.1:  80  ...................GF.LL-L.FGSVTVYHIMLQIIQEKRG.-*
NM 001198758.1:  80  ...................GF.LL-L.FGSVTVYHIMLQIIQEKRG.-*
XM 004050339.2:  80  ...................GF.LL-L.FGSVTVYHIMLQIIQEKRG.-*
NM 001198762.1:  80  ...................GF.LL-L.FGSVTVYHVMLQIVHEKRG.-*
XM 030801595.1:  80  ...................GF.LL-L.FGSVTVYHIMLQIIQEKRGD-*
XM 035263963.1:  80  ............V..I...GF.LL-L.FGSVIVYHAVSQILQNQRVHL*
XM 012040931.1:  80  ........S...V......GF.LL-L.FGSVAIYHVMLQIVQEK.R.-*
XM 028832947.1:  79  ........S...V......GF.LL-L.YGSVAIYHVMLQIVQEK.R.-*
XM 021677005.1:  79  ...............I...GF.LL-L.FGSVMLYHVVSQILNNQRVHL*
XM 011929983.1:  80  ........S...V......GF.LL-L.FGSVAIYHIMLQIVQEK.R.-*
XM 009185182.4:  80  ........S...V......GF.LL-L.FGSVAIYHVMLQIVQEK.R.-*
XM 005576718.3:  80  ........S...V......GF.LL-L.YGSVAIYHVMLQIVQEK.R.-*
XM 011762253.1:  80  ........S...V......GF.LL-L.FGSVAIYHVMLQIVQEK.R.-*
XM 017885792.1:  80  ........S...V......GF.LL-L.FGSVAIYHIMLQIIQEK.R.-*
XM 010354645.2:  80  ........S...V......GF.LL-L.FGSVAIYHIMLQIIQEK.R.-*
XM 003943325.3:  80  ............V..I...GF.LL-V.FGSVMMYHVVSRILQDQRIPF*
XM 001116556.3:  68  ...........TV...V..A.TILS..V-------------------L*
XM 008004069.2:  68  ...........TV...V..A.TILS..V-------------------L*
XM 011762371.2:  68  ...........TV...V..A.TILS..V-------------------L*
XM 025358469.1   68  ..........VTV...V..AVTILS..V-------------------L*
XM 005576720.3:  68  ...........TV...V..A.TILS..V-------------------L*
XM 017894043.1:  68  ..T........TV...V.TA.TILS..V-------------------L*
XM 010356490.2:  68  ...........TV..FV.TA.TILS..V-------------------L*
XM_009185183.3:  79  ...........TV...V.TA.TILS..V-------------------L*
XM 012652051.1:  101 .......V...V.S..G...V..A-T.V--------------SIRSFS*
XM 012651945.1:  101 .......V...V.S..G...V..A-T.V--------------SIRSFS*
XM 003802780.3:  98  ...........V.SL.G..M..I.-..I--------------VI.SR.*
XM 003802781.3:  94  ...........V.SL.G..M..I.-..I--------------VI.SR.*
XM 045558583.1:  101 .......V...V.S..G...VV..-T.V-------------VSI.SFS*
XM 045558582.1:  101 .......V...V.S..G...VV..-T.V-------------VSI.SFS*
XM_012769579.2:  100 ...........V.S..GI..M..G-M.I--------------NSRNIH*
XM 012769578.2:  101 .......V...V.S..G...I..A-T.V--------------SV.NLT*
NM_030694.1:47-  100 ........SS..FS...V.IC.I.---FSTTSVVVFQSFAQRTPHSGF*
NM_026820.3:155  79  ........SS.FFT..TA.VV..V--------------------C.IR*
NM_025378.2:100  101 ........ST.V.S...VVIT..S-VII----------IVLNAQNLHT*
```

Figure S5: Alignment of primate IR-IFITMs: Alignment of the AA sequence of primate IR-IFITMs.

N-terminus

```
                               10        20        30        40        50        60        70        80        90       100
                      ....|....|....|....|....|....|....|....|....|....|....|....|....|....|....|....|....|....|....|....|
NM 006435.3:378    1  ATGAACCACATTGTGCAAACC---TTCTCTCCTGTCAACAGCGGCCAGCCTCCCAACTACGAGATGCTCAAGGAGGAGCAGGAAGTGGCTATGCTGGGGG
XM 034931794.1:    1  .....................---..........................C...................................A.............
XM 004050342.2:    1  .....................---...................A.......C......................C.........G................
NM 001198767.1:    1  .....................---..........................C...................................A.............
XM 009245970.2:    1  ..........C...C......---...................A.......C.............................C..G................
NM 001198757.1:    1  .....T....C...C......TTC..........................C........T....................C..G......G.........
XM 004050337.3:    1  .....T....C...C......TTC..........................C........T....................C..G......G.........
XM 002821311.4:    1  ..........C...C......TTC.........................TC........T....................C..G......G.........
XM 003281297.4:    1  ..........C...C......TTC..........................C........T...G................C..G.....CG.........
NM 021034.3:48-    1  .....T....C...C......TTC.................T........C........T....................C..G......G.........
XM 030801594.1:    1  ..........C...C......TTC..........................C........T...G................C..G.....CG.........
XM 034951329.1:    1  .....T....C...C......TTC..........................C........T....................C..G......G.........
XM 012456047.2:    1  ..........C..........TTC...A....C.C..G..C.CA...T..C..G..............G........A..T..G.....CGC........
XM 039471018.1:    1  ..........C...C......TTC...A....C.C..G..C..A..GT..C..A.....T........G...........T..G....A.G.........
XM 003943324.3:    1  ..........C...C......TTC...A....C.C..G..C..A..GT..C..A.....T.....A..G...........T..G......GC........
XM 039471016.1:    1  ..........C...C..T...TTC...A....C.C.....C.CA...T..C..G.....T.....A..G...........T..G......GC........
XM 035263965.1:    1  ..........CG..C......TTC...A....C.C..G..C.A...GT..C..G.....T........G........A..T..G......GC........
XM 035263964.1:    1  ..........CG..C......TTC...A....C.C..G..C.A...GT..C..GG....T........G........A..T..G......GC........
XM 012040930.1:    1  ..........CG..C......GTC..........................C....G..................A.....C..G..A...G.........
XM 028832948.1:    1  ..........CG..C......GTC...C......................C....G...T..............A.....T..G..A...G.........
XM 015113207.2:    1  ..........CG..C......TTC...................................T..............A.....C..T.........A......
XM 015113206.2:    1  ..........CG..C......TTC...................................T..............A.....C..T.........A......
XM 031662204.1:    1  ..........CG..C......GTC..........................C....G..................A.....C..G..A...G.........
XM 031653856.1:    1  ..........CG..C......GTC..........................C....G..................A.....C..G..A...G.........
XM 005576716.3:    1  ..........CG..C......GTC...C......................C....G...T..............A.....T..G..A...G.........
XM 005576719.3:    1  ..........CG..C......TTC...................................T..............A.....C..T.........A......
XM 011762251.1:    1  ..........CG..C......GTC...C......................C....G...T..............A.....C..G..A...G.........
XM 011762255.2:    1  ..........CG..C......TTC..................A................T..............A.....C..G.........A......
XM 010354836.2:    1  ..........CG..C......GTC..............T................G...T........G.....A.....C..G......G.........
XM 017885790.1:    1  ..........CG..C......GTC..............T....................T...T................G......G.........
XM 010354744.2:    1  ..........CG..C......GTC..............T....................T...T................G......G.........
XM 017521001.2:    1  ..........C...C......TTC...A......C..G..C.CA..GT..C..G.....T.....A..G........A..T..G......GC........
NM 003641.5:132    1  ---------------------------------------------------------------....A.........A..T..G......G........C
XM 034931795.1:    1  ---------------------------------------------------------------....A.........A..T..G......G.........
NM 001198758.1:    1  ---------------------------------------------------------------....A.........A..T..G......G.........
XM 004050339.2:    1  ---------------------------------------------------------------....A.........A..T..G......G.........
NM 001198762.1:    1  ---------------------------------------------------------------....A............C..G...A..G.........
XM 030801595.1:    1  ---------------------------------------------------------------....A............C..G......G.........
XM 012040931.1:    1  ---------------------------------------------------------------....A............C......T..G.........
XM 028832947.1:    1  ---------------------------------------------------------------....A............C......T..G.........
XM 011929983.1:    1  ---------------------------------------------------------------....A............C..G......G.........
XM 009185182.4:    1  ---------------------------------------------------------------....A............C......T..G.........
XM 005576718.3:    1  ---------------------------------------------------------------....A............C......T..G.........
XM 011762253.1:    1  ---------------------------------------------------------------....A............C......T..G.........
XM 017885792.1:    1  ---------------------------------------------------------------....A............C..G...T..G.........
XM 010354645.2:    1  ---------------------------------------------------------------....A............C..G...T..G.........
XM 003943325.3:    1  ---------------------------------------------------------------..................C........GG.........
XM 021677005.1:    1  ---------------------------------------------------------------..................C..G.....AG.........
XM 035263963.1:    1  ---------------------------------------------------------------........A.........C..G.....AG.........
XM 001116556.3:    1  ---------------------------------------------------------------...A.............C---------------..T.
XM 008004069.2:    1  ---------------------------------------------------------------...A.............C---------------..T.
XM 011762371.2:    1  ---------------------------------------------------------------...A.............C---------------..T.
XM 025358469.1     1  ---------------------------------------------------------------...A.............C---------------..T.
XM 005576720.3:    1  ---------------------------------------------------------------...A.............C---------------..T.
XM 017894043.1:    1  ---------------------------------------------------------------...A.............C---------------T.T.
XM 010356490.2:    1  ---------------------------------------------------------------...A.............C---------------T.T.
XM_009185183.3:    1  ...------------TC.G.AGAGGAGG.CTG..G...G..A.A.------------------G..A.....A.......C---------------..T.
XM_012769578.2:    1  ....G.....C.CCC..G...TTC...A.C.GG.C.C.AGCT..G.TC..C..A.........G.................C..G.....CG........A
XM 012652051.1:    1  ..........C.TCC..GC..TTC...A.C.GC.C.C.AGT.....TC..C..A.....T..AG................GC..G.....CG........A
XM 012651945.1:    1  ..........C.TCC..GC..TTC...A.C.GC.C.C.AGC.....TC..CT.A.........GG................C..G.....CG....A...A
XM 003802780.3:    1  .........TC.TCC....GTGTC...A...GAAC.C...CA..G.TC..C..G.....T...CA.........A.....C........CG.......TT
XM 003802781.3:    1  ....GT...TC.TCC.C..ATCTC..-------------.CA..G.TC..C..G.....T....CA.G......A.....C........CG.......TT
XM 045558583.1:    1  .........GC.TCC..GC..TTC...A.C.GA.C.C.G.C.....TC..C..A.....T...G.................C..G.....CG........A
XM 045558582.1:    1  .........GC.TCC..GC..TTC...C..GG..C.C.G.C.....TC..CT.A...C.....G..G..............C..G.....CG........A
XM_012769579.2:    1  ...---AG..C.CCC..GC..TTC...A.C.GG.C.C.A.C.....T..ACT.A....C......................C..G.....CG........A

                             110       120       130       140       150       160       170
                      ....|....|....|....|....|....|....|....|....|....|....|....|....|....|.
NM 006435.3:378   98  TGCCCCACAACCCTGCTCCCCCGATGTCCACCGTGATCCACATCCGCAGCGAGACCTCCGTGCCTGACCAT
XM 034931794.1:   98  C..............................G................................C......
XM 004050342.2:   98  C.......................C....................A..................C......
NM 001198767.1:   98  C...............................................................C......
XM 009245970.2:   98  C.......................CA......................................C......
NM 001198757.1:  101  C................................................T..............C......
XM 004050337.3:  101  C.......................C.......................................C......
XM 002821311.4:  101  C.......................CA......................................G......
XM 003281297.4:  101  C................G......C.......................................C......
NM 021034.3:48-  101  C.......................C.......................................C......
XM 030801594.1:  101  C................G......C.......................................C......
XM 034951329.1:  101  C.......................C.......................................C......
XM 012456047.2:  101  C...............G.......CA......................................C.....C
XM 039471018.1:  101  ................A.......CA...........T........A.................C......
XM 003943324.3:  101  ................G.......CA...........T........A.................C......
XM 039471016.1:  101  ................G.......CA....................A......G..........C.....C
XM 035263965.1:  101  C...............G.......CA....................A.................C.....C
XM 035263964.1:  101  C...............G.......CA....................A.................C.....C
XM 012040930.1:  101  C..........T............C.................................T.....C......
XM 028832948.1:  101  C...............................................................C......
XM 015113207.2:  101  C.......................C...............T.......................C......
XM 015113206.2:  101  C.......................C...............T..............................
XM 031662204.1:  101  CA......................C.......................................C......
XM 031653856.1:  101  CA......................C.......................................C......
XM 005576716.3:  101  C...............................................................C......
XM 005576719.3:  101  C.......................C...............T..............................
XM 011762251.1:  101  C...............................................................C......
XM 011762255.2:  101  C.......................C...............T.......................C......
XM 010354836.2:  101  CT................................A.............................C......
XM 017885790.1:  101  C.......................C.......................................C......
XM 010354744.2:  101  C...............................................................C......
XM 017521001.2:  101  C...............G.....A.CA....................A.................G.....C
NM 003641.5:132   38  CA....C..G.A.CATC.TT..A.G.............A......A..................C......
```

```
N-terminus

XM 017521001.2:   101 C...............G.....A.CA....................A.................G.....C
NM 003641.5:132   38  CA....C..G.A.CATC.TT..A.G.............A......A..................C......
XM 034931795.1:   38  CA....C..G.A.CATC.TT..A.G.............A......A..................C......
NM 001198758.1:   38  CA....C..G.A.CATC.TT..A.G.............A......A..................C......
XM 004050339.2:   38  CA....C..G.A.CATC.TT..A.G.............A......A..................C......
NM 001198762.1:   38  CA....C..G.A.CATC.TT..A.G......T......A......A........T.........C......
XM 030801595.1:   38  CA....C..G.A.CATC.TT..A.G.............A......A..................C......
XM 012040931.1:   38  CA.....T.G.A.CATC.T...A.G.......A.....A.T....AA.................C......
XM 028832947.1:   38  CA.....T.G.A.CATC.T...A.G.......A.....A......AA.................C......
XM 011929983.1:   38  CA.....T.G.A.CATC.T...A.G.......A.....A......AA.................C......
XM 009185182.4:   38  CA.....T.G.A.CATC.T...A.G.......A.....A......AA........................
XM 005576718.3:   38  CA.....T.G.A.CATC.T...A.G.......A.....A......AA.................C......
XM 011762253.1:   38  CA.....T.G.A.CATC.T...A.G.......A.....A......AA.................C......
XM 017885792.1:   38  CA.....T.G.A.CATC.T...A.G.......A.....A......AA.................C......
XM 010354645.2:   38  CA.....T.G.A.CATC.T...A.G.......A.....A......AA.................C......
XM 003943325.3:   38  G......G.G.AGCATG.TT..C.G......T......A......AG......G..........C.....C
XM 021677005.1:   38  GC.....G.G.AGCATG.T...C.G........C...........AG......G..........C.....C
XM 035263963.1:   38  G........G.AGCATG.T...C.C.............A......AG..T.G............C.....C
XM 001116556.3:   23  ..---------------------.A...AG......C...........................C......
XM 008004069.2:   23  ..---------------------.C...AG......C.............A.............C......
XM 011762371.2:   23  ..---------------------.A...Ag..........T.......................C......
XM 025358469.1    23  ..---------------------.A...AG......C...........................C......
XM 005576720.3:   23  ..---------------------.A...AG......C...........................C......
XM 017894043.1:   23  ..---------------------.A...AG..A...C...........................C......
XM 010356490.2:   23  ..---------------------.A...AG..A...C.......................C...C......
XM_009185183.3:   56  ..---------------------.A...AG......C...........................C......
XM 012769578.2:   101 C.......GG...C..CA..T.C.CA...............G...A......C...G.......C.....C
XM 012652051.1:   101 C.......GG...C......T.C.C................G...A..................C.....C
XM 012651945.1:   101 C.......GG...C......T.C.C................G...A..................C.....C
XM 003802780.3:   101 C.....C.............---------................ATG.T......G.T.....C.....C
XM 003802781.3:   89  C.....C.............---------................ATG.T......G.T.....C.....C
XM 045558583.1:   101 C.......GG...C..G...T.C.CA...............G...A..................C.....C
XM 045558582.1:   101 C.......GG...C..G...T.C.CA...............G...A..................C.....C
XM_012769579.2:   98  C.......GG...C..CA..T.C.CT...................A......C...........C.....C
```

Figure S6: Alignment N-terminus of primate IR-IFITMs: Alignment N-terminus of the AA sequence of primate IR-IFITMs.

CD225 domain

```
                          10         20         30         40         50         60         70         80         90        100
                 ....|....|....|....|....|....|....|....|....|....|....|....|....|....|....|....|....|....|....|....|
NM_006435.3:378  1    GTGGTCTGGTCCCTGTTCAACACCCTCTTCATGAACACCTGCTGCCTGGGCTTCATAGCATTCGCGTACTCCGTGAAGTCTAGGGACAGGAAGATGGTTG
NM_021034.3:48-  1    ..C.................................C............................C..................................
XM_034931794.1:  1    ..C.................................C............................C..................................
XM_034951329.1:  1    ..C.........T.......................C............................C..................................
NM_001198767.1:  1    ..C.................................C............................C..................................
NM_001198757.1:  1    ..C.................................C............................C..................................
XM_004050342.2:  1    ..C..............................................................C..................................
XM_004050337.3:  1    ..C.................................C...............................................................
XM_002821311.4:  1    ..T.................................C............................C..................................
XM_009245970.2:  1    ..T.................................C............................C.....T............................
XM_003281297.4:  1    ..C.................................C............................C..................................
XM_030801594.1:  1    ..C.................................C............................C..................................
XM_012456047.2:  1    ..C.................................T......................G.....C................................C.
XM_039471018.1:  1    ..C.....................A...........T......................G.....C..................................
XM_003943324.3:  1    ..C.................................T......................G.....C..................................
XM_039471016.1:  1    ..C.....................A...........T......................C.A...C..................................
XM_035263965.1:  1    ..C.................................T......................G.....C..................................
XM_035263964.1:  1    ..C.................................T......................G.....C..................................
NM_003641.5:132  1    ..C...........................T.....TGG.....T....................C..................................
XM_034931795.1:  1    ..C...........................T.....TGG.....T....................C..................................
NM_001198758.1:  1    ..C...........................T.....TGG.....T....................C..................................
XM_004050339.2:  1    ..C...........................T.....TGG.....T....................C.....T............................
NM_001198762.1:  1    ..C...........................T.....TGG.....T....................C.....T............................
XM_030801595.1:  1    ..C...........................T.....TGG......................A...C..................................
XM_035263963.1:  1    ..C.................................TGG..T.................G.....C.......................A..........
XM_012040931.1:  1    A.C.....................A.....T.....TGG....................G.....C.....T............................
XM_028832947.1:  1    A.C.....................A.....T.....TGG..........................C............-----.................
XM_021677005.1:  1    A.C.................................TGG....................C.....C............-----...............C.
XM_011929983.1:  1    A.C.....................A.....T.....TGG..........................C..................................
XM_009185182.4:  1    A.C.....................A.....T.....TGG..........................C..................................
XM_005576718.3:  1    A.C.....................A.....T.....TGG..........................C..................................
XM_011762253.1:  1    A.C.....................A.....T.....TGG..........................C..................................
XM_017885792.1:  1    A.C.....................A.....T.....TGG..........................C.....T............................
XM_010354645.2:  1    A.C.....................A.....T.....TGG..........................C.....T............................
XM_003943325.3:  1    ..C.....................A...........TGG....................G.....C..................................
XM_001116556.3:  1    ..CA..........................G.....C............................C.....T......................C...G.
XM_008004069.2:  1    ..C.................................C......................G.....C............................CA....
XM_011762371.2:  1    ..T.................................C......................G.....C............................C.....
XM_025358469.1   1    ..C........................................................G.....C...........C................C.....
XM_005576720.3:  1    ..CA................................C............................C.....T......................C...G.
XM_017894043.1:  1    ..C.................................C............................C.C................................
XM_010356490.2:  1    ..C.................................C............................C.C................................
XM_009185183.3:  1    ..T........................................................G.....C............................C.....
XM_012040930.1:  1    ..T...........................................................T..C..................................
XM_028832948.1:  1    ..T...........................................................T..C..................................
XM_015113207.2:  1    ..T.................................C.........................T..C..................................
XM_015113206.2:  1    ..T.................................C.........................T..C..................................
XM_031662204.1:  1    ..T.................................C......................G.....C..................................
XM_031653856.1:  1    ..T.................................C......................G.....C..................................
XM_005576716.3:  1    ..T...........................................................T..C..................................
XM_005576719.3:  1    ..T.................................C.........................T..C..................................
XM_011762251.1:  1    ..T...........................................................T..C..................................
XM_011762255.2:  1    ..T.................................C......................G..T..C..................................
XM_010354836.2:  1    ..C.................................C............................C..................................
XM_017885790.1:  1    ..C.................................C............................C..................................
XM_010354744.2:  1    ..C.................................C............................C..................................
XM_017521001.2:  1    ..C.................................T......................G.....C..................................
XM_012769578.2:  1    ..T....................G......C.....TG...........................C................................G.
XM_012652051.1:  1    ..C....................G......C.....TG...........................C..............................A.G.
XM_012651945.1:  1    ..C....................G......C.....TG...........................C..............................A.G.
XM_003802780.3:  1    ..C....................G............TG..T..................G.....C................................G.
XM_003802781.3:  1    ..C....................G............TG..T..................G.....C................................G.
XM_045558583.1:  1    ..C....................G...........TTG.....................G.....C............G...................G.
XM_045558582.1:  1    ..C....................G............TG.....................G.....C............G...................G.
XM_012769579.2:  1    ..T....................G......C.....TG..............G.G........C................................G.

                         110        120        130        140        150
                 ....|....|....|....|....|....|....|....|....|....|...
NM_006435.3:378  101  GCGACGTGACCGGGGCCCAGGCCTATGCCTCCACCGCCAAGTGCCTGAACATC
NM_021034.3:48-  101  .....................................................
XM_034931794.1:  101  .....................................................
XM_034951329.1:  101  .....................................................
NM_001198767.1:  101  .....................................................
NM_001198757.1:  101  .....................................................
XM_004050342.2:  101  .....................................................
XM_004050337.3:  101  .....A...............................................
XM_002821311.4:  101  .....A................G..............................
XM_009245970.2:  101  .....A................G..............................
XM_003281297.4:  101  .....C...............................................
XM_030801594.1:  101  .....C...............................................
XM_012456047.2:  101  .....C...................C...........................
XM_039471018.1:  101  .....C...................C...........................
XM_003943324.3:  101  .....C...................C...........................
XM_039471016.1:  101  .....C...................C...........................
XM_035263965.1:  101  .....C...................C...........................
XM_035263964.1:  101  .....C...................C.......................T...
NM_003641.5:132  101  .....................................................
XM_034931795.1:  101  .....................................................
NM_001198758.1:  101  .....................................................
XM_004050339.2:  101  .....................................................
NM_001198762.1:  101  ....T.................G..............................
XM_030801595.1:  101  .....C...............................................
XM_035263963.1:  101  .....C...................C...........................
XM_012040931.1:  101  ................T....................................
XM_028832947.1:  96   ..................................T..................
XM_021677005.1:  96   .....C...................C...........................
XM_011929983.1:  101  .....................................................
XM_009185182.4:  101  ................T....................................
XM_005576718.3:  101  ..................................T..................
XM_011762253.1:  101  ..................................T..................
XM_017885792.1:  101  ..........T..........................................
```

```
CD225 domain

XM 011762253.1:  101 ..................................T..................
XM 017885792.1:  101 ..........T..........................................
XM 010354645.2:  101 ..........T..........................................
XM 003943325.3:  101 .....C...................C..................T........
XM 001116556.3:  101 .....C....T..A..........G............................
XM 008004069.2:  101 .....C....T..A.......................................
XM 011762371.2:  101 .....C....T..A..........G............................
XM 025358469.1   101 .T...C....T..A..........G............................
XM 005576720.3:  101 .T...C....T..A..........G............................
XM 017894043.1:  101 .....C....T..A..........G..........A.................
XM 010356490.2:  101 .....C....T..A..........G............................
XM_009185183.3:  101 .....C....T..A..........G............................
XM 012040930.1:  101 .....C....T..........................................
XM 028832948.1:  101 .....C....T..........................................
XM 015113207.2:  101 .....C....T..........................................
XM 015113206.2:  101 .....C....T..........................................
XM 031662204.1:  101 .....C....T........................................G.
XM 031653856.1:  101 .....C....T........................................G.
XM 005576716.3:  101 .....C....T..........................................
XM 005576719.3:  101 .....C....T..........................................
XM 011762251.1:  101 .....C....T..A....................................G..
XM 011762255.2:  101 .....C....T..........................................
XM 010354836.2:  101 .....C....T..........................................
XM 017885790.1:  101 .....C....T..........................................
XM 010354744.2:  101 .....C....T..........................................
XM 017521001.2:  101 .....C...................C...........................
XM 012769578.2:  101 .....C...................C.......................TG..
XM 012652051.1:  101 .....C...................C........................G..
XM 012651945.1:  101 .....C...................C........................G..
XM 003802780.3:  101 .....C..G.T......A..A.............T..................
XM 003802781.3:  101 .....C..G.T......A..A.............T..................
XM 045558583.1:  101 .........................C........................G..
XM 045558582.1:  101 ..................................................G..
XM_012769579.2:  101 .....C.................C.C...........................
```

Figure S7: Alignment CD225 domain of primate IR-IFITMs: Alignment CD225 domain of the AA sequence of primate IR-IFITMs.

C-terminus

```
                                10        20        30        40        50        60        70        80        90       100
                       ....|....|....|....|....|....|....|....|....|....|....|....|....|....|....|....|....|....|....|....|
NM 006435.3:378    1   TGGGCCCTGATTTTGGGCATCTTCATGACCATTCTGCTCATCATCATC---CCAGTGTTG----------------------------------------
XM 034931794.1:    1   .......................................G........---.........----------------------------------------
NM 001198767.1:    1   .......................................G........---.........----------------------------------------
XM 004050342.2:    1   .......................................G........---.........----------------------------------------
XM 009245970.2:    1   ............C........C....................G.....---.........----------------------------------------
XM 034951329.1:    1   ............C........C...................TG.....---.........----------------------------------------
NM 021034.3:48-    1   ............C........C....................G.....---......C..----------------------------------------
NM 001198757.1:    1   ............C........C...................TG.....---.........----------------------------------------
XM 004050337.3:    1   ............C........C....................G.....---.........----------------------------------------
XM 002821311.4:    1   ............C........C....................G.....---.........----------------------------------------
XM 003281297.4:    1   ............C........C..........................---.........----------------------------------------
XM 030801594.1:    1   ............C........C....................G.....---.........----------------------------------------
XM 012456047.2:    1   .....................A...C............G...G.....---...A.AC..----------------------------------------
XM 039471018.1:    1   .....................A..............A.G.........---...A.CC..----------------------------------------
XM 003943324.3:    1   ............C........A..............A.G.........---...A.CC..----------------------------------------
XM 039471016.1:    1   ............C........A......T.......A.G.........---...A.CC..----------------------------------------
XM 035263965.1:    1   ...........C.........C...C............G.........---...A.AC..----------------------------------------
XM 035263964.1:    1   ...........C.........C...C............G.........---..CA.AC..----------------------------------------
XM 012040930.1:    1   .....................C...................TG..G..---......A..----------------------------------------
XM 028832948.1:    1   .....................C............C......TG.....---..G...A..----------------------------------------
XM 015113207.2:    1   .....................C...................TG..G..---......A..----------------------------------------
XM 015113206.2:    1   .....................C...................TG..G..---......A..----------------------------------------
XM 031662204.1:    1   .....................C...................T...G..---......A..----------------------------------------
XM 031653856.1:    1   .....................C...................T...G..---......A..----------------------------------------
XM 005576716.3:    1   .....................C............C......TG.....---..G...A..----------------------------------------
XM 005576719.3:    1   .....................C................G..TG..G..---......A..----------------------------------------
XM 011762251.1:    1   .....................C............C......TG.....---..G...A..----------------------------------------
XM 011762255.2:    1   .....................C...................TG.TG..---......A..----------------------------------------
XM 010354836.2:    1   .....................C...................TG.....---......A..----------------------------------------
XM 017885790.1:    1   .....................C...................TG.....---......A..----------------------------------------
XM 010354744.2:    1   .....................C...................TG.....---......A..----------------------------------------
XM 017521001.2:    1   .....................A................G.........---...A.AC..----------------------------------------
NM 003641.5:132    1   ............C........C...........GGAT.....C.GT.A---.TG..A..CGGCTCTGTGACAGTCTACCATATTATGTTACAGATAATAC
XM 034931795.1:    1   ............C........C...........GGAT.....C.GT.A---.T...A..CGGCTCTGTGACAGTCTACCATATTATGTTACAGATAATAC
NM 001198758.1:    1   ............C........C...........GGAT.....C.GT.A---.T...A..CGGCTCTGTGACAGTCTACCATATTATGTTACAGATAATAC
XM 004050339.2:    1   ............C........C...........GGAT.....C.GT.A---.TG..A..CGGCTCTGTGACAGTCTACCATATTATGTTACAGATAATAC
NM 001198762.1:    1   ............C........C...........GGAT.....C.GT.A---.TG.....TGGCTCTGTGACAGTCTACCATGTTATGTTACAGATAGTAC
XM 030801595.1:    1   ............C........C...........GGAT.....C.GT.G---.TG.....TGGCTCTGTGACAGTCTACCATATTATGTTACAGATAATAC
XM 035263963.1:    1   ...........CG........A...........GGAT.....C.GT.A---.TG.....TGGCTCTGTGATCGTGTACCACGCTGTGTCACAGATCTTAC
XM 003943325.3:    1   ...........CG........A...........GGAT.....C.GC.A---GTG.....TGGCTCTGTGATGATGTACCATGTTGTGTCACGGATCTTAC
XM 021677005.1:    1   .....................A...........GGAT.....C.GT.A---.TG.....TGGCTCTGTGATGCTGTACCATGTTGTGTCACAGATCTTAA
XM 012040931.1:    1   .C..........G........C...........GGAT.....C.GT.A---.TG.....TGGCTCTGTGGCAATCTACCATGTTATGTTACAGATCGTAC
XM 028832947.1:    1   .C..........G........C..........CGGAT.....C.GT.A---.TG....ATGGCTCTGTGGCAATCTACCATGTTATGTTACAGATCGTAC
XM 011929983.1:    1   .C..........G........C..........CGGAT.....C.GT.A---.TG.....TGGCTCTGTGGCAATCTACCATATTATGTTACAGATCGTAC
XM 009185182.4:    1   .C..........G........C...........GGAT.....C.GT.A---.TG.....TGGCTCTGTGGCAATCTACCACGTTATGTTACAGATCGTAC
XM 005576718.3:    1   .C..........G........C..........CGGAT.....C.GT.A---.TG....ATGGCTCTGTGGCAATCTACCATGTTATGTTACAGATCGTAC
XM 011762253.1:    1   .C..........G........C..........CGGAT.....C.GT.A---.TG.....TGGCTCTGTGGCAATCTACCATGTTATGTTACAGATCGTAC
XM 017885792.1:    1   .C..........G........C..........CGGAT.....C.GT.A---.TG.....TGGCTCTGTGGCAATCTACCATATTATGTTACAGATCATAC
XM 010354645.2:    1   .C..........G........C..........CGGAT.....C.GT.A---.TG.....TGGCTCTGTGGCAATCTACCATATTATGTTACAGATCATAC
XM 001116556.3:    1   ..........C.G....T...C.TG....A...GCA....C....C.TTCA.....AG.T----------------------------------------
XM 008004069.2:    1   ..........C.G....T...C.TG....G...GCA....C....C.TTCA.....AG.T----------------------------------------
XM 011762371.2:    1   ..........C.G....T...C.TG....A...GCA....C....C.TTCA.....AG.T----------------------------------------
XM 025358469.1     1   ......G...C.G....T...C.TG....G...GCAG...C....C.TTCA.....AG.T----------------------------------------
XM 005576720.3:    1   ..........C.G....T...C.TG....A...GCA....C....C.TTCA.....AG.T----------------------------------------
XM 017894043.1:    1   ..........C.G........C.TG....G.C.GCA....C....C.TTCA.....AG.T----------------------------------------
XM 010356490.2:    1   ..........C.G..........TG....G.C.GCA....C....C.TTCA.....AG.T----------------------------------------
XM_009185183.3:    1   ..........C.G....T...C.TG....G.C.GCA....C....C.TTCA.....AG.T----------------------------------------
XM 012769578.2:    1   .........G.CC.CA.....C.TGG..........A.....G..GC.---A.G...G.C----------------------------------------
XM 012652051.1:    1   .........G.CC.CA.....C.TGGC.........G.....G.TGC.---A.G...G.C----------------------------------------
XM 012651945.1:    1   .........G.CC.CA.....C.TGGC.........G.....G.TGC.---A.G...G.C----------------------------------------
XM 003802780.3:    1   .........G.CC.CA..C..C.TGG...T...A..............---..G...A.C----------------------------------------
XM 003802781.3:    1   .........G.CC.CA..C..C.TGG...T...A..............---..G...A.C----------------------------------------
XM 045558583.1:    1   .........G.CC.CA.....C.GGG..........G..G..G.....---A.G...G..---------------------------------------G
XM 045558582.1:    1   .........G.CC.CA.....C.GGG..........G..G..G.....---A.G...G..---------------------------------------G
XM_012769579.2:    1   .........G.CC.CA.....C.TGG.T........A.G...G..GG.---ATG...A.C----------------------------------------

                               110       120
                       ....|....|....|....|...
NM 006435.3:378   57   --GTCGTCCAGGCCCAGCGATAG
XM 034931794.1:   57   --.....................
NM 001198767.1:   57   --.....................
XM 004050342.2:   57   --.....................
XM 009245970.2:   57   --A.............T......
XM 034951329.1:   57   --A..T........T.TG.....
NM 021034.3:48-   57   --A..T........T.TG.....
NM 001198757.1:   57   --A..T........T.TG.....
XM 004050337.3:   57   --A..T........T.TG.....
XM 002821311.4:   57   --A..T........T.T.A....
XM 003281297.4:   57   --A..T........T.T.A....
XM 030801594.1:   57   --A.............T......
XM 012456047.2:   57   --A.TC.G..A...T.T.AG...
XM 039471018.1:   57   --A.TC.G..A...T.T..G...
XM 003943324.3:   57   --A.TC.G..A...T.T..G...
XM 039471016.1:   57   --A.TC.G..A...T.T..G...
XM 035263965.1:   57   --A.TC.G..A...T.T.AG...
XM 035263964.1:   57   --A.TC.G..A...T.T.AG...
XM 012040930.1:   57   --A..T....A.....T......
XM 028832948.1:   57   --A..TA...A.....T......
XM 015113207.2:   57   --A..T....A.....T.A....
XM 015113206.2:   57   --A..T....A.....T.A....
XM 031662204.1:   57   --A..T....A.....T......
XM 031653856.1:   57   --A..T....A.....T......
XM 005576716.3:   57   --A..TA...A.....T......
XM 005576719.3:   57   --A..T....A.....T.A....
XM 011762251.1:   57   --A..TA...A.....T......
XM 011762255.2:   57   --A..T....T.....T.A....
XM 010354836.2:   57   --A..TA...A.....T......
XM 017885790.1:   57   --A..TA...A.....T......
XM 010354744.2:   57   --A..TA...A.....T......
XM 017521001.2:   57   --A.TC.G..A...T.T.AG...
NM 003641.5:132   98   AG.AAAAA.G..GTT.C---...
```

```
C-terminus

XM 017521001.2:   57   --A.TC.G..A...T.T.AG...
NM 003641.5:132   98   AG.AAAAA.G..GTT.C---...
XM 034931795.1:   98   AA.AAAAA.G..GTT.C---...
NM 001198758.1:   98   AG.AAAAA.G..GTT.C---...
XM 004050339.2:   98   AG.AAAAA.G..GTT.C---...
NM 001198762.1:   98   AT.AAAAA.G..GTT.C---...
XM 030801595.1:   98   AG.AAAAA.G..GTG.C---...
XM 035263963.1:   98   AGAA.CAAAGA.TT..CTTG...
XM 003943325.3:   98   AG.A.CAAAGAATT.CCTTC...
XM 021677005.1:   98   ACAA.CAAAG..TT..C.TG...
XM 012040931.1:   98   AG.AAAAA...CGTT.C---...
XM 028832947.1:   98   AG.AAAAA...CGTT.C---...
XM 011929983.1:   98   AG.AAAAA...CGTT.C---...
XM 009185182.4:   98   AG.AAAAA...CGTT.C---...
XM 005576718.3:   98   AG.AAAAA...CGTT.C---...
XM 011762253.1:   98   AG.AAAAA...CGTT.C---...
XM 017885792.1:   98   AG.AAAAA...CGTT.C---...
XM 010354645.2:   98   AG.AAAAA...CGTT.C---...
XM 001116556.3:   60   -----------------.TG.GA
XM 008004069.2:   60   -----------------.TG.GA
XM 011762371.2:   60   -----------------.TG.GA
XM 025358469.1    60   -----------------.TG.GA
XM 005576720.3:   60   -----------------.TG.GA
XM 017894043.1:   60   -----------------.TG.GA
XM 010356490.2:   60   -----------------.TG.GA
XM_009185183.3:   60   -----------------.TG.GA
XM 012769578.2:   57   --AG......AAA.TTAACC...
XM 012652051.1:   57   --AG.A...GAAG.TTTTCC...
XM 012651945.1:   57   --AG.A...GAAG.TTTTCC...
XM 003802780.3:   57   --...A.....T.G.GTG.....
XM 003802781.3:   57   --...A.....T.G.GTG.....
XM 045558583.1:   59   TCAG.A.....AG.TTTTCC...
XM 045558582.1:   59   TCAG.A.....AG.TTTTCC...
XM_012769579.2:   57   --AA.TC..GAAA.ATT.AC...
```

Figure S8: Alignment C-terminus of primate IR-IFITMs: Alignment C-terminus of the AA sequence of primate IR-IFITMs.

Supplement: Scattered IFITIMs are retrogenes

TSD (Target site duplication)
Mutation in TSD
Start codon
Premature stop codon in frame
Stop codon IFITM3
Consensus Poly (A) signal
Poly (A)

Genomic Loci of Retro(pseudo)genes

XR_001161573.1 PREDICTED: Otolemur garnettii interferon-induced transmembrane protein 3 pseudogene (LOC100959247), misc_RNA

AGGTGAAACCTTAGACCACCGCAGCCTCCTCTCGGCATCATGAACCACCCTTCCCAAAATATCTTCCCACAAACC
CACCCAGGGCTCCCCCCGAACTATGAGACGCTCAAGGAAGAGCACGAGGTGGCCGTGCTGAGGACGACCCCCAAC
CCTGCTCCCACCGTGATCCACATCCAGAGTGAGACCGCTGTGCCCGACCACGTCGTCTGGTCCCTGTTCAACGCG
CTCTTCATGAACTGCTGCTGCCTGGGCTTCATAGCGTTCGCCTACTCCGTGAAGTCTAGGGATAGGAAGATGGTG
GGCGACCTGGCTGGAGCCAAGACCTACGCCTCCACTGCCAAGTGCCTGAACATCTGGGCCCTGGTCCTCAGCCTC
ATTGGGGCGGCTATTCTGCTCATAGCCATCCCACTGTTCGTCACCCGGTCGCGTGGATAGAGTGGAATCCCTGTC
AAGGCTGCCAGTAACCGCCCATCGCTCCGTGCTCTCAGCTCTGTGCCAGCCCCGCCTGGACCCACCCTTCATGCA
GCTGTTTATACTCACACGCCTGTCCACCAAGGGGTTCAATAAAGTGCACACATTCCTGAAAAAAAAAAAAAGCTG
AAACC

Peptide:
MNHPSQNIFPQTHPGLPPNYETLKEEHEVAVLRTTPNPAPTVIHIQSETAVPDHVVWSLFNALFMNCCCLGFIAF
AYSVKSRDRKMVGDLAGAKTYASTAKCLNIWALVLSLIGAAILLIAIPLFVTRSRG*

XR_504221.2 PREDICTED: Carlito syrichta interferon-induced transmembrane protein 3 pseudogene (LOC103259173), misc_RNA

AAGAAGATAGCCCTCGACCCCTCAGCGCCCCCGTCTCTGGCCATGCACCACACTTCCCAGACCTTCTTCACTCGG
GCCAGCGCCGGCGCCCCCCTGAACTATGAGATGCTCAAGGAGGAGCACGAGGTGGCCATGCTGGGGACGCCCCAC
GGCCCTGCTCCCACGACGTCCACCGTGATCCACATCCACAGTGACACCTCCGTGCCCGACCACGTCGTCTGGTCC
CTGTTCAACACGCTCTTCCTGAATGTCTGCTGTCTGGGCTTCATCGCCTTCGCCTGCTCCGTGAAGTCTAGGGAC
AGGAAGATGGTGGGCGACGTGACCGGGGCCCAGGCCTCCGCCTCCACCGCCAAGTGCCTGAACATCTGGGCCCTG
ATCCTGGGCATCCTTGGGACCATTGGTGTCATCCTCGTTCTGGTCTTGATCTTCGTGGTGTTTCAGTAGACTGTG
AGGCCACGTGGAGTTCATCATCGCCACAGCGAGGCCCATGTGCCCAACCTTCCGTTCTCTTCCCCGTGCCTCGGC
CCCTTCTACAGCAGTTTGTACTCACACACTTTTCTACAGTGGTGGTCAATAAAGTGCATGTGTCACAAAAAAAAA
AAAAAAAAAAAAAAAAAAAAGAAGATAGCCCTC

Peptide:
MHHTSQTFFTRASAGAPLNYEMLKEEHEVAMLGTPHGPAPTTSTVIHIHSDTSVPDHVVWSLFNTLFLNVCCLGF
IAFACSVKSRDRKMVGDVTGAQASASTAKCLNIWALILGILGTIGVILVLVLIFVVFQ*

XM_039468571.1 PREDICTED: Saimiri boliviensis boliviensis interferon-induced transmembrane protein 3-like (LOC104651646), mRNA

AAAAACTTTGATTCTTGAGAAGCATCCCAGCAACCCCACCACAGCAGGTCTTCCCGGACACCATGAACCACACTG
TCCAAACCTTCTTCACTCCCGCCAGCACCGACCGTCCCCCAAACTATGAGATGCTGAAGGAGGAGCATGAGGTGG
CTGCGCTGGGGGTGCCCCACAACCCTGCACCCCCGACATCCACCGTGATCCACATCCGAAGCGAGGCCTCCGTGC
CCGACCATGTCGTCTGGTCCCTGTTCAACACCCTCTTCATGAACTCCTGCTGCCTGGGCTTCATAGCGTTCGCCT
ACTCCGTGAAGTCTAGGGACAGGAAGATGGTTGGCGACCTGACCGGGGCCCAGGCCTACGCCTCCACCGCCAAGT
GCCTGAACATCTGGGCCCTGATTCTGGGCATCATCATGACCATTCTGATGATCATCATCCCAATCCTGATTCTGC
AAGCCTATCGGTAGATACAGAGGAATCACCCGGGCCAGGGGCTCTGCCCGTGACCTGTCTCCCTGGTATCCAGCT
TCCATCCCTCGCCCTGCCCCCAGCCCTTCCTGTATCAACCCTTTACCCTCACACACTTTTCTACAGTGGTTCAAT
AAAGTGCACGTGCTCCTGGAAAAAAAAAAAAAAAAAACTTTGATTCTTG

Peptide:

MNHTVQTFFTPASTDRPPNYEMLKEEHEVAALGVPHNPAPPTSTVIHIRSEASVPDHVVWSLFNTLFMNSCCLGI
AFAYSVKSRDRKMVGDLTGAQAYASTAKCLNIWALILGIIMTILMIIIPILILQAYR*

XM_001112566.4 PREDICTED: Macaca mulatta interferon-induced transmembrane protein 3 (LOC717105), mRNA

TGAAACCCTAATCCTGGAGAACGATCCCACTAACCTGACCACCGCTGGCCTTCGCAGGACACCATGAACCACACG
GTCCAAACCTTCTTCTCTCCTGTCAACAGCGGCCAGCCTCCCAACTATGAGATGCTCAAGGAAGAGCATGAGGTG
GCTATGCTGGGGGCGCCCCACAACCCTGCTCCCCCAACGTCCACCGTGATCCACATCCGCAGCAAAACCTCCGTG
CCCGACCATGTCGTCTGGTCCCTGTTCAACACCCTCTTCATGAACCCCTGCTGCCTGGGCTTCATAGCGTTCGCC
TACTCCGTGAAGTCTAGGGACAGGAAGATGGTTGGCGACCTGACTGGGGCCCAGGCCTATGCCTCCACCGCCAAG
TGCCTGAACATCTGGGCCCTGATTTTGGGCATCCTCATGACCATTCTGCTCATTGTCGTCCCAGTATTGATCTTG
CAAGCCCATCAATAGTTCAGGAGACATCATTCAGGCCAGGAGCTCTGCCCATAACCTGTGTCCCACGTGCTCCAC
CTTCCATTCCTCGCCCTGCCCCCAGAGCCGAGTCCTGTATCAGCCCTTTATCCTCACACACTTTTCTACAATGGC
ATTCAATAAAGTGTACATGTTTCTGGTTAAAAAAAAAAAAAGGAAACCCTAATCCT

Peptide:

MNHTVQTFFSPVNSGQPPNYEMLKEEHEVAMLGAPHNPAPPTSTVIHIRSKTSVPDHVVWSLFNTLFMNPCCLGF
IAFAYSVKSRDRKMVGDLTGAQAYASTAKCLNIWALILGILMTILLIVVPVLILQAHQ*

XR_004026378.1 PREDICTED: Nomascus leucogenys interferon-induced transmembrane protein 3 pseudogene (LOC115830870), misc_RNA

AAAGGATATAATCGAACCCGACCACTGCTGGTCTTCGCTGGACACCATGAACCACACTGTCCAAACCTTCTTCTC
TCCTGTCAACAGCAGCCAGCCCCCCAACTATGAGATGCTCAAGGAGGAGCACGAGGTGGCTGTGCCGGGGGCGCC
CCACAACCCTGCTCCCCCAACGTCCACCATGATCCACATCCGCAGCGAGACCTCTGTGCCCGACCATGTTGTCTG
GTCCCTGTTCAACACCCTCTTCATGAACCCCTGCTGCCTGGGCTTCATAGCATTCGCCTACTCAGTGAAGTCTAG
GGACAGGAAGATGGTTGGCGACCTGACCAGGGCCCAGGCCTATGCCTCCACTGCCAAGTGCCTGAACATCTGGGC
CCTGATTTTGGGCATCCTCATGACCATTCTGCTCATTATCATGCCAGTGTTCATCATCCAAGCCCATCGATAGAT
CAGGAGGCATCATTGAGGCCAGGAGCTCTGCCCATGACCTGTATCCCATGTACTCCACCTTCCATTCCTTGCCCT
GGCCCAGAACCAAATCCTGTATCAGCCCTTTATCCTCACACACTTTTCTACAATGGCATTCAATAAAGTGTATAT
GTTTCTGGTGCTGCTGCAAAAAAAAAAAAAAAAAAAAAGGATATAATC

Peptide:

MNHTVQTFFSPVNSSQPPNYEMLKEEHEVAVPGAPHNPAPPTSTMIHIRSETSVPDHVVWSLFNTLFMNPCCLGF
IAFAYSVKSRDRKMVGDLTRAQAYASTAKCLNIWALILGILMTILLIIMPVFIIQAHR*

XR_002005707.2 PREDICTED: Gorilla gorilla gorilla interferon-induced transmembrane protein 3 pseudogene (LOC109026657), misc_RNA

AGAAATGAAGACCCGGAACCATCCCAGTAACCCGACCACCGCTGGTCTTCGCTGGACACCATGAATCACACTGTC
CAAACCTTCTTCTCTCCTGTCAACAGCGGCCAGCCCCCCAACTATGAGATGCTCAAGGAGGGGCACGAGGTGGCT
GTGCTGGGGGCGCCCCACAACCCTGCTCCTCCGACGTCCACCGTGATCCACATCCGCAGCGAGACCTCCGTGCCC
GACCATGTCGTCTGGTCCCTGTTCAACACCCTCTTCATGAACCCCTGCTGCCTGGGCTTCATAGCATTCGCGTAC
TCCGTGAAGTCTAGGGACAGGAAGATGGTTGGCGACGTGACCGGGGCCCAGGCCTATGCCTCCACCGCCAAGTGC
CTGAACATCTGGGCCCTGATTCTGGGCATCCTCATGACCATTCTGCTCATCGTCATCCCAGTGTTGATCTTCCAG
GCCTACGGATAGATCAGGAGGCATCACTGAGGCCAGGAGCTCTGCCCATGACCTGTATCCCACGTACTCCACCTT
CCATTCCTCGCCCTGCCCCCGGAGCCGAGTCCTGTATCAGCCCTTTATCCTCACACGCTTTTCTACAATGGCATT
CAATAAAGTGCACGTGTTTCTGGTGAAAAAAAAAAAAAAAAAGAAATGAAGACCC

Peptide:

MNHTVQTFFSPVNSGQPPNYEMLKEGHEVAVLGAPHNPAPPTSTVIHIRSETSVPDHVVWSLFNTLFMNPCCLGF
IAFAYSVKSRDRKMVGDVTGAQAYASTAKCLNIWALILGILMTILLIVIPVLIFQAYG*

NG_006210.1 Homo sapiens interferon induced transmembrane protein 9 pseudogene (IFITM9P) on chromosome 11

AAGAATACACTAGAGCGGAGAAAACGAAACTACTAGGGAAAGGAGGGCCCACTGAGAATCACCCCAGAAACCCG
ACCACCACTGGTCTTCGCTGGACACCATGAACCACACTGTCCAAACCTTCTTCTCTCCTGTCAACAGCAGCCAGC
CCCCCCAACTATGAGATGCTCAAGGAGGAACACGACGTGGCTGTGCGGGGGCACCCCACAACCCTGCTCCCCCGA
CATCCACTGTGATCCACATCCGCAGCGAGACCTCCGTGCCCGACCATGTTGTCTGGTCCCTGTTCAACGCCCTCT
TCATGAACCCCTGCCGCCCGGGCTTCAAGGCATTCGCCTACTCCGTGTAGTCTAGGGACAGGAAGATGGTTGGCG

ACCTGACTGGGGCCCAGGCCTATGCCTCCACCGCCAAGTGCCTGAACATCTGGGCCCTGATTTTGGGCATCCTCA
CGACCATTCTGCTCATCGTCATCCCAGTGTTGATCATCCAAGCCCATCGATAGATCAGGAGGCATCATTGAGGCC
AGGAGCTCTGCCCATGACCTGTATCCCACGTACTCCACCTTCCATTCCTCGCCCTGCCCCCGGAGCCAAGTCCTG
TTATCAGCCCTTTATCTTCACACACTTTTCTACAATAGCATTCAATAAAGTGTATATGTTTCTGGTGCTGCTGCG
AAAAAAAAAAAAAAAAAAAAGAATACACTAGAGC

Peptide:
MNHTVQTFFSPVNSSQPPQL*

XR_002477520.1 PREDICTED: Aotus nancymaae interferon-induced transmembrane protein 3 pseudogene (LOC110567062), misc_RNA

AATAAGGTGTAAGTGGGAGAACCATCCCAGCAGGCTGACCACAGCTGGTCTTCCCTGGACACCATGAACCACACT
GTCCAAACCTTCTTCACTCCTGCCAACAGTGGCTGCCCCCCCAAACTATGAGATGCTGAAGGAGGAGCATGAGGT
GGCTGTGCTGGGAGTGCCCCACAACCCTGATCCCCCGACATCCACCGTGATCCACATCCGCAGTGAGACCTCCAT
GCCTGACCATGTCGTCTGGTCCCTGTTCAACACCCTCTTTATGAACTCCTGCTGCCTGGGCTTCATAGCGTTCAC
CTACTCTGTGAAGTCTAGGGACACGAAGATGGTTGGTGACCTGACTGGGGCCCAGGCCTATGCCTCCACCGCTAA
GTGCCTGAACATCTGGTCCCTGATTTAGGCATCATCATGACCATTCTGCTTATCATCATCCCAGAACTCATCTCT
CAAGTCTATCAATAGATCAAGAGAAATCATCCAGGCCAGGAGCTCTGCCTGTGACCTGTTTCCCACATACTCCAC
CTTCCCTTCCTCTCATTGCCCCCGGAGCCGAGTCCTGCATCAATCATTTATCTACACACACTTTTCTACAATGAC
ATTCAATAAAGTGCACGTGTTCCTGGTTAAAAATAATAATAATAAGTTGTAAGTG

Peptide:
MNHTVQTFFTPANSGCPPKL*

XR_748909.2 PREDICTED: Rhinopithecus roxellana interferon-induced transmembrane protein 3 pseudogene (LOC104668909), misc_RNA

TAAAATGCAGTACCACTAACCCGACCACCGCTGGCCTTCGCAGGACACCATGAACCACACGGTCCAAACCGTCTT
CTCTCCTGTCAATAGCGGCCAGCCTCCCAGCTATGAGATGCTGAAGGAAGAGCACGAGGTGGCTGTGCTGGGGGC
TCCCCACAACCCTGCTCCCCCGATGTCCACCGTAATCCACATCCGCAGCGAGACCTCCGTGCCCGACCATGTCGT
CTGGTCCCTGTTCAACACCCTCTTCATGAACCCCTGCTGCCTGGGCTTCATAGCATTCGCCTACTCCGTGAAGTC
TAGGGACAGGAAGATGGTTGGCGACCTGACTGGGGCCCAGGCCTATGCCTCCACCGCCAAGTGCCTGAACATCTG
GGCCCTGATTTTGGGCATCCTCATGACCATTCTGCTCATTGTCATCCCAGTATTGATCTACCAAGCCCATCGATA
GATCAGGAGACATCATTCAGGCCAGCAGCTCCGCCCACAACCTGTGTCCCACGTGCTCCACCTTCCTTCCATTCC
TCGCCCTGCCCCCGGAGCGAGTCCTGTATCAGCCCTTTATCTTCACACTTTTCTACAATGGCATTCAATAAAGTG
TACATGTTTCTGGTGCTGCTGCGAAAAAAAAAAATAAAATAAAATAAAAATAAAATAAAATAAAATGCAGTACC

Peptide:
MNHTVQTVFSPVNSGQPPSYEMLKEEHEVAVLGAPHNPAPPMSTVIHIRSETSVPDHVVWSLFNTLFMNPCCLGF
IAFAYSVKSRDRKMVGDLTGAQAYASTAKCLNIWALILGILMTILLIVIPVLIYQAHR*

XR_001017992.1 PREDICTED: Cercocebus atys interferon-induced transmembrane protein 3 pseudogene (LOC105594389), misc_RNA

AAAAAGTATCTTTAAAGGAAAAATCGAAACTACTAGGGAAAGGGAGGGCCCACTGAGAATCATCCCACTAACCCG
ACCACTGCTGGCCTTCGCAAGACACCATGAACCGCACGGTCCAAACCGTCTTCTCTCCTGTCAACAGCGGCCAGC
CCCCCAGCTATGAGATGCTCAAGGAAGAGCACAAGGTGGCTGTGCTGGGGGCGCCCCACAACCCTGCTCCCCTGA
CGTCCACCGTGATCCACATCCGCAGCGAGACCTCCGTGCCCGACCATGTTGTCTGGTCCCTGTTCAACACCCTCT
TCATGAACCCCTGCTGCCTGGGCTTCATAGCATTCACCTACTCCGTGAAGTCTAGGGACAGGAAGATAGTTGGCG
ACCTGATTGGGGCCCAGGCCTATGCCTCCACCGCCAAGTGCCTGAACATCTGGGCCCTGATTTTGGGCATCCTCA
TGACCATTCTGCTCATTGTCATCCCAGTATTGATCTACCAAGCCCATCGATAGATCAGGAGACATCATTCAGGCC
AGGAGCTCTGCCTATAACCTGTATCCCACGTGCTCCACCTTCCATTCCTGGTCCTGCCCCCGGAGCCGAGTCCTG
TATCAGCCCTTTATCCTCACACACTTTTCTACAATGGCATTCAATAAAGTATACATGTTTCTGGTTACAAAAAAA
AAAAAAAAGTATCTTTAAAG

Peptide:
MNRTVQTVFSPVNSGQPPSYEMLKEEHKVAVLGAPHNPAPLTSTVIHIRSETSVPDHVVWSLFNTLFMNPCCLGF
IAFTYSVKSRDRKIVGDLIGAQAYASTAKCLNIWALILGILMTILLIVIPVLIYQAHR*

>XR_002913425.1 PREDICTED: Pongo abelii interferon-induced transmembrane protein 3 pseudogene (LOC100453335), misc_RNA

AAAGGATATAATCGAACCCGACCACCGCTGGTCTTCGCTGGACACCATGAACCACGCTGTCCAAACCTTCTTCTC
TCCTGTCAACAGCGGCCAGCCCCTCAACTATGAGATGCTCAAGGAGGAGCATGAGGTGGCTGTGCCAGGGGTGCC
CCACAGCCCTGCTCCCCCGACATCCACTGTGATCCACATCTGCAGCGAGACCTCCGTGCCCGACCATGTTGTCTT
GTCCCTGTTCAACACCTTCATGAACCCCTGCTGCCTGGGCTTCATAGCATTCACCTACTCCGTGAAGTCTAGGGA
CAGGAAGATGGTTGGCGACCTGACCGGGGCCCACGCCTATGCCTCCACCGCCAAGTGCCTGAACATCTGGGCCCT
GATTTTGGGCATCCTCATGACCATTCTGCTCATTGTCATCCCAGTGTTGATCATCCAAGCCCATCGATAGATCAG
GAGGCATCACTGAGGCCAGGAGCTCTGCCCATGACCTGTATCCCATGTACTCCACCTTCCACTCCTTGCCCTGCC
CCAGAGCCAAGTCCTGTATCAGCCCTTTATCCTCACATACTTTTTTACAATGGCAGTCAATAAAGTGTATATGTT
TCTGGTGCTGCTGCAAAAAAAAAAAAAAAAAAAAAAAAAGGATA

Peptide:
MNHAVQTFFSPVNSGQPLNYEMLKEEHEVAVPGVPHSPAPPTSTVIHICSETSVPDHVVLSLFNTFMNPCCLGFI
AFTYSVKSRDRKMVGDLTGAHAYASTAKCLNIWALILGILMTILLIVIPVLIIQAHR*

>XR_001716631.2 PREDICTED: Pan troglodytes interferon-induced transmembrane protein 3 pseudogene (LOC101057405), misc_RNA

AAATATACAATATTAGGGCCCACTGAGAGCCATCCCAGTAACCCGACCACCGCTGGTCTTCACTGGACCGCCATG
AACCACACTGTCCAACCTTCTTCTCTCCTATCAACAGCGGCCAGCCCCCCAACTATGAGATGCTCAAGGACGAGC
ACAAGGTGGCTGTGCTGGGGGCGGCCTACAACCCTGCTCCTCCGACGTCCACCGTGATCCACATCCGCAAAGAGA
CCTCTGTGCCCGACCATGTTGTCTGGTCCCTGTTCAACACTCTCTTCATGAACCCCTGCTGCCTGGGCTTCATAG
CATTCGCTTACTCCGTGAAGTCTAGGCACAGGAAGATGGTTGGCGACCTGACTGGCGCCCAGGCCTATGCCTCCA
CCGCCAAGTGCCTGAACATCTGGGCCCTGATTTTGGGCATCCTCATGACCATTCTGCTCATCATCATCCCAGTGT
TGATCTTCCAAGTCTATCAATAGATCAGGAGGCATCATTGAGGCCAGGAGCTCTGCCCATGACCTGTATCCCATG
TACTCCACCTTCCATTCCTCGCCCTGCCCCAGAGCCGAGTCCTGTGTCAGCCCTTTATCCTCACACACTTTTCTA
CAACGGCATTCAATAAAGTGCACGTGTTTCTGGTAATAAATAAATAAATAAATAAAAATATACA

Peptide:
MNHTVQPSSLLSTAASPPTMRCSRTSTRWLCWGRPTTLLLRRPP*

>XM_034961680.1 PREDICTED: Pan paniscus interferon-induced transmembrane protein 3-like (LOC100993091), mRNA

AAAAATGTACAGTCCAGGGCCCACTCAGAACCATCCCAGCAACCTGACCACAGCTGGTCTTTGCTGGACACCATG
AACCACACTGCCCAAACCTTCTTCATTCCTGCCAACAGCGGCTGCCCTCCCCCGCCCCCCAACCGCAGCTATGAG
ATGCTCAAGGAGGAGCATGAGGTGGCTGTGCTGGGGGCACCCCACAACCCTGCTCCGCCGATGTCCACCATGATC
CATATCTGCAGCGAGACCTCCGTGTCTGACTATGTTGTCTGGTCCCTGTCCAACATCCTCTTCATGAACCCCCAC
TGCCTGGGATTCATAGCATTCACCTACTCCCTGAAGTCTAGGGACAGGAAGATGGTTGGAGACCTGACTGGGGCC
CAGGCCTATGCCTCCACTGCCAAGTACCTGAACATCTGAGCCCTGATTGTGTGCATCATCATGACCATTCTGCTC
ACCATCATCATCCTAGTGTTGATCTTCCAAGTCTGTCGATAGATCAGGAGGCATCATCCAGGCCAGGAGCTCTGC
CCATGACCTGTATTCCACATACTCCAACTTCCATTCCTCGTCCTGGCCCCAGAGCTGAGTTCTGTATCATCCCTT
TATCCTCACACACTTTTCTACAATGGCATTCAATAAAGTGCATGTGTTCCTGGTTAAAAAAAAAATGTAC

Peptide:
MNHTAQTFFIPANSGCPPPPPNRSYEMLKEEHEVAVLGAPHNPAPPMSTMIHICSETSVSDYVVWSLSNILFMNP
HCLGFIAFTYSLKSRDRKMVGDLTGAQAYASTAKYLNI*

>NG_006230.2 Homo sapiens IFITM3P pseudogene 6 (IFITM3P6) on chromosome 12

ATAAAAATCTTTTTTTTTTTTTGGAGAACCATCCCAGCAACCCAACCACAGCTGGTTCTCCCTGGAGACCATGAA
CCACACTGTCCAAACCCTCTTCACTCCTGCCAACACCGGCCGCTCCACCAACCATGAGATGCTCAAGGAGAAGCA
TGAGGTGGCTGTGCTGGGGGCACCCCACAACCCTGTGCCTCCAGCGTTCACCATGATCCACATCTGCAGTGAGAC
CTCCGTGCCCGACCATGTCGTCTGGTCCCTATTCAACACCCTCTTCAAGAATTCCTGCTGCCCGGACTTCATAGC

ATTCATCTACTCTGTGAAGTCTAGGACAGGAAGTCTATGGACAGGAAGATGGTTGGTGACCTGACTGGGGCCCAG
GCCTGTGTCTCCACTGCCAAGTGCCTGAACATCTGGGCCCTGGCTCTGGGCATCCTCCTGACCATTCTGCTCATC
ATCATCTCAGTGCTGATCTTCCAAGTCTCTCGATAGAACAGGAGACAGCATCCGGGCCAGGAGCTCTGCCCACAA
CCTGTCTCCCATGCACTCCGCCTTCCATTCCTTGCCCTGCCCCCAAAGCTGAGTCCTGTATCAGCCCTTTATCCT
CACACACTTTTCTACAATGTCATTCAATAAAGTGCATGTGTTCGTGGTGCAAAAAAATAAAAAT

Peptide:
MNHTVQTLFTPANTGRSTNHEMLKEKHEVAVLGAPHNPVPPAFTMIHICSETSVPDHVVW
SLFNTLFKNSCCPDFIAFIYSVKSRTGSLWTGRWLVT*

>XM_034966156.1 PREDICTED: Pan paniscus interferon-induced transmembrane protein 3-like (LOC100985648), mRNA

AATAGTTCAGGAGAAATTGAAACTACTGGGGAGAGGGAGGGCCTACTGGGAACCATCCCAGCAACCCGACCACAG
CTGGTCTTCCCTGGACACCATGAACCACACTTTCCAAACCTTCTTCACTCCTGCCAACAGCGGCCACCCCCGCAA
TTATGAGATGCTCAAGGAGGAGCATGAGGTGGCTGTGGTGGGGGCGCCCCAAAACCCTGCTCTCCCGATGTCCAC
CATGATCCACTTCCACAGTGAGACCTCCATGCCTGACCATGTCGTGTGGTCGCTGTTCAACACCCTCTTCATGAA
CTCCGGCTGCCTGGGCTTCATAGCATTTGCGTACTCCTTGAAGTCTAGGGAGAGGAAGAAGGTTGGTGACCTGAC
CAGGGCCCAGGCCTATACCTCCACTGCCAAGTGCCTGAACATCTGGGCCCTGATCGTGGACATTGTCATGACCAT
TCTGCTCATCATCATCCCAGTGTTGATCTTCCAAGTCTATTGATAGATCAGGAGGCATCATCCAGGCCAGGAGCT
CTGCCCATGACCTGTATCCCACGCACTCCATCTTTCATTCCTTGCACTGCCCCCAGAGCCAAGTCCTGTATCAGC
CCTTTATCCTCACACACTTTTCTACAATGGCATTCAATAAAGTGCACGTGCGCCTGGTTAAAAAAAAAAAAAAAGT
TCAGG

Peptide:
MNHTFQTFFTPANSGHPRNYEMLKEEHEVAVVGAPQNPALPMSTMIHFHSETSMPDHVVWSLFNTLFMNSGCLGF
IAFAYSLKSRERKKVGDLTRAQAYTSTAKCLNIWALIVDIVMTILLIIIPVLIFQVY*

>XR_169790.4 PREDICTED: Pan troglodytes interferon-induced transmembrane protein 3 pseudogene (LOC101057658), misc_RNA

AAAGGATATAATCGAACCCGACCACCGCTGGTCTTCGCTGGACACCATGAACCACACTGTCCAAACCTTCTTCTC
TCCTGTCAACAGCGGCCAGCCCCTCAACTATGAGATGCTCAAGGAGGAGCATGAGGTGGCTGTGCCAGGGGTGCC
CCACAACCCTGCTCCCCCGACGTGATCCACATCTGCAGCGAGACCTCCGTGCCCAACCATGTTGTCTGGTCCCTG
TTCAACACCCTCTTCATGAACCCCTGCTGCCTGGGCTTCATAGCATTCACCTACTCCATGAAGTCTAGGGACAGG
AAGATGGTTGGCGACCTGACCGGGGCCCAGGCCTATGCCTCCACCGCCAAGTGCCTGAACATCTGGGCCCTGACT
TTGGGCATCCTCATGACCATTCTGCTCATCGTCATCCCAGTGTTGATCATCCAAGCCCATTGATAGATCAGGAGG
CATCACTGAGGCCAGGAGCTCTGCCCATGACCTGTATCCCATGTACTCCACCTTCCACTCCTTGCCCTGCCCCGG
AGCCAAGTCCTGTATCAGCCCTTTATACTCACACACTTTTCTACAATGGCATTCAATAAAGTGTATATGTTTCTG
GTGCTGCTGCGAAAAAAAAAAAGGACATAATC

Peptide:
MNHTVQTFFSPVNSGQPLNYEMLKEEHEVAVPGVPHNPAPPT*

>XM_004052942.3 PREDICTED: Gorilla gorilla gorilla putative dispanin subfamily A member 2d (LOC101143671), mRNA

TAATTTCAAATATGGCGAAAACGAAACTACTGGGGAAACGGAGGGTCCGCTGAGAATCATCCCAGTAACCCAACC
ACGGCTGGTCTTCGCTGGACACCATGAACCACACTGTCCAAACCTTCTCTCCTGTCAACAGCGGCCAGCCCCCCA
ACTATGAGATGCTCAAGGAGGAGCACAAGGTGGCTGTGCTGGGGGTGCCCCACAACCCTGCTCCCCCGACGTCCA
CCGTGATCCACATCCGCAGCAAGACCTCCGTGCCCCACCATGTCGTCTGGTCCCTGTTCAATACCCTCTTCATGA
ACTCCTGCTGCCTGGGCTTCATAGCATTCGCCTACTCCGTGAAGTCTAGGGACAGGAAGATGGTTGGCAACGTGA
CCGGGGCCCAGGCCTATTCCTCCACCGCCAAGTGCCTGAACATCTGGGCCCTGATTTTGGGCATCCTCATGACCA
TTCTGCTCATCGTCATCCCAGTATTAATCTTCCAAGCCCATCGATAGATCAGGAGACATCATTGAGGCCAGGAGA
CCTACCCATGACCTGTATCCTTTGTACTCCGTACTCCACCTTCCATTCCTCGCCCTGCTCCCGGAGCCAAGTCCT
GTATCAGCCCTTTATCCTCACACACTTTTTTACAATGGCATTCAATAAAGTGTATGTGTTTCTGGTAAAATAAAT
AAATAAATTCAAATATG

Peptide:

MNHTVQTFSPVNSGQPPNYEMLKEEHKVAVLGVPHNPAPPTSTVIHIRSKTSVPHHVVWS
LFNTLFMNSCCLGFIAFAYSVKSRDRKMVGNVTGAQAYSSTAKCLNIWALILGILMTILL
IVIPVLIFQAHR*

>XR_656019.2 PREDICTED: Pongo abelii interferon-induced transmembrane protein 3 pseudogene (LOC100443671), misc_RNA

AAGAATACACTAGAGCGGAGGAAACGAAACTACTAGGGAAAGGGAGGGCCCACTGACAGCCATCCCAGTAACCCG
ACCACCACTGGTCTTCGCTGGACACCATGAACCACACTGTCCAAACCTTCTCTCCTGTCAACAGCGGCCAGCCCC
CCAGCTATGAGATGCTCAAGGAGGAGCACGAGGTGGCTGTGCCGGGGGCGCCCCACAACCCTGCTCCCCCGACGT
CCACTGTGATCCATATCCGCAGTGAGACCTCCGTGCCCAACCATGTTGTCTGGTCCCTGTTCAACACCCTCTTCA
TGAACCCCTGCCGCCTAGGCTTCATAGCATTCGCCTACTCTGTGAAGTCTAGGGACAGGAAGATGGTTGGCAACC
TGACCGAGGCCCAGGCCTATGCCTCCACCGCCAAGTGCCTGAACATCTGGGCCCTGATTTTGGGCATCCTCATGA
CCATTCTGCTCATCGTCATCCCAGTGTTGATCATCCAAGCCCATCGATAGATCAGGAGGCATCACTGAGGCCAGA
AGCTCTGCCCATGACCTGTATCCCACGTCCTCCACCTTCCATTCCTCGCCCTGCCCCCGAAGCCAAGTCCTGTAT
CAGCCGTTTATCCTCACACACTTTTCTACAATGGCATTCAATAAAGTGTATATGTTTCTGGTGCTGCTGCGAAAA
AAAAAAAAAAGAATACACTAGAGC

Peptide:

MNHTVQTFSPVNSGQPPSYEMLKEEHEVAVPGAPHNPAPPTSTVIHIRSETSVPNHVVWS
LFNTLFMNPCRLGFIAFAYSVKSRDRKMVGNLTEAQAYASTAKCLNIWALILGILMTILL
IVIPVLIIQAHR*

>XR_004027821.1 PREDICTED: Nomascus leucogenys interferon-induced transmembrane protein 3 pseudogene (LOC100585336), misc_RNA

ATTATAGGTAGTGACCCATTAATACTGAGTGCAGAGAACCATCCCCGTAACTCGACCACCGCTGGTCTTCGCTGG
ACACCATGAACCACACTGTCCAAACCTTCTTCTCTCCTGTCAACAGCGGCCAGCCCCTCAATTATGAGATGCTCA
AGGAGGAGCACGAGGTGGCTGTGCTGGGGGCACCCCACAACCCTGCTTCCCCGATGCCCACCGTGGTCCACATCC
GCAGCAAGACCTCCGTGCCCGACCATGTCGTCTGGTCCCTGTTCAACACCCTCTTCATGAACCCCTGCTGCCTGG
GCTTCATAGCATTCACCTACTCCGTGAAGTCTAGGGACAGGAAGATGGTTGGTGACGTGACCGGGGCCCAGGCCT
ATGCCTCCACTGCCAAGTGCCTGAACATCTGGGCCCTGATTCTGGGCATCCTCATGACCATTCTGCTCATCGTCA
TCCCAGTATTGATCTTCCAGGCCTATCGATAGATCAGGAGGCATCATTGAGGCCAGGAGCTCTGCCCATGACCTG
TATCCCACATATCCACCTTTCATTGCTCACCCTGCCCCCGGAGCCGAGTCCTGTATCAGCCCTTTATCCTCACAC
ACTTTTCTACAATGGCATTCAATAAAGTGCACGTGTTTCTGGTAAAAAAAAAAAAAAAAAAAAAAATATAG

Peptide:

MNHTVQTFFSPVNSGQPLNYEMLKEEHEVAVLGAPHNPASPMPTVVHIRSKTSVPDHVVW
SLFNTLFMNPCCLGFIAFTYSVKSRDRKMVGDVTGAQAYASTAKCLNIWALILGILMTIL
LIVIPVLIFQAYR*

>XR_001438791.2 PREDICTED: Macaca mulatta interferon-induced transmembrane protein 3 pseudogene (LOC706375), misc_RNA

AGAAGGAAAGGGAAACAATGCAGCTGACCTGACTGAGAACCATCCCACTAACCCGACCACTGCTGGCCTTTGCAG
GACACCATGAACCATACAGTCCAAACCTTCTTCTCTCCTATCAACAGTGGCCAGCCACCCCACTATGAGACGCTC
AAGGAGAAGCACGAGGTGGCTGTGCTGAGGGTGCCCCACAACACTGCTCCCCCAACGTCCACCGTGATCCACATC
TGCAGCGAGACCTCCGTGCCTGATCATGTCGTCTGGTCCCTGTTCAACACCCTCTTCATGCTCTTCATGAACCCC
TGCTGCCTGGGCTTCATAGCATTCTCCTACTCTGTGAAGTCTAGGGACAGGAAGATGGCTGGTGACTTGACTGGG
GCCTAGGCCTATGCTTCCACCGCTAAGTGCCTGAACATCTGGGCCCTGATTTTGGGCATCCTCATGACCATTCTG
CTCATCGTCATCCCAGTATTGATCTTGCAAGCCTATTGATAGATCAGGAGACATCATTCAGGCCAGGAGCTCTGC
CCATAACCTGTATCCCACGTGCTCCACCTTCCATTCCTCGCCCTGCCCCCGGAGCTGAGTCCCGTATCAGCCCTT
TATCCTCACACACTTTTCTACAATGGCATTCAATAAAGTGTATATGTTTCTGAAAAAAAAAAAAAAAAAAAGAA
GTAAA

Peptide:

MNHTVQTFFSPINSGQPPHYETLKEKHEVAVLRVPHNTAPPTSTVIHICSETSVPDHVVW
SLFNTLFMLFMNPCCLGFIAFSYSVKSRDRKMAGDLTGA*

>XR_001011714.1 PREDICTED: Cercocebus atys interferon-induced transmembrane protein 3 pseudogene (LOC105576564), misc_RNA

GAAATCACAGAGCTTGGAGAACCATCCCACTAACCCGACCACCGCTGGCCTTCGCAGGACACCATGAACCACACA
GTCCAAACCTTCATCTCTCCTGTCAGCAGCGGCCAGCCCCCGAACTATGAGATTCTCAAGGAAGAGCACGAGGTG
GCTATGCTGGGGGCGCCCCACAACCCTGCTCCCCCGACGTCCACCGTGATCCACATCCGCAGCGAGACCTCCGTG
CCCAACCATGTCGTCTGGTCCCTGTTCAACACCCTCTTCATGAACCCCTGCTGCCTGGGCTTCATAGCGTTTGCC
TACTCCATGAAGTCTAGGGACAGGAAGATGGTTGGCGACCTGACTGGGGCCCAGGCCTATGCCTCCACCGCCAAG
TGCCTGAACATCTGGGCCCTGATTTTGGGCATCCTCATGACCATTCTGCTCATTGTCGTCCCAGTATTGATCTCC
CAAGCCCATCAATAGGTCAGGAGACATCATTCAGGCCAGGAGCTCTGCCCATAACCTGTGTCCCACGTGCTCCAC
CTTCCATTCCTCGCCCTGCCCCCAGAGCTGAGTTCTGTATCAGCCCTTTATCCTCACACACTTTTCTACAATGGC
ATTCAATAAAGTGTACATGTTTCTGGTGCTAAAAAAAAAAAAAAAAAAAAAGAAATCACAGAGCTTG

Peptide:
MNHTVQTFISPVSSGQPPNYEILKEEHEVAMLGAPHNPAPPTSTVIHIRSETSVPNHVVW
SLFNTLFMNPCCLGFIAFAYSMKSRDRKMVGDLTGAQAYASTAKCLNIWALILGILMTIL
LIVVPVLISQAHQ*

>XM_030922644.1 PREDICTED: Rhinopithecus roxellana interferon-induced transmembrane protein 3 (LOC115894740), mRNA

AGAAAAGTACCAGTCTTGACTAACCCGACCACCGCTGGCCTTCGCAGGACACCATGAACCACACGGTCCAAACCG
TCTTCTCTCCTGTCAATAGCGGCCAGCCTCCCAACTATGAGTTGCTCAAGGAGGAGCAGGAGGTGGCTGTGCTGG
GGGCGCCCCACAACCCTGCTCCCCCGACGTCCACCGTGATCCACATCCGCAGCGAGACCTCCGTGCCCGACCATG
TCGTCTGGTCCCTGTTCAACACCCTCTTCATGAACCCCTGCTGCCTGGGCTTCATAGCATTCGCCTACTCCGTGA
AGTCTAGGGACAGGAAGATGGTTGGTGACCTGACTGGGGCCCAGGCCTATGCCTCCACCGCCAAGTGCCTGAACA
TCTGGGCCCTGATTTTGGGCATCCTCATGACCATTCTGCTCATTGTCATCCCAGTATTGATCTACCAAGCCCATC
GATAGATCAGGAGACATCATTCAGGCCAGCAGCTCCGCCCATAACCTGTGTCCCACGTGCTCCACCTTCCTTCCA
TTCCTCGCCCTGCCCCCGGAGCGAGTCCTGTATCAGCCCTTTATCCTCACACTTTTCTACAATGGCATTCAATAA
AGTGTACATGTTTCTGGTGCTGCTGCGACTTCAAAAAAAAAAAAAAAAAAAAAGAAAAGTACCAGTCTTG

Peptide:
MNHTVQTVFSPVNSGQPPNYELLKEEQEVAVLGAPHNPAPPTSTVIHIRSETSVPDHVVW
SLFNTLFMNPCCLGFIAFAYSVKSRDRKMVGDLTGAQAYASTAKCLNIWALILGILMTIL
LIVIPVLIYQAHR*

>XR_001106643.2 PREDICTED: Aotus nancymaae interferon-induced transmembrane protein 3 pseudogene (LOC105713798), misc_RNA

TTAAAAACAAATTGGAGAGATCCAAACTATGGTGGAAAGGGAGAGCTCACTGAGAACCATCCCAGCAACCTGACC
ACAGCTGGTCTTCCATGGACACCTTGAACCACACTGTCCAAACCTTCTTCACTCCTGCCAACACTGGCCATCCTC
CTAACTATAAGTTGATCAAGAAAGAGCATGAGGTGACTGTGCTGGGGGTGCCCCACAACCCTGCTCCCCCGACAT
CCACCGTGATCCACATCTGCAGCGAGACCTCCGTGCCCGACCATGTCATCTGGTCCCTGTTCAACACCCTCTTCA
TGAACTCCTGCTGCCTGGGCTTCATAGTGGTTGCCTACTCCGTGAAGTCTAGGAACAGGCAGATGGTTGGCGAAC
TGTTGGGGCCCAGGCCTATACCTCCACCGCCAAGTGCCTGAACTTCTGGGCCCTGATTTTGGGCATCATCATGAC
CATTATGCTCATCATTATCCCCGTGTTGATCCACCACGTCTATCGATAGAGCAGGAGGCAGCCTCCAGGCCAGGA
GCTCTGCCCATGACCTGTATCTCACGTACTCCATCTTCCATTCCTCGCCCTGCCCCCGGAACTGAGTTCTGTATC
AGCCCTTTATCCTCACACACTTTTCTACAATGGCATTCAATAAAGTGCACGTGTTCCTGGAAAAAAAATTTAAAA
A

Peptide:
MDTLNHTVQTFFTPANTGHPPNYKLIKKEHEVTVLGVPHNPAPPTSTVIHICSETSVPDH
VIWSLFNTLFMNSCCLGFIVVAYSVKSRNRQMVGELLGPRPIPPPPSA*

>XM_039478903.1 PREDICTED: Saimiri boliviensis boliviensis interferon-induced transmembrane protein 3-like (LOC120367594), mRNA

TCTAGGGTCACTGCAACCTCTGCCTTCTAGGTTCAAGCGATTCTTCTGCCTCGGCCTCCCAAGTAGCTGGGATTA
CAGGCACCTGCCACCAAAAAAAAAAAAAAAAAAAAAAAAAAAAAAAAAAAAAAAAAAAACAACTCTTTAAAATATTTAA
CATGGAGAAGCATCCCAGTAACCCCACCATGGTCGGTCTTCCCGGACACCATGAACCACACTGTCCAAACCTTCT
TTACTCCTGCCAGCACCGACCGTCCCCCAAACTATGAGATGCTGAAGGAGGAGCATGAGGTGGCTGCGCTGGGGG
TGCCCCACAACACTGCACCCCCGACATCCACCGTGATCCACATCCGAAGCGAGACCTCCATGCCTGACCATGTCG
TCTGGTCCCTGCTCAACACCCTCTTCATGAACTCCTGCTGCCTGGGCTTCATAGTGTTCGCCTACTCCGTGAAGT
CTAGGGACAGGAAGATGGTTGGCGACCTGACCGGGGCTCAGGCCTACGCCTCCACTGCCAAGTGCCTGAACATCT
GGGCCCTGATTTTGGGCATCATCATGACCATTCTGATGATGATCATCCCAATCCTGATTCTGCAAGCCTATCAGT
AGATCCAGAGGAATCACCTGGGCCAGGGGCTCTGCCCCGTGACCTGTCTGTCTCCCTGGTATCCAGATTCCATCC
CTCGCCCTGCCCCCAGTCCATTCTGTATCAACCCTTTACCCTCACACACTTTTCTACAGTGGTTCAATAAAGTGC
ACGTGCTCCTGGAAAAAAAAAAAACAAAAACAAAAAAATTAACATATTTGAGATCAGCCTGGACAACATCATGAAA
CCCCATCTCTGCAAAAAAATACAAATAAAATAAACCTCTTTAAAATAGGTTTATTGTTGTCATAAATACAGGCAT
GAGCCACTGTGCCTGGCCCAGAGATTCTTCAATTTGCAATTAGTTAAAGGAATAAGGCTCTGCCTAAAACTGGGA
GCTGACAGAAAGCAATGCTTAAGTTAA

Peptide:
MNHTVQTFFTPASTDRPPNYEMLKEEHEVAALGVPHNTAPPTSTVIHIRSETSMPDHVVW
SLLNTLFMNSCCLGFIVFAYSVKSRDRKMVGDLTGAQAYASTAKCLNIWALILGIIMTIL
MMIIPILILQAYQ*

>XM_008052862.1 PREDICTED: Carlito syrichta interferon-induced transmembrane protein 3-like (LOC103254874), mRNA

GGCACTGAGTCATGCTGTTTTTGGTCAAAAATTGTTGAATACCCAGAGAGATGTGGGGAGATGTGCTCGTGAATC
ACCCATCATGAAATGGGTAAACGTGTTAAAAGAATCTCAAAAAAAAAAAAAAATTCACTGGAGGGGAAGGATCCC
CTGAGCATCTTCCTCTCATCTGGACCGACAGCGGTTCCTGTCCCCACTCCATGAACCACGCTTCCCAGACCTTCA
CTTGGGCCAGAGCCGGCGCTGCCTCAAACTATGAGATGCTCAAGGAGGAGCACGAAGGGGACGTGCTGGGGACAT
CCCACAGCTCTGCTCTCATGACGTCCACCGTGATCCACGTCCACAGCGACCCCTCCGTGCCCGACCACGTCGTCT
GGTCCCTGTTCAACACGCTCTTCCTGAACGTCTGCAGTCTGGGCTTCATCGCCTTCGCCTACTCCGTGAAGGTGC
GTGTGGGCCCCTGTGGGAGTCCAGGAAGGCGCCTCTGAACTGTGGCCAGCTTGGCCAGCTCCACCGACTCAGGCT
AGGAACAGGAAGATGGTGGGCGACGTGACCAGGGCCCAGGCCTACGCCTCCACCACCAAGTGCCTGAACATCTGG
GCCCTGATCCTCCGCATCCTTGGGACCATTGGCGTCATCCTTGTTCCAGTCTTGATCTTCCTAGTGTTTCAGTAG
ACTGTGAGGCCACTTGGAGTTCCTCATCGCCATGGCCAGGCCCATGTGCCCGACCTTCCATTCTCTTCCCCCTGC
CTCCGCCCCTTATACAGCAGTTTGTACTCACACTTTTCTACAGTGGCAGTCAATAAAGTGCAAGTGTTGAGAAAA
AAAAAATTCACTGGAGACAAATGCAGGCTCTCACAACAACGCTAGCTGGTACACTGATGCCAATGGGTTCCTAGA
ACACTTACCTAGCAGATAAAGCCTGTACTATATGGGGCCTGCCCTCCATAAAATAATTCTGGGTTTTGGGGGGTT
CCCCTTCACTATGTCCAGACAATTACCTCACAGGAAGGAAGACAAATGTGTGACCACTGGATTTGGTCACACAGA
GGTTAATGGTGAGCTTGAAAGTGGT

Insert? Marked

Peptide: with insert
MNHASQTFTWARAGAASNYEMLKEEHEGDVLGTSHSSALMTSTVIHVHSDPSVPDHVVWS
LFNTLFLNVCSLGFIAFAYSVKVRVGPCGSPGRRL*

Peptide: spliced
MNHASQTFTWARAGAASNYEMLKEEHEGDVLGTSHSSALMTSTVIHVHSDPSVPDHVVWS
LFNTLFLNVCSLGFIAFAYSVKARNRKMVGDVTRAQAYASTTKCLNIWALILRILGTIGV
ILVPVLIFLVFQ*

Figure S9: Scattered IFITMs possess features of retrogenes: Genomic sequence of the IFITMs 200 bp upstream of the canonical start codon and 400 bp downstream of the canonical stop codon were manually searched for canonical start codon, canonical stop codon, premature stop codon, poly A signal (AATAAA), poly A start and target-site duplications (5' and 3' UTR).

```
                                                   10        20        30        40        50        60        70        80
NM_021034.3 Homo sapiens inter      1    AACCCGACCGCCGCTGGTCTTCGCTGGACA----------CCATGAATCACACTGTCCAAACCTTCTTCTCTCCTGTCAA
NG_006210.1 Homo sapiens inter      1    .........A..A.................----------.......C................................
NG_006230.2 Homo sapiens IFITM      1    .....A...A.A......TC..C.....G.----------.......C..................C.....A......C...
XM_034951329.1 PREDICTED: Pan       1    .........A....................----------........................................
XM_034961680.1 PREDICTED: Pan       1    ....T....A.A.........T........----------.......C.......C.................AT.....C...
XM_034966156.1 PREDICTED: Pan       1    .........A.A..........C.......----------.......C......T..................A......C...
NM_001198757.1 Pan troglodytes     1    .........A....................----------........................................
XR_001716631.2 PREDICTED: Pan       1    .........A............A......CG---------.......C............-..............A....
XR_169790.4 PREDICTED: Pan tro      1    .........A....................----------.......C................................
XM_004050337.3 PREDICTED: Gori      1    .........A....................----------........................................
XR_002005707.2 PREDICTED: Gori      1    .........A....................----------........................................
XM_004052942.3 PREDICTED: Gori      1    .....A...A.G..................----------.......C.......................---..........
XM_009245970.2 PREDICTED: Pong      1    .........A....................----------.......C.....................---............
XR_002913425.1 PREDICTED: Pong      1    .........A....................----------.......C...G............................
XR_656019.2 PREDICTED: Pongo a      1    .........A..A.................----------.......C...................CTC..---.....
XM_030801594.1 PREDICTED: Noma      1    .........A.................G..----------.......C................................
XR_004026378.1 PREDICTED: Noma      1    .........A.T..................----------.......C................................
XR_004027821.1 PREDICTED: Noma      1    ...T.....A....................----------.......C................................
XM_015113206.2 PREDICTED: Maca      1    .........A.......C....A.A.....----------.......C.....G..........................
XM_001112566.4 PREDICTED: Maca      1    ....T....A.......C......A.....----------.......C.....G..........................
XR_001438791.2 PREDICTED: Maca      1    .........A.T.....C...T..A.....----------.......C..T..A.....................A....
XM_012040930.1 PREDICTED: Cerc      1    .........A.TA....C......C.....----------.......C.....G.........G................
XR_001017992.1 PREDICTED: Cerc      1    .........A.T.....C......AA....----------.......C.G...G.........G................
XR_001011714.1 PREDICTED: Cerc      1    .........A.......C......A.....----------.......C.....A............A............G
XM_010354836.2 PREDICTED: Rhin      1    .........A.......C......A.....----------.......C.....G.........G................
XR_748909.2 PREDICTED: Rhinopi      1    .........A.......C......A.....----------.......C.....G.........G................
XM_030922644.1 PREDICTED: Rhin      1    .........A.......C......A.....----------.......C.....G.........G................
XM_039471018.1 PREDICTED: Saim      1    .....C...A.G..G.......T-......----------.......C.......................A....C.C..G
XM_039468571.1 PREDICTED: Saim      1    .....C...A.A..A.......C-......----------.......C.......................A....C.C..G
XM_039478903.1 PREDICTED: Saim      1    T.A..CCA.CAT.G.C.GTC.TC.C.....----------.......C......................TA......C..G
XM_012456047.2 PREDICTED: Aotu      1    .......T.A.A..CT..........G..----------.......C........G..............A....C.C..G
XR_002477520.1 PREDICTED: Aotu      1    .GG.T....A.A..........C.......----------.......C.......................A......C...
XR_001106643.2 PREDICTED: Aotu      1    .T...-.G.AA.-...AC.ACA.....T.TTCCATGGACA..T....C.......................A......C...
XM_008053679.1 PREDICTED: Carl      1    C.GA.CC.GCAGCGCCCC.G..T.CA.-------------.....C.C......TC...G.........A...GG.C..G
XR_504221.2 PREDICTED: Carlito      1    TCGA.CC.TCAGCGCCCC.G..T....-------------.....C.C......TC...G.........A...GG.C..G
XM_003802780.3 PREDICTED: Otol      1    CT.AGAC.ACTGCAGCC..C..T.-..-..----------.......C...T..TC.....GTG.....A...GAAC.C.
XR_001161573.1 PREDICTED: Otol      1    CTTAGAC.AC.GCAGCC..C..T.-..-..----------T......C...C..TC.....ATA.....C.A.AAAC.C.

                                                   90       100       110       120       130       140       150       160
NM_021034.3 Homo sapiens inter     71    CAGTGGCCAGCCCCCC---------------AACTATGAGATGCTCAAGGAGGAGCACGAGGTGGCTGTGCTGGGGGCGC
NG_006210.1 Homo sapiens inter     71    ...CA...........C--------------.......................A.....C..........-......A.
NG_006230.2 Homo sapiens IFITM     71    ..CC....-..T..A.C--------------...C..................A....T.....................A.
XM_034951329.1 PREDICTED: Pan      71    ...C............---------------.................................................
XM_034961680.1 PREDICTED: Pan      71    ...C...TGC..T...CCGCCCCCCAACCGC.G........................T.....................A.
XM_034966156.1 PREDICTED: Pan      71    ...C.....C....G.---------------..T.......................T............G.........
NM_001198757.1 Pan troglodytes    71    ...C............---------------.................................................
XR_001716631.2 PREDICTED: Pan      71    ...C............---------------....................C......A.....................G
XR_169790.4 PREDICTED: Pan tro     71    ...C..........T.---------------..........................T.............CA....T..
XM_004050337.3 PREDICTED: Gori     71    ...C............---------------.................................................
XR_002005707.2 PREDICTED: Gori     71    ...C............---------------......................G..........................
XM_004052942.3 PREDICTED: Gori     68    ...C............---------------...........................A..................T..
XM_009245970.2 PREDICTED: Pong     68    ...CA...........---------------.....C..............................A............
XR_002913425.1 PREDICTED: Pong     71    ...C..........T.---------------..........................T.............CA....T..
XR_656019.2 PREDICTED: Pongo a     68    ...C............---------------.G......................................C........
XM_030801594.1 PREDICTED: Noma     71    ...C............---------------.........G.........................C.............
XR_004026378.1 PREDICTED: Noma     71    ...CA...........---------------........................................C........
XR_004027821.1 PREDICTED: Noma     71    ...C..........T.---------------..T.............................................A.
XM_015113206.2 PREDICTED: Maca     71    ...C........T...---------------....................A........T......A..A.........
XM_001112566.4 PREDICTED: Maca     71    ...C........T...---------------....................A.....T.........A............
XR_001438791.2 PREDICTED: Maca     71    ............A...C---------------.........C..........A....................A...T..
XM_012040930.1 PREDICTED: Cerc     71    ...C............---------------.G...C..............A...........A................
XR_001017992.1 PREDICTED: Cerc     71    ...C............---------------.G..................A......A.....................
XR_001011714.1 PREDICTED: Cerc     71    ...C...........G---------------...........T........A...............A............
XM_010354836.2 PREDICTED: Rhin     71    T..C........T...---------------.G............G.....A...........................T.
XR_748909.2 PREDICTED: Rhinopi     71    T..C........T...---------------.G............G.....A...........................T.
XM_030922644.1 PREDICTED: Rhin     71    T..C........T...---------------.........T................G......................
XM_039471018.1 PREDICTED: Saim     70    ..CC.A..GT.....A---------------..............G...........T.......A...........T..
XM_039468571.1 PREDICTED: Saim     70    ..CC.A..GT.....A---------------..............G...........T..........C........T..
XM_039478903.1 PREDICTED: Saim     71    ..CC.A..GT.....A---------------..............G...........T..........C........T..
XM_012456047.2 PREDICTED: Aotu     71    ..CCCA...T.....G---------------.....C........G........A..T........C.C...........
XR_002477520.1 PREDICTED: Aotu     71    .......TGC......A--------------..............G...........T.................A.T..
XR_001106643.2 PREDICTED: Aotu     79    ..C......T..T..T---------------......A..T..A.....A.A.....T......A............T..
XM_008053679.1 PREDICTED: Carl     68    .GCC...GCC.....G---------------...................................C.........A...
XR_504221.2 PREDICTED: Carlito     68    .GCC...GCC....TG---------------...................................CA........A...
XM_003802780.3 PREDICTED: Otol     69    ..CA..G.TC.....G---------------.........CA.........A........A.....C........TT...
XR_001161573.1 PREDICTED: Otol     69    .CCA..G.TC.....G---------------..........C.........A..............C......A..A..A

                                                  170       180       190       200       210       220       230       240
NM_021034.3 Homo sapiens inter    136    CCCACAACCCTGCTCCCCCGACGTCCACCGTGATCCACATCCGCAGCGAGACCTCCGTGCCCGACCATGTCGTCTGGTCC
NG_006210.1 Homo sapiens inter    136    ......................A.....T.........................................T.........
NG_006230.2 Homo sapiens IFITM    136    ............TG..T..AG...T....A...........T....T.................................
XM_034951329.1 PREDICTED: Pan     136    ................................................................................
XM_034961680.1 PREDICTED: Pan     151    ................G....T.......A.......T...T.................T.T...T....T.........
XM_034966156.1 PREDICTED: Pan     136    ....A..........T.....T.......A........T...A...T.........A....T...........G.....G
NM_001198757.1 Pan troglodytes   136    .....................T.........................T.................................
XR_001716631.2 PREDICTED: Pan     136    ..T.............T............................AA........T..............T.........
XR_169790.4 PREDICTED: Pan tro    136    ........................-------..........T....................A.......T.........
XM_004050337.3 PREDICTED: Gori    136    ................................................................................
XR_002005707.2 PREDICTED: Gori    136    ................T...............................................................
XM_004052942.3 PREDICTED: Gori    133    ...............................................A..............C.................
XM_009245970.2 PREDICTED: Pong    133    ......................A...............................................T.........
XR_002913425.1 PREDICTED: Pong    136    ......G...............A.....T............T............................T....T....
XR_656019.2 PREDICTED: Pongo a    133    ............................T........T........T...............A.......T.........
XM_030801594.1 PREDICTED: Noma    136    ..............G.................................................................
XR_004026378.1 PREDICTED: Noma    136    ...................A.........A.........................T..............T.........
XR_004027821.1 PREDICTED: Noma    136    ..............T......TC.........G..............A................................
XM_015113206.2 PREDICTED: Maca    136    .....................................T.......................T........T.........
XM_001112566.4 PREDICTED: Maca    136    ...................A...........................A.A..............................
XR_001438791.2 PREDICTED: Maca    136    ........A..........A.....................T...................T..T...............
```

```
XM_012040930.1 PREDICTED: Cerc  136 ........T.........................................T...................T.........
XR_001017992.1 PREDICTED: Cerc  136 ..................T...................................................T.........
XR_001011714.1 PREDICTED: Cerc  136 ..............................................................A.................
XM_010354836.2 PREDICTED: Rhin  136 .....................T.........A................................................
XR_748909.2 PREDICTED: Rhinopi  136 .....................T.........A................................................
XM_030922644.1 PREDICTED: Rhin  136 ................................................................................
XM_039471018.1 PREDICTED: Saim  135 .............A........A...........T........A....................................
XM_039468571.1 PREDICTED: Saim  135 .............A........A....................A......G.............................
XM_039478903.1 PREDICTED: Saim  136 ........A....A........A....................A............A....T..................
XM_012456047.2 PREDICTED: Aotu  136 .............G........A............................................C............
XR_002477520.1 PREDICTED: Aotu  137 ............A.........A.......................T.........A....T..................
XR_001106643.2 PREDICTED: Aotu  144 ......................A..................T.............................A........
XM_008053679.1 PREDICTED: Carl  133 .....GG...C.T....A....................G...A......C.................C............
XR_504221.2 PREDICTED: Carlito  133 .....GG..........A........................A...T..C.................C............
XM_003802780.3 PREDICTED: Otol  134 ...C.............A.---------.............ATG.T......G.T...........C............
XR_001161573.1 PREDICTED: Otol  134 ...C.............A.---------.............AG..T......G.T...........C............

                                             250       260       270       280       290       300       310       320
                                    ....|....|....|....|....|....|....|....|....|....|....|....|....|....|....|....|
NM_021034.3 Homo sapiens inter   216 CTGTTCAACACCCTCTTCAT---------GAACCCCTGCTGCCTGGGCTTCATAGCATTCGCCTACTCCGTGAAGTCTAG
NG_006210.1 Homo sapiens inter   216 .........G..........---------..........C...C........AG..................T.......
NG_006230.2 Homo sapiens IFITM   216 ..A................A---------...TT.........C..A.............AT......T...........
XM_034951329.1 PREDICTED: Pan   216 T...................---------...................................................
XM_034961680.1 PREDICTED: Pan   231 ....C.....T.........---------.......CA.........A............A........C..........
XM_034966156.1 PREDICTED: Pan   216 ....................---------....T..G......................T..G......T..........
NM_001198757.1 Pan troglodytes  216 ....................---------...................................................
XR_001716631.2 PREDICTED: Pan   216 ...........T........---------.................................T.................
XR_169790.4 PREDICTED: Pan tro   209 ....................---------...............................A........A..........
XM_004050337.3 PREDICTED: Gori  216 ....................---------.................................G.................
XR_002005707.2 PREDICTED: Gori  216 ....................---------.................................G.................
XM_004052942.3 PREDICTED: Gori  213 ........T...........---------....T..............................................
XM_009245970.2 PREDICTED: Pong  213 ....................---------.......................................T...........
XR_002913425.1 PREDICTED: Pong  216 ............---.....---------...............................A...................
XR_656019.2 PREDICTED: Pongo a  213 ....................---------..........C....A.......................T...........
XM_030801594.1 PREDICTED: Noma  216 ....................---------...................................................
XR_004026378.1 PREDICTED: Noma  216 ....................---------.......................................A...........
XR_004027821.1 PREDICTED: Noma  216 ....................---------...............................A...................
XM_015113206.2 PREDICTED: Maca  216 ....................---------..............................T....................
XM_001112566.4 PREDICTED: Maca  216 ....................---------...........................G.......................
XR_001438791.2 PREDICTED: Maca  216 ....................GCTCTTCAT...............................T.......T...........
XM_012040930.1 PREDICTED: Cerc  216 ....................---------....A.........................T....................
XR_001017992.1 PREDICTED: Cerc  216 ....................---------...............................A...................
XR_001011714.1 PREDICTED: Cerc  216 ....................---------...........................G..T.........A..........
XM_010354836.2 PREDICTED: Rhin  216 ....................---------...................................................
XR_748909.2 PREDICTED: Rhinopi  216 ....................---------...................................................
XM_030922644.1 PREDICTED: Rhin  216 ....................---------...................................................
XM_039471018.1 PREDICTED: Saim  215 ............A.......---------....T......................G.......................
XM_039468571.1 PREDICTED: Saim  215 ....................---------....T......................G.......................
XM_039478903.1 PREDICTED: Saim  216 ...C................---------....T.....................TG.......................
XM_012456047.2 PREDICTED: Aotu  216 ....................---------....T......................G.......................
XR_002477520.1 PREDICTED: Aotu  217 .................T..---------....T......................G...A.......T...........
XR_001106643.2 PREDICTED: Aotu  224 ....................---------....T.....................TGG.T....................
XM_008053679.1 PREDICTED: Carl  213 ...........G......C.---------....GT......T...........C..C.......................
XR_504221.2 PREDICTED: Carlito  213 ...........G......C.---------...TGT......T...........C..C.......G...............
XM_003802780.3 PREDICTED: Otol  205 ...........G........---------....TG..T..................G.......................
XR_001161573.1 PREDICTED: Otol  205 .........G.G........---------....TG.....................G.......................

                                             330       340       350       360       370       380       390       400
                                    ....|....|....|....|....|....|....|....|....|....|....|....|....|....|....|....|
NM_021034.3 Homo sapiens inter   287 GGACAGGAAGATGGTTGGCGA----------------CGTGACCGGGGCCCAGGCCTATGCCTCCACCGCCAAGTGCCTG
NG_006210.1 Homo sapiens inter   287 .....................----------------.C....T....................................
NG_006230.2 Homo sapiens IFITM   287 .-........-.CTA...AC.GGAAGATGGTTGGTGA.C....T.............G..T......T............
XM_034951329.1 PREDICTED: Pan   287 .....................----------------...........................................
XM_034961680.1 PREDICTED: Pan   302 ..................A..----------------.C....T.......................T.......A....
XM_034966156.1 PREDICTED: Pan   287 ...G.......A......T..----------------.C.....A..............A.......T............
NM_001198757.1 Pan troglodytes  287 .....................----------------...........................................
XR_001716631.2 PREDICTED: Pan   287 .C...................----------------.C....T..C.................................
XR_169790.4 PREDICTED: Pan tro   280 .....................----------------.C.........................................
XM_004050337.3 PREDICTED: Gori  287 .....................----------------.A.........................................
XR_002005707.2 PREDICTED: Gori  287 .....................----------------...........................................
XM_004052942.3 PREDICTED: Gori  284 ...................A.----------------.....................T....................
XM_009245970.2 PREDICTED: Pong  284 .....................----------------.A................G........................
XR_002913425.1 PREDICTED: Pong  284 .....................----------------.C.............C..........................
XR_656019.2 PREDICTED: Pongo a  284 ...................A.----------------.C......A..................................
XM_030801594.1 PREDICTED: Noma  287 .....................----------------.C.........................................
XR_004026378.1 PREDICTED: Noma  287 .....................----------------.C.....A......................T............
XR_004027821.1 PREDICTED: Noma  287 ..................T..----------------.............................T............
XM_015113206.2 PREDICTED: Maca  287 .....................----------------.C....T....................................
XM_001112566.4 PREDICTED: Maca  287 .....................----------------.C....T....................................
XR_001438791.2 PREDICTED: Maca  296 ..............C...T..----------------.T....T......T..........T........T.........
XM_012040930.1 PREDICTED: Cerc  287 .....................----------------.C....T....................................
XR_001017992.1 PREDICTED: Cerc  287 ............A........----------------.C...TT....................................
XR_001011714.1 PREDICTED: Cerc  287 .....................----------------.C....T....................................
XM_010354836.2 PREDICTED: Rhin  287 .....................----------------.C....T....................................
XR_748909.2 PREDICTED: Rhinopi  287 .....................----------------.C....T....................................
XM_030922644.1 PREDICTED: Rhin  287 ..................T..----------------.C....T....................................
XM_039471018.1 PREDICTED: Saim  286 .....................----------------.C...................C.....................
XM_039468571.1 PREDICTED: Saim  286 .....................----------------.C...................C.....................
XM_039478903.1 PREDICTED: Saim  287 .....................----------------.C..........T........C........T............
XM_012456047.2 PREDICTED: Aotu  287 ...............C.....----------------.C...................C.....................
XR_002477520.1 PREDICTED: Aotu  288 .....C............T..----------------.C....T..........................T.........
XR_001106643.2 PREDICTED: Aotu  295 .A.....C.............----------------AC..-TT...............A....................
XM_008053679.1 PREDICTED: Carl  284 ...............G.....----------------................---------------------------
XR_504221.2 PREDICTED: Carlito  284 ...............G.....----------------....................CC.....................
XM_003802780.3 PREDICTED: Otol  276 ...............G.....----------------.C..G.T......A..A.............T............
XR_001161573.1 PREDICTED: Otol  276 ...T...........G.....----------------.C..G.T..A...A..A....C........T............

                                             410       420       430       440       450       460       470       480
                                    ....|....|....|....|....|....|....|....|....|....|....|....|....|....|....|....|
NM_021034.3 Homo sapiens inter   351 AACATCTGGGCCCTGATTCTGGGCATCCTCATGACCATTCTGCTCATCGTCAT---CCCAGTGCTGATCTTCCAGGCCTA
NG_006210.1 Homo sapiens inter   351 ..................T............C.....................---.......T.....A....A...C.
NG_006230.2 Homo sapiens IFITM   365 ...............GC.............C.................A....---.T................A.T..C
XM_034951329.1 PREDICTED: Pan   351 ...............................................T.....---.......T................
```

```
XM_034961680.1 PREDICTED: Pan    366 ........A.........G..T.....A..................C.A....CAT..T....T..........A.T..G
XM_034966156.1 PREDICTED: Pan    351 .................CG...A...TG....................A....---.......T..........A.T...
NM_001198757.1 Pan troglodytes   351 ...............................................T.....---.......T................
XR_001716631.2 PREDICTED: Pan    351 ..................T.............................A....---.......T..........A.T...
XR_169790.4 PREDICTED: Pan tro   344 ................C.T..................................---.......T.....A....A...C.
XM_004050337.3 PREDICTED: Gori   351 .....................................................---.......T................
XR_002005707.2 PREDICTED: Gori   351 .....................................................---.......T................
XM_004052942.3 PREDICTED: Gori   348 ..................T..................................---......AT.A........A...C.
XM_009245970.2 PREDICTED: Pong   348 .....................................................---.......T.....G........C.
XR_002913425.1 PREDICTED: Pong   348 ..................T............................T.....---.......T.....A....A...C.
XR_656019.2 PREDICTED: Pongo a   348 ..................T..................................---.......T.....A....A...C.
XM_030801594.1 PREDICTED: Noma   351 .....................................................---.......T.....G........C.
XR_004026378.1 PREDICTED: Noma   351 ..................T............................TA....---G......T.C...A....A...C.
XR_004027821.1 PREDICTED: Noma   351 .....................................................---......AT................
XM_015113206.2 PREDICTED: Maca   351 ..................T............................T...G.---......AT..........A...C.
XM_001112566.4 PREDICTED: Maca   351 ..................T............................T...G.---......AT.......G..A...C.
XR_001438791.2 PREDICTED: Maca   360 ..................T..................................---......AT.......G..A.....
XM_012040930.1 PREDICTED: Cerc   351 ..................T............................T...G.---......AT..........A...C.
XR_001017992.1 PREDICTED: Cerc   351 ..................T............................T.....---......AT......A...A...C.
XR_001011714.1 PREDICTED: Cerc   351 ..................T............................T...G.---......AT......C...A...C.
XM_010354836.2 PREDICTED: Rhin   351 ..................T............................T.....---......AT......A...A...C.
XR_748909.2 PREDICTED: Rhinopi   351 ..................T............................T.....---......AT......A...A...C.
XM_030922644.1 PREDICTED: Rhin   351 ..................T............................T.....---......AT......A...A...C.
XM_039471018.1 PREDICTED: Saim   350 ..................T........A..............A.G...A....---....A.C.....TC.G..A.....
XM_039468571.1 PREDICTED: Saim   350 ...........................A..............A.G...A....---....A.C.....TC.G..A.....
XM_039478903.1 PREDICTED: Saim   351 ..................T........A..............A.G..GA....---....A.C.....TC.G..A.....
XM_012456047.2 PREDICTED: Aotu   351 ..................T........A...C............G........---....A.A.....TC.G..A.....
XR_002477520.1 PREDICTED: Aotu   352 .........T........T-A......A................T...A....---.....AA..C....CT..A.T...
XR_001106643.2 PREDICTED: Aotu   358 ...T..............T........A...........A........A.T..---...C...T.....CA...C.T...
XM_008053679.1 PREDICTED: Carl   320 --------------------------------------------------------------------------------
XR_504221.2 PREDICTED: Carlito   348 .................C...........TGG.......GGTG.....C..G.---T.TG..CT........GT..TG.T
XM_003802780.3 PREDICTED: Otol   340 ...............G.C..CA..C....TGG...T...A........A....---...G...A.CG..A......TGCG
XR_001161573.1 PREDICTED: Otol   340 ...............G.C..CA..C..A.TGG.G.GGC.A.T..GC..A.AGCCAT....C..T.CG..AC..G.T.GCG

                                              490       500       510       520       530       540       550       560
                                     ....|....|....|....|....|....|....|....|....|....|....|....|....|....|....|....|
NM_021034.3 Homo sapiens inter   428 TGGATAGATCAGGAGGCATCACTGAGGCCAGGAGCTCTGCCC-ATGACCTGTA----TCCCACGTACTCC-------AAC
NG_006210.1 Homo sapiens inter   428 .C...................T....................-..........----.............-------.C.
NG_006230.2 Homo sapiens IFITM   442 .C......A......A..G..TCCG.................-.CA......C----.....T.C.....-------GC.
XM_034951329.1 PREDICTED: Pan    428 .........................................T-..........----.............-------.C.
XM_034961680.1 PREDICTED: Pan    446 .C...................TCC..................-..........T---...-..A......-------...
XM_034966156.1 PREDICTED: Pan    428 .T...................TCC..................-..........----.......C.....-------.T.
NM_001198757.1 Pan troglodytes   428 .........................................T-..........----.............-------.C.
XR_001716631.2 PREDICTED: Pan    428 .CA..................T....................-..........----.....T.......-------.C.
XR_169790.4 PREDICTED: Pan tro   421 .T........................................-..........----.....T.......-------.C.
XM_004050337.3 PREDICTED: Gori   428 ..........................................-..........----...........T.-------.C.
XR_002005707.2 PREDICTED: Gori   428 C.........................................-..........----.............-------.C.
XM_004052942.3 PREDICTED: Gori   425 .C.............A.....T............AC..A...-..........----...TTT.......GTACTCC.C.
XM_009245970.2 PREDICTED: Pong   425 .C...................T....................-..........----......A......-------.C.
XR_002913425.1 PREDICTED: Pong   425 .C........................................-..........----.....T.......-------.C.
XR_656019.2 PREDICTED: Pongo a   425 .C.............................A..........-..........----........C....-------.C.
XM_030801594.1 PREDICTED: Noma   428 .C...................T....................-..........----A.....A......-------.C.
XR_004026378.1 PREDICTED: Noma   428 .C...................T....................-..........----.....T.......-------.C.
XR_004027821.1 PREDICTED: Noma   428 .C...................T....................-..........----......A..TC.A--------C.
XM_015113206.2 PREDICTED: Maca   428 .CA....G.......A.....T.C..................-..A......G----........G....-------.C.
XM_001112566.4 PREDICTED: Maca   428 .CA....T.......A.....T.C..................-..A......G----........G....-------.C.
XR_001438791.2 PREDICTED: Maca   437 .T.............A.....T.C..................-..A.......----........G....-------.C.
XM_012040930.1 PREDICTED: Cerc   428 .C.............A.G...T.C..................-..A.......----......A.G....-------.C.
XR_001017992.1 PREDICTED: Cerc   428 .C.............A.....T.C.................T-..A.......----........G....-------.C.
XR_001011714.1 PREDICTED: Cerc   428 .CA....G.......A.....T.C..................-..A......G----........G....-------.C.
XM_010354836.2 PREDICTED: Rhin   428 .C.............A.....T.C.......C.....C....-.CA......G----........G....-------.C.
XR_748909.2 PREDICTED: Rhinopi   428 .C.............A.....T.C.......C.....C....-.CA......G----........G....-------.C.
XM_030922644.1 PREDICTED: Rhin   428 .C.............A.....T.C.......C.....C....-..A......G----........G....-------.C.
XM_039471018.1 PREDICTED: Saim   427 .C.G......CA....A.....CCG......AG.........-G........CTGTC....TG..-....-------.G.
XM_039468571.1 PREDICTED: Saim   427 .C.G.....ACA....A.....CCG.......G.........-G........C----....TG..-....-------.G.
XM_039478903.1 PREDICTED: Saim   428 .CAG......CA....A.....CTG.......G.........CG........CTGTC....TG..-....-------.GA
XM_012456047.2 PREDICTED: Aotu   428 .CAG.....GCA....A.....CCC.......G.........-G........C----.............-------.G.
XR_002477520.1 PREDICTED: Aotu   428 .CA.........A...AA....TCC................T-G........T----......A......-------.C.
XR_001106643.2 PREDICTED: Aotu   435 .C......G.........G.CTCC..................-..........----..T..........-------.T.
XM_008053679.1 PREDICTED: Carl   320 --------------------------------------------------------------------------------
XR_504221.2 PREDICTED: Carlito   425 .CAG....CTGT.....CA.GTG...TT..TC.T.---...A-CA-----.CGAG--G....T..G.C.A-------.C.
XM_003802780.3 PREDICTED: Otol   417 ........GTG.A.TC.C.GT.A-....---------....A-G....T--------G....TCAC.C.A-------TG.
XR_001161573.1 PREDICTED: Otol   420 ........GTG.A.TC.C.GT.A-....---------....A-G.A...--------G....TCGCTC.G-------TG.

                                              570       580       590       600       610       620       630       640
                                     ....|....|....|....|....|....|....|....|....|....|....|....|....|....|....|....|
NM_021034.3 Homo sapiens inter   496 TTCCATTCCTCGCCC-----TGCCCCCGGAGCC-GAGTCCTGT------ATCAGCCCTTTATCCTCACACGCTTTTCTAC
NG_006210.1 Homo sapiens inter   496 ...............-----.............-A.......T-----..............T......A.........
NG_006230.2 Homo sapiens IFITM   510 ..........T....-----.......AA...T-.........------...............................A.........
XM_034951329.1 PREDICTED: Pan    496 ...............-----.............-.........------...............................
XM_034961680.1 PREDICTED: Pan    514 ............T..-----..G....A....T-....T....------....T................A.........
XM_034966156.1 PREDICTED: Pan    496 ..T.......T..A.-----.......A.....-A........------.....................A.........
NM_001198757.1 Pan troglodytes   496 ...............-----.............-.........------...............................
XR_001716631.2 PREDICTED: Pan    496 ...............-----.......A.....-.........------G.....................A.........
XR_169790.4 PREDICTED: Pan tro   489 .....C....T....-----......-......-A........------.............A.......A.........
XM_004050337.3 PREDICTED: Gori   496 ...............-----.............-.........ACCTGT...............................
XR_002005707.2 PREDICTED: Gori   496 ...............-----.............-.........------...............................
XM_004052942.3 PREDICTED: Gori   500 ...............-----...T.........-A........------.....................A.....T...
XM_009245970.2 PREDICTED: Pong   493 ........T......-----.............-.C.......------.....................A.........
XR_002913425.1 PREDICTED: Pong   493 .....C....T....-----......-A.....AA-.......------....................TA.....T...
XR_656019.2 PREDICTED: Pongo a   493 ...............-----........A....AA-.......------.......G.............A.........
XM_030801594.1 PREDICTED: Noma   496 .A.............-----...G...A.....-.........------..G..................A.........
XR_004026378.1 PREDICTED: Noma   496 ..........T....-----..G...-A..A..AA.-......------.....................A.........
XR_004027821.1 PREDICTED: Noma   495 ..T....G...A...-----.............-.........------.....................A.........
XM_015113206.2 PREDICTED: Maca   496 ...............-----.......A.....-.........------.....................A.........
XM_001112566.4 PREDICTED: Maca   496 ...............-----.......A.....-.........------.....................A.........
XR_001438791.2 PREDICTED: Maca   505 ...............-----............T-......C..------.....................A.........
XM_012040930.1 PREDICTED: Cerc   496 ..........G.T..-----.......A....T-.........------................T....A.........
XR_001017992.1 PREDICTED: Cerc   496 ..........G.T..-----.............-.........------.....................A.........
XR_001011714.1 PREDICTED: Cerc   496 ...............-----.......A....T-....T....------.....................A.........
XM_010354836.2 PREDICTED: Rhin   496 ....T.C.A.TC.T.-GCCC............--.........------..............T......--........
XR_748909.2 PREDICTED: Rhinopi   496 ....-....ATT..TCGCCC............--.........------..............T......--........
XM_030922644.1 PREDICTED: Rhin   496 ....T.C.A.TC.T.-GCCC............--.........------..............T......--........
```

```
XM_039471018.1 PREDICTED: Saim  498 ..------------------------------------------------------------------------------
XM_039468571.1 PREDICTED: Saim  494 ......C........-----.......A.CC.----T......------....A.......C........A.........
XM_039478903.1 PREDICTED: Saim  500 ......C........-----.......A.TC.---.T..-...------....A.......C........A.........
XM_012456047.2 PREDICTED: Aotu  496 ....G......A...-----......G.CC.AG----......------............C..............G...
XR_002477520.1 PREDICTED: Aotu  496 ....C......T.AT-----.............-........C------....AT.A......TA.....A.........
XR_001106643.2 PREDICTED: Aotu  503 ...............-----..........A.T-....T....------.....................A.........
XM_008053679.1 PREDICTED: Carl  320 --------------------------------------------------------------------------------
XR_504221.2 PREDICTED: Carlito  487 ....G...TCTT...CG---....T.G.CCC.-----.T..A---------....AG...G.A.......A.........
XM_003802780.3 PREDICTED: Otol  471 .CT.TGCT..GTG..AGCCC.C..T-G.ACC..ACCC.T.ATG--------....TG.....A.......A.C.A..C..
XR_001161573.1 PREDICTED: Otol  474 .CT..GCT..GTG..AGCCCC...T-G.ACC.-ACCC.T.ATG--------....TG.....A.........C.G..C..

                                            650       660       670
                                    ....|....|....|....|....|....|....|
NM_021034.3 Homo sapiens inter  564 AATGGCATT--CAATAAAGTGCACGTGTTTCTGGT
NG_006210.1 Homo sapiens inter  565 ...A.....--..........T.TA..........
NG_006230.2 Homo sapiens IFITM  578 ....T....--............T....CG.....
XM_034951329.1 PREDICTED: Pan   564 .........--........................
XM_034961680.1 PREDICTED: Pan   582 .........--............T.....C.....
XM_034966156.1 PREDICTED: Pan   564 .........--................CGC.....
NM_001198757.1 Pan troglodytes  564 .........--........................
XR_001716631.2 PREDICTED: Pan   563 ..C......--........................
XR_169790.4 PREDICTED: Pan tro  556 .........--..........T.TA..........
XM_004050337.3 PREDICTED: Gori  570 .........--........................
XR_002005707.2 PREDICTED: Gori  564 .........--........................
XM_004052942.3 PREDICTED: Gori  568 .........--..........T.T...........
XM_009245970.2 PREDICTED: Pong  561 .........--..........T.TA......C---
XR_002913425.1 PREDICTED: Pong  560 .......G.--..........T.TA..........
XR_656019.2 PREDICTED: Pongo a  561 .........--..........T.TA..........
XM_030801594.1 PREDICTED: Noma  564 .........--.....-------------------
XR_004026378.1 PREDICTED: Noma  563 .........--..........T.TA..........
XR_004027821.1 PREDICTED: Noma  563 .........--........................
XM_015113206.2 PREDICTED: Maca  564 ..CT.....--..........T..A..........
XM_001112566.4 PREDICTED: Maca  564 .........--..........T..A..........
XR_001438791.2 PREDICTED: Maca  573 .........--..........T.TA........AA
XM_012040930.1 PREDICTED: Cerc  564 .........--.........AT..A..........
XR_001017992.1 PREDICTED: Cerc  564 .........--.........AT..A..........
XR_001011714.1 PREDICTED: Cerc  564 .........--..........T..A..........
XM_010354836.2 PREDICTED: Rhin  565 .........--..........T..A.........-
XR_748909.2 PREDICTED: Rhinopi  565 .........--..........T..A..........
XM_030922644.1 PREDICTED: Rhin  565 .........--..........T..A..........
XM_039471018.1 PREDICTED: Saim  499 -----------------------------------
XM_039468571.1 PREDICTED: Saim  559 .--.TGG..--................C.C....A
XM_039478903.1 PREDICTED: Saim  565 .--.TGG..--................C.C....A
XM_012456047.2 PREDICTED: Aotu  561 .G.C..G..--..................C.....
XR_002477520.1 PREDICTED: Aotu  564 ....A....--..................C.....
XR_001106643.2 PREDICTED: Aotu  571 .........--..................C....A
XM_008053679.1 PREDICTED: Carl  320 -----------------------------------
XR_504221.2 PREDICTED: Carlito  550 .--.TGG.GGT............T...-------.
XM_003802780.3 PREDICTED: Otol  542 ..A..GC..--.............ACAC.C...A-
XR_001161573.1 PREDICTED: Otol  544 C.A..GG..--.............ACA..C...AA
```

Figure S10: Alignment of IFITM retrogene genome sequence with corresponding canonical clusters IR-IFITM mRNA sequence: Alignment of two randomly selected genome sequences of IFITM retrogenes of each species with the mRNA sequence of the corresponding canonical clusters IR-IFITM.
